# Supplementary material for: How has the COVID-19 pandemic affected the utilisation of online consultation and face-to-face medical treatment? An interrupted time-series study in Beijing, China
Source: BMJ Open. 2023 Feb 10;13(2):e062272. doi: 10.1136/bmjopen-2022-062272 (PMC9922877; doi:10.1136/bmjopen-2022-062272)
Supplement: Author's manuscript [file bmjopen-2022-062272.draft_revisions.pdf]

BMJ Open

BMJ Open is committed to open peer review. As part of this commitment we make the peer review history of every article we publish publicly available.

When an article is published we post the peer reviewers' comments and the authors' responses online. We also post the versions of the paper that were used during peer review. These are the versions that the peer review comments apply to.

The versions of the paper that follow are the versions that were submitted during the peer review process. They are not the versions of record or the final published versions. They should not be cited or distributed as the published version of this manuscript.

BMJ Open is an open access journal and the full, final, typeset and author-corrected version of record of the manuscript is available on our site with no access controls, subscription charges or pay-per-view fees (http://bmjopen.bmj.com).

If you have any questions on BMJ Open's open peer review process please email info.bmjopen@bmj.com

BMJ Open

How has the COVID-19 pandemic affected the utilization of online consultation and offline medical treatment? An interrupted time series study in Beijing, China.

Journal:	*BMJ Open*
Manuscript ID	bmjopen-2022-062272
Article Type:	Original research
Date Submitted by the Author:	23-Feb-2022
Complete List of Authors:	Zhang, Shan; Capital Medical University,<br>Ma, Chengyu; Capital Medical University
Keywords:	COVID-19, PUBLIC HEALTH, SOCIAL MEDICINE

SCHOLARONE™
Manuscripts

I, the Submitting Author has the right to grant and does grant on behalf of all authors of the Work (as defined in the below author licence), an exclusive licence and/or a non-exclusive licence for contributions from authors who are: i) UK Crown employees; ii) where BMJ has agreed a CC-BY licence shall apply, and/or iii) in accordance with the terms applicable for US Federal Government officers or employees acting as part of their official duties; on a worldwide, perpetual, irrevocable, royalty-free basis to BMJ Publishing Group Ltd ("BMJ") its licensees and where the relevant Journal is co-owned by BMJ to the co-owners of the Journal, to publish the Work in this journal and any other BMJ products and to exploit all rights, as set out in our licence.

The Submitting Author accepts and understands that any supply made under these terms is made by BMJ to the Submitting Author unless you are acting as an employee on behalf of your employer or a postgraduate student of an affiliated institution which is paying any applicable article publishing charge ("APC") for Open Access articles. Where the Submitting Author wishes to make the Work available on an Open Access basis (and intends to pay the relevant APC), the terms of reuse of such Open Access shall be governed by a Creative Commons licence – details of these licences and which Creative Commons licence will apply to this Work are set out in our licence referred to above.

Other than as permitted in any relevant BMJ Author's Self Archiving Policies, I confirm this Work has not been accepted for publication elsewhere, is not being considered for publication elsewhere and does not duplicate material already published. I confirm all authors consent to publication of this Work and authorise the granting of this licence.

For peer review only

How has the COVID-19 pandemic affected the utilization of online consultation and offline medical treatment? An interrupted time series study in Beijing, China.

Shan Zhang[1], Chengyu Ma[1]

[1] School of Public Health, Capital Medical University, Beijing, People's Republic of China

First Author: Shan Zhang, Master Degree Candidate, zsunll2017@163.com

Corresponding Author: Chengyu Ma, Associate Professor, 10132415@qq.com

Abstract

Objective The COVID-19 pandemic has had a major impact on healthcare utilization. The aim of this paper was to quantify how the utilization of healthcare services changed both online and offline during this time, so as to gain insight and inform planning of future healthcare resources during the outbreak of infectious diseases.

Design An interrupted time series study.

Setting We collected monthly healthcare service utilization data online and offline in 22 public hospitals in Beijing in 2019 and 2020.

Participants 32,421,515 offline outpatient and emergency events for 2019 and 20,642,104 for 2020 in 22 public hospitals in Beijing. 1,041,190 offline discharges events for 2019 and 668,997 for 2020. And 70,086 online consultation events for 2019 and 144,651 for 2020.

Results The monthly average outpatient visits and discharges decreased by 36.33% and 35.75%, respectively, compared with 2019 in 22 public hospitals in Beijing. At the same time, the monthly average online consultations increased by 90.06%. There was a highly significant reduction in the mean outpatients and inpatients, which dropped by 1755930 cases (P<0.01) and 59.20 thousand cases (P<0.01), respectively. Online consultations rose by 3650 cases (P<0.05). We identified an immediate and significant drop in offline healthcare services for four major diseases, i.e. acute myocardial infarction (-174.405, P<0.1), lung cancer (-2502.02, P<0.01), disk disease (-375.560, P<0.01), and Parkinson's disease (-205.229, P<0.01). Otherwise, a significant increase in online consultations was shown for disk disease (63.118, P<0.01) and Parkinson's disease (24.625, P<0.05). There were more than 1,300 unique physicians providing online consultation services per month in 2020, which was 35.3% higher than in 2019.

Conclusions There were obvious complementary trends in online and offline healthcare services during the COVID-19 pandemic. Different changes in healthcare utilization were shown for different diseases. Non-critically ill patients chose online consultation immediately after the COVID-19 lockdown, but critically ill patients chose hospital healthcare services first. At the same time, the volume of online physician service significantly rose as a result of COVID-19.

Keywords COVID-19, healthcare utilization, online consultation, health services research

Strengths and limitations of this study

- Little data is available the relationship between hospital visits and online health services over the COVID-19 pandemic, especially with quantitative time-trend evidence. It is valuable to examine the short- and long-term pattern of the changes in hospital volume and online health services during the COVID-19 pandemic.

For peer review only

- The study compared the impact of the COVID-19 pandemic on online and offline healthcare service utilization.
- The behavior of patients with five diseases were selected in this paper. Further studies need to determine whether this phenomenon can be extended to other diseases.

Introduction

In early 2020, the COVID-19 pandemic affected most of the planet and rapidly changed the utilization of healthcare services worldwide[1]. Fear of infection and reduced availability of healthcare services has led to a decrease in non-COVID healthcare utilization in hospitals. Rapid and innovative deployment of telemedicine and online health services has happened in many countries to relieve the pressure on hospitals and to prevent cross-infection. In the United States, emergency room (ER) visits dropped by 42% during, April followed by a 26% reduction at the end of May, compared with 2019[2]. Meanwhile, telemedicine visits increased from 102.4 daily to 801.6 daily between March 2nd and April 14th 2020[3].

It is clear that the impact of the global pandemic on healthcare systems has been acute. As the COVID-19 pandemic continues, many studies have reported major changes in the utilization of healthcare services because of measures such as lockdowns and stay-at-home orders. These changes include large reductions in hospital visits, and some selective increases in online health services[4,5]. However, little data is available the relationship between hospital visits and online health services over the same time period, especially with quantitative time-trend evidence. It is valuable to examine the short- and long-term pattern of the changes in hospital volume and online health services during the COVID-19 pandemic. The extent and effects of substitution with online healthcare services for different kinds of disease also require further investigation.

Beijing is a megacity with the most extensive internet accessibility (77.8%)[6] and most famous hospitals compared to other parts of China. After the COVID-19 pandemic, many patients were unable to access hospitals in Beijing due to the lockdown policies; in response, online health services and an internet-based health system have been utilized by hospitals in addition to third-party service platforms, such as GoodDoctor (www.haodf.com). Physicians are active in spending their free time on these platforms, to provide patients with high-quality online health consultations. Online consultations refer to patients using the Internet to communicate with physicians in real time through online text, pictures, video, audio, etc. This study illustrates the speed, scale, and reception of online consultations utilization by patients and physicians, and the complementary trends in online and offline health services. The results may help health systems optimize the post-pandemic use of health resources.

Methods

Data collection

Monthly hospital-grade level healthcare service data from January 2019 to December 2020 were provided by 22 municipal tertiary first-class public hospitals in Beijing. Beijing municipal hospitals refer to the public hospitals managed by the Beijing Municipal Health Commission.

Firstly, outpatient and emergency visits, as well as the number of discharges were used as the overall offline healthcare utilization outcomes. Four diseases including acute myocardial infarction, lung cancer, disk disease, and Parkinson's disease were selected to analyze changes in healthcare service utilization for different disease severities. Due to data sharing restrictions, the

For peer review only - http://bmjopen.bmj.com/site/about/guidelines.xhtml

monthly number of discharges for these four diseases were collected from July 2019 to December 2020.

Secondly, online consultations data were obtained from GoodDoctor (www.haodf.com). GoodDoctor is the largest and longest-running online physician-patient communication platform in China. It was founded in 2006. As of October 2021, it has collected information on 860,000 physicians from 9,780 regular hospitals in China, of which more than 73% are physicians in tertiary hospitals. A large number of patients turned to this platform for help after the COVID-19 pandemic outbreak, which largely represents the trend in the utilization of online consultations during the pandemic.

The platform provides historical data of online consultations from 2010 to the present time. We collected the daily online consultation data of the abovementioned 22 public hospitals in 2019 and 2020, and screened out the online consultation volume data for acute myocardial infarction, lung cancer, disk disease, and Parkinson's disease based on disease information. Figure 1 shows the content of the online consultation provided by this platform. We used the Java language to design a web crawler to capture daily online consultation information from web pages. Specific extracted fields include date, website link, title, disease description, disease name, expected treatment, the duration of illness, consultation text of the patient and physician, and the name, title, department, patient voting numbers, online consultation volume, and satisfaction of online service physicians. The final sample size collected was 70,086 for 2019 and 144,651 for 2020.

Study design

We conducted an interrupted time series analysis to estimate changes in offline and online healthcare utilization before and after the COVID-19 outbreak. Simple pre-post designs perform before-after comparisons by estimating the change from a single pre-intervention time point to a single post-intervention timepoint. However, these have poor internal validity as they cannot exclude underlying trends as a cause for any change[7]. Conversely, interrupted time series (ITS) use multiple observations in the pre-intervention and post-intervention periods, and thus offers a quasi-experimental research design with a potentially high degree of internal validity[8,9]. To do so, we fitted an ordinary least squares (OLS) model using either regress or Newey (with lag specified) with monthly service volume as the primary outcome of interest. We produced Newey-West standard errors to handle the autocorrelation.

We treated COVID-19 as the intervention, and 2019 and 2020 as the pre-intervention and post-intervention periods, respectively. The ITSA regression model assumes the following form:

$$Y_t = \beta_0 + \beta_1 T_t + \beta_2 X_t + \beta_3 X_t T_t + \varepsilon_t$$

Y_t is the effect variable, including outpatient and emergency visits, discharge volume, and online consultation volume. T_t is the time since the start of the study, X_t is a dummy variable representing the intervention (pre-intervention period 0, otherwise 1), and X_tT_t is the interaction term. β_0 represents the intercept or starting level of the outcome variable. β1 is the slope or trajectory of the outcome variable until the COVID-19 outbreak. β_2 represents the change in the level of the outcome that occurred in the period immediately following the COVID-19 outbreak (compared with the counterfactual). β_3 represents the difference between prepandemic and pandemic slopes of the outcome. Thus, we looked for significant p-values in β_2 to indicate an immediate treatment effect, or in β_3 to indicate a treatment effect over time [10].

For peer review only

In addition, in order to know the impact of the COVID-19 on the utilization of healthcare services for different diseases, we stratified models by disease type restricted to: (1) acute myocardial infarction, (2) lung cancer, (3) disk disease, and (4) Parkinson's disease. All statistical analyses were conducted using Stata 15.0 and SPSS 20.0.

Patient and Public Involvement

This paper was an observational cohort study using publicly available data from the GoodDoctor. We did not use private patient-level information. Our study passed the ethics review of Ethics Committee of Capital Medical University.

Result

1. COVID-19 pandemic trends in Beijing

Figure 2 depicts the epidemic trends and significant changes in the COVID-19 outbreak in Beijing. The first confirmed case of COVID-19 in Beijing occurred on January 20, 2020. Since then, the virus spread across Beijing. Beijing launched the first-level response mechanism for public health emergencies on January 24, 2020. In order to respond to public health emergencies in a timely manner, China has launched a tiered response mechanism based on different pandemic levels and hazards. The first-level response mechanism is unified deployment by provincial headquarters according to the decisions of The State Council; the second-level response mechanism refers to deployment by the provincial headquarters; the third-level response mechanism refers to deployment by the headquarters of the prefect-level city. There were two peaks on February 2 and March 23 in the early days. After that, the epidemic entered a normalization stage, with no new cases for a long time. On June 11, a 52-year old man was diagnosed with COVID-19 from Xinfadi Market in Beijing; after a 56-day zero new case interval, marking the beginning of 'the second epidemic', new cases increased sharply on June 13, and the number of new cases in a single day reached the highest value of 36 cases. Beijing's response mechanism for public health emergencies was adjusted from level 3 to level 2 on June 16. This led to a fast downward slope with the control of the Chinese government. The epidemic in Beijing has gradually stabilized since then. As of December 31, 987 confirmed cases and 9 deaths had been reported.

2. **Impact of COVID-19 pandemic on utilization of healthcare services**

In Table 1, We show the descriptive statistical indicators of the offline outpatient and emergency visits, discharges, and online consultations in 2019 and 2020. Compared with 2019, the utilization of offline healthcare services decreased in 2020. The monthly average outpatient and emergency visits, as well as discharges decreased by 36.33% and 35.75%, respectively. Compared with 2019, the utilization of online consultations increased in 2020. The monthly average online consultations increased by 90.06%.

Table 1 The healthcare service utilization in 2020 compared with 2019 (ten thousands)

	2019 (before the COVID-19 outbreak)			2020 (after the COVID-19 outbreak)		
	offline outpatient and emergency visits	offline discharges	online consultation	offline outpatient and emergency visits	offline discharges	online consultation

For peer review only

Mean		270.179	8.677	0.634	172.018	5.575	1.205
Std.		25.779	0.918	0.054	54.554	2.160	0.238
Median		274.391	8.956	0.611	179.315	5.996	1.272
Min		201.617	6.190	0.575	69.529	1.959	0.550
Max		311.549	9.559	0.754	243.728	8.292	1.450
Quartile	Q1	261.232	8.328	0.592	135.299	3.959	1.189
	Q3	280.498	9.215	0.672	219.232	7.656	1.324

Table 2 shows the percentage changes in healthcare service utilization in Beijing. Compared with the same period in 2019, the monthly offline outpatient and emergency visits, as well as discharges in 2020 were lower. The percentage change frequency for both was greatest in March, with decreases of 66.97% and 75.17%, respectively. The overall change showed a decreasing trend after that. It should be noted that, when affected by "the second epidemic", offline healthcare services increased slightly again, but utilization in July fell at a higher rate than in June. Online consultations in all months except January were higher than the same period in 2019. The overall change showed an increasing trend. The greatest decrease was seen in August. Figure 3 shows that, in the early days of the pandemic (January to June), the utilization of online and offline healthcare services showed obvious complementary trends. Afterwards, the growth rate of online consultations has remained at a relatively high level, while the decline rate of offline healthcare services has gradually decreased. This suggests that the utilization of offline healthcare services had gradually returned to normal levels as COVID-19 fades away, while online consultations still maintain a high growth rate.

Table 2 Percentage change in healthcare service utilization in 2020 compared with 2019 (%)

Month	offline outpatient and emergency visits	offline discharges	online consultation
January	-22.31	-12.46	-20.81
February	-65.51	-68.35	64.89
March	-66.97	-75.17	68.76
April	-54.50	-59.63	75.90
May	-41.69	-46.89	86.61
June	-35.65	-29.60	97.97
July	-44.72	-50.39	91.89
August	-30.09	-30.54	139.85
September	-19.17	-13.78	124.22
October	-23.69	-21.20	126.73
November	-18.08	-16.05	138.85
December	-21.77	-13.25	113.45
Total	-36.33	-35.75	90.12

3. Interrupted time-series analysis

Changes in overall online and offline healthcare services

As shown in Table 3, there was a highly significant reduction in the mean number of outpatient and emergency visits, as well as discharges, which dropped by 1755930 cases (P<0.01) and 59200 cases (P<0.01), respectively. Before the COVID-19 outbreak, there was a gentle

upward trend in offline healthcare services (including outpatient and emergency visits and discharges, Figure 4). Soon after the COVID-19 outbreak, there was a significant increase in the slope of this trend line, but the trend was not statistically significant. At the same time, online consultations rose by 3650 cases (P<0.05), followed by a significant increase in the monthly trend of utilization (relative to the pre-pandemic) of 580 cases.

Table 3 The impact of COVID-19 on the utilization of online and offline healthcare services (ten thousand)

	Medical utilization reduction after the COVID-19 outbreak (intercept)	P value	Average monthly medical utilization decline after the COVID-19 outbreak (potential trend)	P value
offline outpatient and emergency visits	-175.593 (27.247*)	<0.01	7.357 (4.454)	>0.1
offline discharges	-5.920 (1.608)	<0.01	0.283 (0.217)	>0.1
online consultation	0.365 (0.143)	<0.05	0.058 (0.019)	<0.01

*Standard errors.

Changes in offline healthcare services by type of disease

Table 4 summarizes the effect of COVID-19 on offline healthcare services for different diseases. The COVID-19 outbreak was associated with a rapid decline in offline healthcare services for different diseases. From the perspective of immediate effect, we identified a significant drop in healthcare services for four diseases (-174.405 acute myocardial infarction, -2502.02 lung cancer, -375.560 disk disease, -205.229 Parkinson's disease). From the perspective of long-term effects, acute myocardial infarction, lung cancer, and disk disease services showed a non-significant stepwise increase (the slope of the trend line increased). Simultaneously, there was a significant increase in the monthly trend of utilization for Parkinson's disease (relative to the pre-pandemic period) of 12.669 cases (Figure 5).

Table 4 The impact of COVID-19 on the utilization of offline healthcare services by type of disease

	Medical utilization reduction after the COVID-19 outbreak (intercept)	P value	Average monthly medical utilization decline after the COVID-19 outbreak (potential trend)	P value
acute myocardial infarction	-174.405 (84.060*)	<0.1	12.438 (11.540)	>0.1
lung cancer	-2502.02 (367.118)	<0.01	80.508 (80.480)	>0.1
disk disease	-375.560 (72.369)	<0.01	29.163 (16.876)	>0.1
Parkinson's disease	-205.229 (47.852)	<0.01	12.669 (4.892)	<0.05

*Standard errors.

Changes in online consultations by type of disease

Table 5 summarizes the effect of COVID-19 on online consultations for different diseases. The COVID-19 outbreak was associated with a rapid rise in online consultations for different diseases. From the perspective of immediate effect, we identified a significant increase in

For peer review only

healthcare services for disk disease and Parkinson's disease, by 63,118 and 24,625 cases, respectively. On the contrary, online consultations for acute myocardial infarction and lung cancer showed a small, non-significant decrease. From the perspective of long-term effects, online consultations for acute myocardial infarction, lung cancer and disk disease increased monthly at a rate of 1,633, 14,360, and 3,402 respectively after COVID-19 outbreak (Figure 6).

Table 5 The impact of COVID-19 on the utilization of online consultations by type of disease

	Medical utilization reduction after the COVID-19 outbreak (intercept)	P value	Average monthly medical utilization decline after the COVID-19 outbreak (potential trend)	P value
acute myocardial infarction	-5.998 (3.511*)	>0.1	1.633 (0.472)	<0.01
lung cancer	-11.832 (19.413)	>0.1	14.360 (3.079)	<0.01
disk disease	63.118 (11.955)	<0.01	3.402 (1.851)	<0.1
Parkinson's disease	24.625 (10.349)	<0.05	1.772 (1.335)	>0.1

*Standard errors.

4. **Impact of COVID-19 pandemic on online service physicians volume**

Affected by COVID-19, the monthly volume of online services provided by physicians in 2020 was significantly higher than in 2019, except for January. There were, on average, more than 1,300 unique physicians from 22 hospitals in Beijing providing online consultation services per month in 2020, 35.3% higher than in 2019. This suggests that the supply of online consultations has greatly increased after the COVID-19 outbreak (Figure 7).

Discussion

Based on monthly treatment data from 22 public hospitals in Beijing, there were obvious complementary trends in online and offline healthcare services during the COVID-19 pandemic. We found that public hospitals in Beijing experienced substantial losses in offline healthcare and substantial increases in online consultation services when the COVID-19 pandemic began in China in 2020. The interrupted time series models revealed that, after the COVID-19 outbreak, the considerable reduction in offline healthcare services was a short-term effect, while the increase in online consultation was a long-term effect. Simultaneously, the effects of COVID-19 on healthcare services for different diseases were different. Non-critical disease patients showed greater reductions in offline service utilization, and chose online consultations immediately after the COVID-19 lockdown. However, critical disease patients chose offline treatment first, and slowly moved online over time. On the other hand, the volume of online services provided by physicians increased significantly as a result of COVID-19.

Our study is consistent with previous reports on hospital healthcare service changes during the COVID-19 pandemic[11,12]. A systematic review from UK analyzed 81 studies and reported that the utilization of healthcare in care facilities sharply decreased by about a third during the pandemic period, with greater reductions among people with less severe illness[11]. Additionally, according to a study from one large health system (NYU Langone Health) in the United States, telemedicine visits increased from 102.4 daily to 801.6 daily between March 2 and April 14 2020, with a concomitant decline in in-person visits by over 80%. Furthermore, we showed that online

For peer review only

consultation underwent sustained growth due to the impact of COVID-19, while offline healthcare services showed a short-term decline.

A rapid decrease in overall offline discharge admissions was observed for four different diseases across 22 tertiary public hospitals in Beijing after the COVID-19 lockdown. A study performed in Germany observed a similar reduction in traditional face-to-face healthcare services for different diseases[13]. That study found that hospital admissions for malignant cancer treatments and acute myocardial infarction were reduced after the lockdown announcement. However, a difference is that our analysis also showed the impact of the epidemic on diseases with different disease severity. Furthermore, we also examined the changes in online consultations for different diseases. We found that different diseases showed different online change trends. Online consultations for critical illnesses (such as acute myocardial infarction, lung cancer) did not increase immediately after the outbreak, but gradually increased in the long-term study. In contrast, we observed a considerable and immediate increase in non-critical clinical situations (such as disk disease and Parkinson's disease), and this trend has been maintained.

Several reasons may explain the dramatic decrease in offline healthcare services after the COVID- 19 pandemic. Firstly, the lockdown policy and population mobility control measures were implemented in China to mitigate the spread of the pandemic. For instance, the community implemented closed management, hospital services were limited, non-emergency services had to be reserved in advance and staggeredly used in Beijing during the pandemic period. This might have reduced the availability and accessibility of healthcare services offline. Secondly, some patients chose to postpone their treatment to prevent cross-infection in hospital. Most healthcare resources were relocated to manage the pandemic. This might trigger attitudes towards delaying less urgent cases by both the patient and the healthcare system. Thirdly, some health demands might be eliminated. For instance, many patients with self-resolving diseases did not choose to get treatment face to face, such as for colds, diarrhea etc. The more important point is that some health demands might be transferred online. Abundant internet diagnosis and treatment services were supplied by medical institutions in Beijing during the pandemic, such as online consultation, instant messaging, synchronous chat, video-conferencing, etc. Online consultation can span time and space. The physician and patient do not have to meet each other within a physical space, subsequently decreasing the requirement for offline travel, appointment-related concerns, and the risk of offline infection. Some patients could migrate to online consultation to meet their health needs. That is why the volume of online consultations has increased significantly.

On the one hand, our results indicate that a mass migration to online consultations took place shortly after the COVID-19 pandemic. On the other hand, the migration of critically ill patients was slower than for non-critically ill patients, because they relied more on facility treatment. For example, the efficiency of the care pathway at every step matters very much for acute myocardial infarction patients[14]. Chemotherapy (CT), radiation therapy (RT), surgery, and molecular targeted therapy are keystones in the treatment of early and locally advanced lung cancer (LC) with good prognosis[15]. These rely on offline operations and inspections. Online consultations cannot provide these services. This is why the online consultation volume for critical illnesses did not increase immediately after the outbreak. In addition, we found that the online consultation volume for three diseases, except for Parkinson's, had a continuously

For peer review only

increasing trend. This phenomenon can be explained by the fact that the COVID-19 pandemic accelerated the popularity of online healthcare, and the awareness and acceptance of the public regarding online healthcare has increased.

In addition, our study found that the pandemic not only led to a significant increase in online patients, but also facilitated the online migration of physicians. The number of physicians offering services online in 2020 was much higher than over the same period in 2019, except for January. This shows that physicians pay attention to providing online healthcare services during their leisure time as well, to meet the balance of medical resources between supply and demand during and after the pandemic.

With the COVID-19 pandemic, the supply of offline healthcare services was relatively insufficient, resulting in partial healthcare service disruption. The COVID-19 pandemic resulted in a decrease in the supply of offline healthcare services, which was part of the healthcare service disruption. To reduce the public health impact, we put forward two suggestions: firstly, we should designate some part of healthcare resources to online provision. Moreover, we should rapidly set up a collaborative model of offline and online healthcare service in public health emergency situations, to meet the demand of patients. Online consultations and telemedicine can alleviate both geographic and physician shortages, expand healthcare accessibility, and improve the quality of healthcare provision. Therefore, online healthcare services can serve as an important supplement to traditional healthcare services, optimize health resources allocation, and improve service efficiency, especially for non-critically ill patients.

However, not all diseases are suitable for online consultations. Offline healthcare services are necessary and irreplaceable, such as physical exams, diagnosis, and the treatment of difficult and severe diseases. Different healthcare support measures should be formulated according to the characteristics of different diseases. For critical cases, offline channels ought to be opened for diagnosis and treatment, to ensure that patients can receive offline treatment in a timely manner. There are studies indicating that 9.1% patients experienced a delay in lung cancer treatment and half of Acute Myocardial Infarction(AMI) patients did not reach out to the hospital at all during the COVID-19 pandemic[14,16]. For critical cases, delaying or stopping treatment can have a significant negative impact on the physical and mental health of critically ill patients. A timely diagnosis and improved treatment strategy will facilitate the health of patients[15]. For mild cases, co-operation among the government, health facility and third-party platforms should be enhanced. Online healthcare services such as telemedicine, online consultation, psychological counseling, and chronic disease revisits ought to be expanded. Expanding the supply of online healthcare resources and helping patients migrate to online to receive healthcare services are crucial.

Finally, our work confirms that the increase in online consultations is a persistent phenomenon. A new normal deployment of online healthcare services happened in a pandemic and policy-enabling environment. The development of online consultation should be encouraged in view of the advantages of online consultation across time and space. New technologies will be fully exploited to online consultation, such as 5G technology, sensing techniques, and artificial intelligence. It is important to innovate the long-term synergistic modalities of online and offline healthcare services and to promote the connection of high-quality medical resources at all levels.

Conclusion

For peer review only

Offline healthcare services dropped precipitously and online consultations rose sharply in 22 tertiary public hospitals in Beijing during the COVID-19 pandemic. However, offline healthcare services showed quite a fast recovery and online consultations showed continued growth during the post-pandemic period. The change trend in online consultation volume varied by disease, which was characterized by a non-significant increase in the short-term and a significant increase in the long-term regarding critical cases after the outbreak, as well as a significant increase in non-critical cases in the long short-term. The availability of online consultations should be encouraged during pandemics. Particular attention should be given to severely ill patients who need to obtain offline hospital care adequately and in a timely manner.

Limitations

This study has several limitations. First of all, we studied the complementary trend of online and offline health services utilization, but did not analyze the impact of the epidemic on the quality of healthcare services. Secondly, in order to observe the changes in healthcare service utilization, the behavior of patients with diseases of different severity, i.e. acute myocardial infarction, lung cancer, disk disease, Parkinson's disease were selected in this paper. Further studies need to determine whether this phenomenon can be extended to other diseases.

Author affiliations

[1] School of Public Health, Capital Medical University, Beijing, People's Republic of China

Contributors The research idea originated with CM, but all authors designed the study. SZ directed the data analyses. CM and SZ co-writed this manuscript.

Funding The authors have not declared a specific grant for this research from any funding agency in the public, commercial or not- for- profit sectors.

Competing interests We have read and understood the BMJ policy on declaration of interests. All authors report no competing interests.

Patient and public involvement statement Patients and the public were not involved in the design or conduct of this study. The study used publicly available data from the GoodDoctor, which has signed privacy policies with users.

Patient consent for publication Not applicable.

Ethics approval This project was unanimously approved by the expert group of Capital Medical University Ethics Committee, ethical approval document NO.Z2020SY125.

Provenance and peer review Not commissioned; externally peer reviewed.

Data availability statement Data are available on reasonable request.

For peer review only

References

1 Moynihan R , Sanders S , Michaleff Z A , et al. Impact of COVID-19 pandemic on utilisation of healthcare services: a systematic review[J]. BMJ Open, 2021, 11(3):e045343.

2 Garcia S , Albaghdadi M S , Meraj P M , et al. Reduction in ST-Segment Elevation Cardiac Catheterization Laboratory Activations in the United States During COVID-19 Pandemic[J]. Journal of the American College of Cardiology, 2020.

3 Mann D M , Ji C , Rumi C , et al. COVID-19 transforms health care through telemedicine: Evidence from the field[J]. Journal of the American Medical Informatics Association(7):7.

4 Chen Y, Cai M, Li Z, Lin X, Wang L. Impacts of the COVID-19 Pandemic on Public Hospitals of Different Levels: Six-Month Evidence from Shanghai, China. Risk Manag Healthc Policy. 2021;14:3635-3651.

5 Gang L , Guorui F , Yanyan C , et al. What patients "see" doctors in online fever clinics during COVID-19 in Wuhan?[J]. Journal of the American Medical Informatics Association(7):7.

6 China Internet Network Information center. Beijing, China: CNNIC, 2021. Annual Report 2021.

7 James, Lopez, Bernal, et al. The use of controls in interrupted time series studies of public health interventions.[J]. International Journal of Epidemiology, 2018.

8 Shadish W, Cook T, Campbell D. Experimental and Quasi-Experimental Designs For Generalized Causal Inference. Houghton Mifflin, 2002.

9 Campbell, D. T., and J. C. Stanley. 1966. Experimental and Quasi-Experimental Designs for Research. Chicago, IL: Rand McNally.

10 Linden, A., and J. L. Adams. 2011. Applying a propensity-score based weighting model to interrupted time series data: Improving causal inference in program evaluation.Journal of Evaluation in Clinical Practice 17: 1231–1238.

11 Moynihan R, Sanders S, Michaleff ZA, Scott AM, Clark J, To EJ, Jones M, Kitchener E, Fox M, Johansson M, Lang E, Duggan A, et al. Impact of COVID-19 pandemic on utilisation of healthcare services: a systematic review. BMJ Open. 2021 Mar 16;11(3):e045343.

12 Mann DM, Chen J, Chunara R, et al. COVID-19 transforms health care through telemedicine: Evidence from the field. J Am Med Inform Assoc. 2020 Jul 1;27(7):1132-1135.

13 Kapsner LA, Kampf MO, Seuchter SA, et al. Reduced Rate of Inpatient Hospital Admissions in 14 German University Hospitals During the COVID-19 Lockdown. Front Public Health. 2021 Jan 13;8:594117.

15 De Rosa S, Spaccarotella C, Basso C, et al. Reduction of hospitalizations for myocardial infarction in Italy in the COVID-19 era. Eur Heart J. 2020 Jun 7;41(22):2083-2088.

16 Sha Z, Chang K, Mi J, Liang Z, et al. The impact of the COVID-19 pandemic on lung cancer patients. Ann Palliat Med. 2020 Sep;9(5):3373-3378.

17 Fujita K, Ito T, Saito Z, et al. Impact of COVID-19 pandemic on lung cancer treatment scheduling. Thorac Cancer. 2020 Oct;11(10):2983-2986.

Figure legends

Figure 1 The information of the online consultation provided by this platform

Figure 2 COVID-19 epidemic trend in Beijing

For peer review only

Figure 3 Percentage change in healthcare service utilization in 2020 compared with 2019 (%)

Figure 4 The effect of COVID-19 intervention on offline and online healthcare utilization. Scatter plots represent monthly medical utilization. The black line of best fit represents medical utilization across estimated by postregression. The vertical black line is the intervention point. (a) offline outpatient and emergency visits; (b) offline discharges; (c) online consultation.

Figure 5 The effect of COVID-19 intervention on offline healthcare utilization by type of disease. Scatter plots represent medical utilization monthly. The black line of best fit represents medical utilization across estimated by postregression. The vertical black line is the intervention point. (a) Acute myocardial infarction; (b) lung cancer; (c) disk disease; (d) Parkinson's disease.

Figure 6 The effect of COVID-19 intervention on online consultations utilization by type of disease. Scatter plots represent medical utilization monthly. The black line of best fit represents medical utilization across estimated by postregression. The vertical black line is the intervention point. (a) Acute myocardial infarction; (b) lung cancer; (c) disk disease; (d) Parkinson's disease.

Figure 7 Changes in online physician volume in 2020 compared with 2019 (%). The dotted line is the mean.

For peer review only

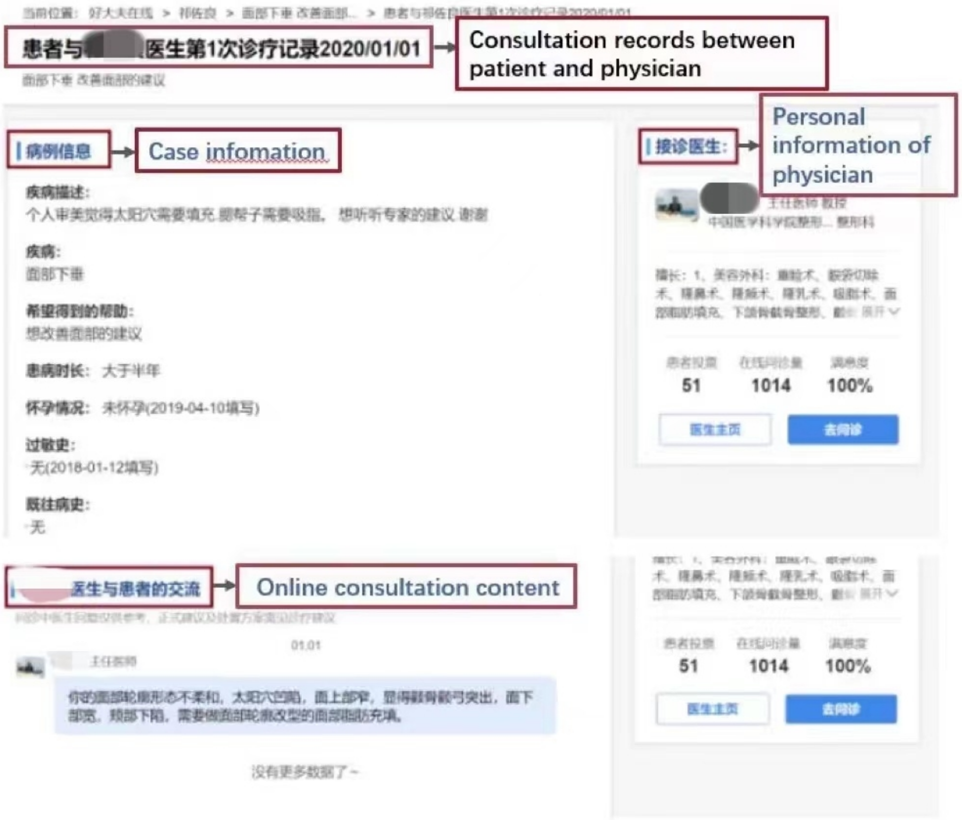

Figure 1 The information of the online consultation provided by this platform

361x309mm (72 x 72 DPI)

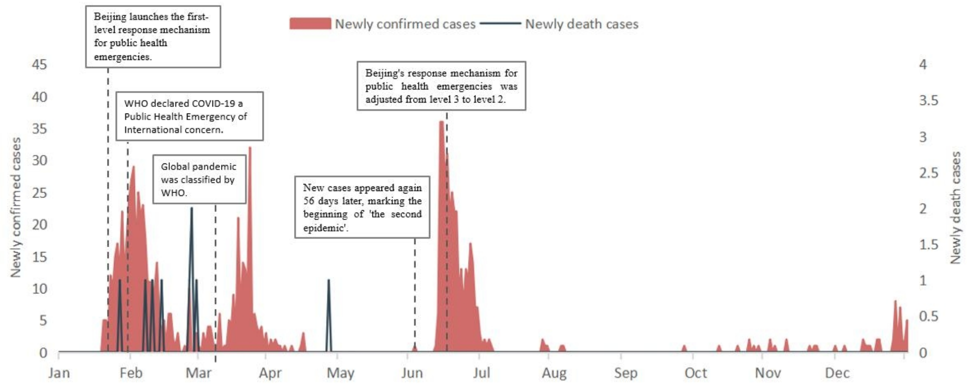

Figure 2 COVID-19 epidemic trend in Beijing

215x96mm (144 x 144 DPI)

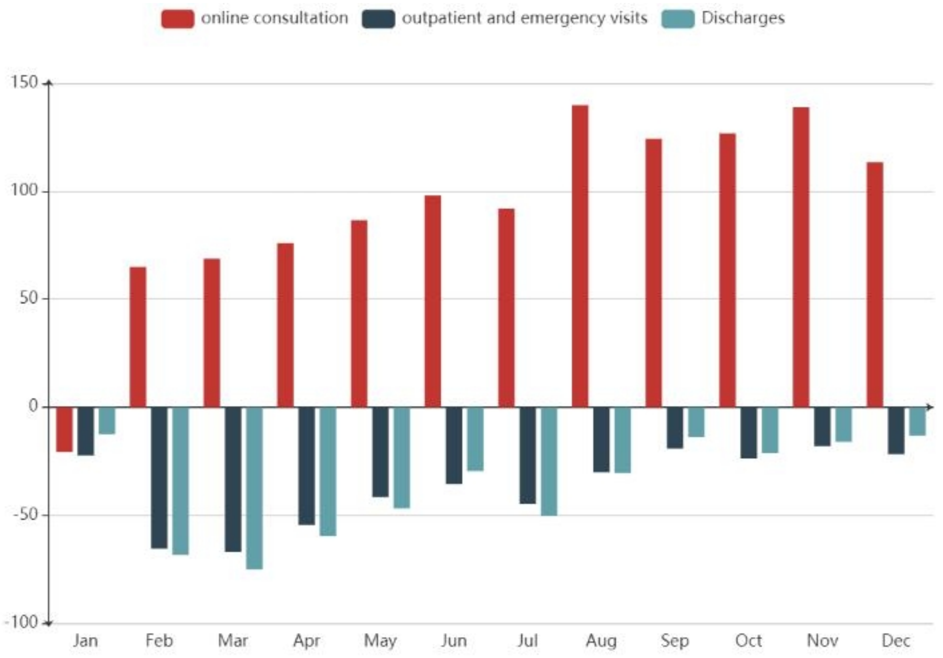

Figure 3 Percentage change in healthcare service utilization in 2020 compared with 2019 (%)

131x88mm (144 x 144 DPI)

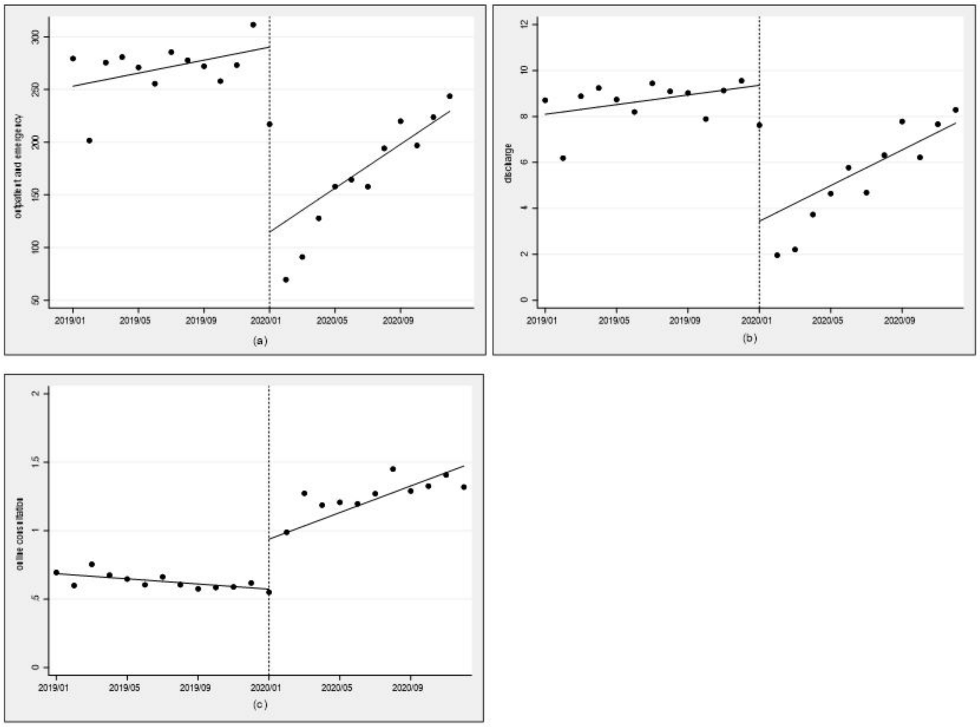

Figure 4 The effect of COVID-19 intervention on offline and online healthcare utilization. Scatter plots represent monthly medical utilization. The black line of best fit represents medical utilization across estimated by postregression. The vertical black line is the intervention point. (a) offline outpatient and emergency visits; (b) offline discharges; (c) online consultation.

149x111mm (144 x 144 DPI)

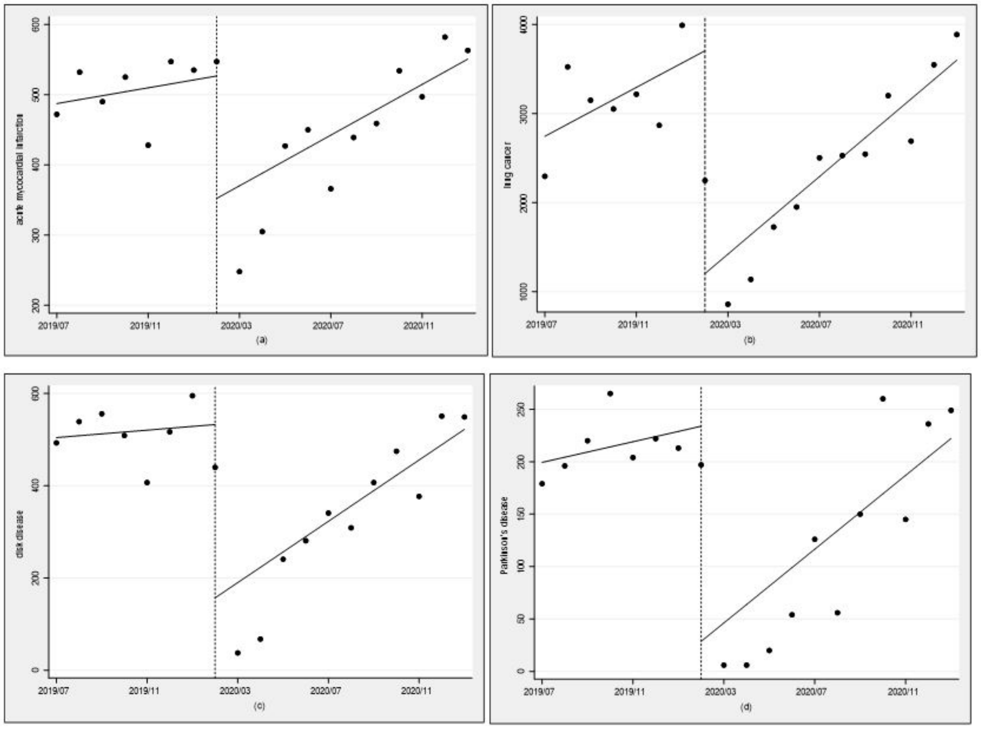

Figure 5 The effect of COVID-19 intervention on offline healthcare utilization by type of disease. Scatter plots represent medical utilization monthly. The black line of best fit represents medical utilization across estimated by postregression. The vertical black line is the intervention point. (a) Acute myocardial infarction; (b) lung cancer; (c) disk disease; (d) Parkinson's disease.

149x112mm (144 x 144 DPI)

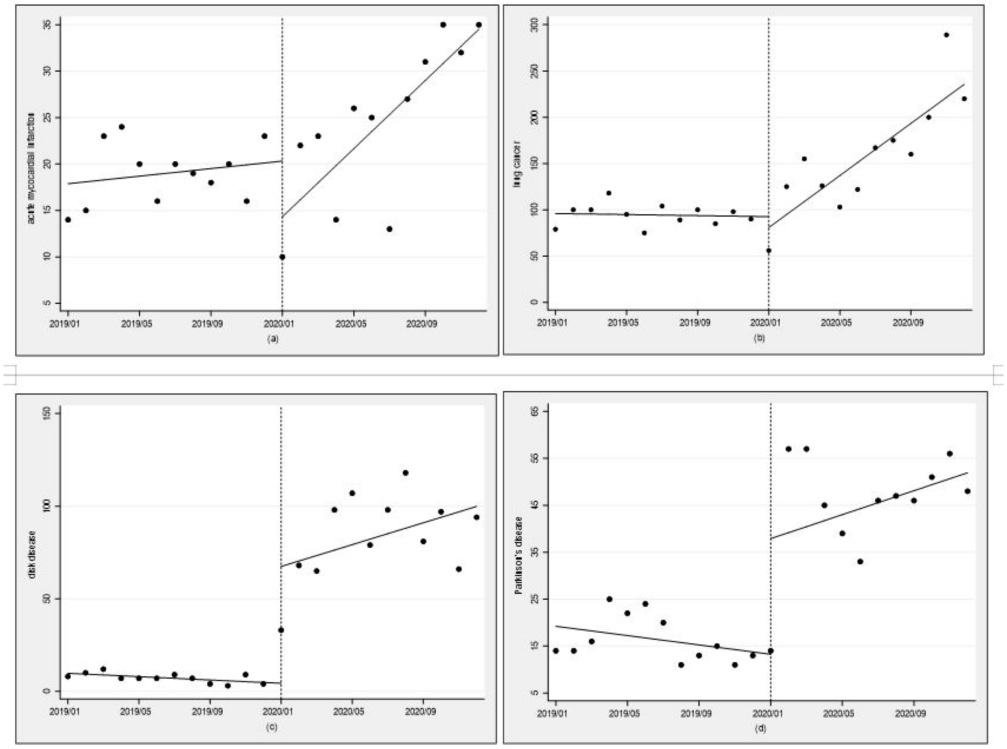

Figure 6 The effect of COVID-19 intervention on online consultations utilization by type of disease. Scatter plots represent medical utilization monthly. The black line of best fit represents medical utilization across estimated by postregression. The vertical black line is the intervention point. (a) Acute myocardial infarction; (b) lung cancer; (c) disk disease; (d) Parkinson’s disease.

149x115mm (144 x 144 DPI)

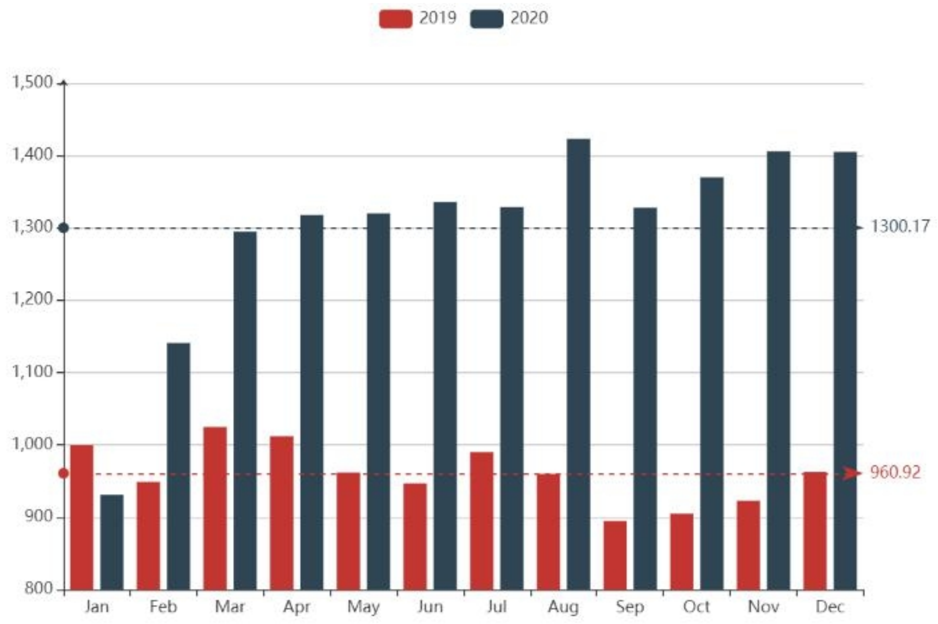

Figure 7 Changes in online physician volume in 2020 compared with 2019 (%). The dotted line is the mean.

131x88mm (144 x 144 DPI)

BMJ Open

How has the COVID-19 pandemic affected the utilization of online consultation and face-to-face medical treatment? An interrupted time-series study in Beijing, China.

Journal:	*BMJ Open*
Manuscript ID	bmjopen-2022-062272.R1
Article Type:	Original research
Date Submitted by the Author:	07-Dec-2022
Complete List of Authors:	Zhang, Shan; The Seventh Affiliated Hospital Sun Yat-sen University<br>Ma, Chengyu; Capital Medical University
**Primary Subject Heading**:	Public health
Secondary Subject Heading:	Health policy, Medical management, Public health
Keywords:	COVID-19, PUBLIC HEALTH, SOCIAL MEDICINE

SCHOLARONE™
Manuscripts

I, the Submitting Author has the right to grant and does grant on behalf of all authors of the Work (as defined in the below author licence), an exclusive licence and/or a non-exclusive licence for contributions from authors who are: i) UK Crown employees; ii) where BMJ has agreed a CC-BY licence shall apply, and/or iii) in accordance with the terms applicable for US Federal Government officers or employees acting as part of their official duties; on a worldwide, perpetual, irrevocable, royalty-free basis to BMJ Publishing Group Ltd ("BMJ") its licensees and where the relevant Journal is co-owned by BMJ to the co-owners of the Journal, to publish the Work in this journal and any other BMJ products and to exploit all rights, as set out in our licence.

The Submitting Author accepts and understands that any supply made under these terms is made by BMJ to the Submitting Author unless you are acting as an employee on behalf of your employer or a postgraduate student of an affiliated institution which is paying any applicable article publishing charge ("APC") for Open Access articles. Where the Submitting Author wishes to make the Work available on an Open Access basis (and intends to pay the relevant APC), the terms of reuse of such Open Access shall be governed by a Creative Commons licence – details of these licences and which Creative Commons licence will apply to this Work are set out in our licence referred to above.

Other than as permitted in any relevant BMJ Author's Self Archiving Policies, I confirm this Work has not been accepted for publication elsewhere, is not being considered for publication elsewhere and does not duplicate material already published. I confirm all authors consent to publication of this Work and authorise the granting of this licence.

For peer review only

How has the COVID-19 pandemic affected the utilization of online consultation and face-to-face medical treatment? An interrupted time-series study in Beijing, China.

[1] The Seventh Affiliated Hospital, Sun Yat-sen University, Guangdong, China

[2] School of Public Health, Capital Medical University, Beijing, China

First Author: Shan Zhang, Master's Degree, zsunll2017@163.com

Corresponding Author: Chengyu Ma, Associate Professor, 10132415@qq.com

Abstract

Objective The COVID-19 pandemic has had a major impact on healthcare utilization. This study aimed to quantify how the online and face-to-face utilization of healthcare services changed during this time and thus gain insights into the planning of future healthcare resources during the outbreak of infectious diseases.

Design This work is an interrupted time-series study.

Setting We collected monthly online and face-to-face healthcare-service utilization data in 22 public hospitals in Beijing in 2019 and 2020. The face-to-face healthcare-service utilization data were from 22 municipal tertiary public hospitals in Beijing. The online-consultation data were obtained from GoodDoctor (www.haodf.com), which is the largest and longest-running online physician–patient community in China.

Results The monthly average outpatient visits and discharges decreased by 36.33% and 35.75%, respectively, compared with those in 2019 in 22 public hospitals in Beijing. Moreover, the monthly average online consultations increased by 90.06%. A highly significant reduction occurred in the mean outpatients and inpatients, which dropped by 1,755,930 cases (P<0.01) and 5,920,000 cases (P<0.01), respectively. Online consultations rose by 3,650 cases (P<0.05). We identified an immediate and significant drop in healthcare services for four major diseases, i.e., acute myocardial infarction (-174, P<0.1), lung cancer (-2,502, P<0.01), disk disease (-3,756, P<0.01), and Parkinson's disease (-205, P<0.01). Otherwise, online consultations for disk disease (63, P<0.01) and Parkinson's disease (25, P<0.05) significantly increased. More than 1,300 unique physicians provided online-consultation services per month in 2020, which was 35.3% higher than in 2019.

Conclusions Obvious complementary trends in online and face-to-face healthcare services existed during the COVID-19 pandemic. Different changes in healthcare utilization were shown for different diseases. Non-critically ill patients chose online consultation immediately after the

For peer review only

COVID-19 lockdown, but critically ill patients chose hospital healthcare services first. Additionally, the volume of online physician services significantly rose as a result of COVID-19.

Keywords COVID-19, healthcare utilization, online consultation, health-service research

Strengths and limitations of this study

- Based on the Beijing Hospitals Authority and the GoodDoctor data, this work is an interrupted time-series study to quantify how the online and face-to-face utilization of healthcare services changed during the COVID-19 pandemic.
- The methodology in exploring the characteristics and trends of changes in healthcare utilization in the short and long term during the pandemic is noteworthy.
- ITS methodologies such as this have limitations in external environmental factors. Other policies and seasonal confounding factors such as meteorological factors may affect medical visits.

Introduction

In early 2020, the COVID-19 pandemic affected most of the planet and rapidly changed the utilization of healthcare services worldwide [1]. Fear of infection and reduced availability of healthcare services has led to decreased non-COVID healthcare utilization in hospitals [2,3]. The rapid and innovative deployment of telemedicine and online health services has occurred in many countries to relieve the pressure on hospitals and to prevent cross-infection [4,5]. In the United States, emergency room visits dropped by 42% in April, followed by a 26% reduction at the end of May, compared with 2019[6]. Meanwhile, telemedicine visits increased from 102.4 daily to 801.6 daily between March 2nd and April 14th 2020[7].

The impact of the global pandemic on healthcare systems is acute. As the COVID-19 pandemic continues, many studies have reported major changes in the utilization of healthcare services because of measures such as lockdowns and stay-at-home orders. These changes include large reductions in hospital visits, and some selective increases in online health services [8-10]. However, minimal data are available on the relationship between hospital visits and online health services over the same period, especially with quantitative time-trend evidence. Examining the short-term and long-term patterns of changes in hospital volume and online health services during the COVID-19 pandemic is valuable. The extent and effects of substitution

For peer review only

with online healthcare services for different kinds of diseases also require further investigation.

Beijing is a megacity with the most extensive internet accessibility (77.8%) [11] and the most famous hospitals compared with other parts of China. After the COVID-19 pandemic, many patients were unable to access hospitals in Beijing owing to the lockdown policies. In response, online health services and an internet-based health system have been utilized by hospitals in addition to third-party service platforms, such as GoodDoctor (www.haodf.com). Physicians are active in spending their free time on these platforms to provide patients with high-quality online health consultations. Online consultations refer to patients using the Internet to communicate with physicians in real-time through online text, pictures, video, audio, etc.

Figure 1 depicts the epidemic trends and significant changes in the COVID-19 outbreak in Beijing. The first confirmed case of COVID-19 in Beijing occurred on January 20, 2020. Since then, the virus has spread across Beijing. Beijing launched the first-level response mechanism for public health emergencies on January 24, 2020. To respond to public health emergencies promptly, China has launched a tiered response mechanism based on different pandemic levels and hazards. The first-level response mechanism is unified deployment by provincial headquarters according to the decisions of The State Council. The second-level response mechanism refers to deployment by the provincial headquarters. The third-level response mechanism refers to deployment by the headquarters of the prefect-level city. Two peaks occurred on February 2 and March 23 in the early days. Afterwards, the epidemic entered a normalization stage, with no new cases for a long time. On June 11, a 52-year-old man from Xinfadi Market in Beijing was diagnosed with COVID-19. After a 56-day zero-new-case interval, marking the beginning of "the second epidemic," new cases increased sharply on June 13. The number of new cases in a single day reached the highest value of 36 cases. Beijing's response mechanism for public-health emergencies was adjusted from level 3 to level 2 on June 16. This phenomenon led to a fast downward slope with the control of the Chinese government. The epidemic in Beijing has gradually stabilized since then. As of December 31, 987 confirmed cases and 9 deaths had been reported. In the present study, the change in short-term refers to the instantaneous change in medical-service utilization at the beginning of the pandemic from the end of December 2019 to February 2020, whereas long-term analysis refers to a snapshot change within the first year of the pandemic.

[Insert Figure 1 here]

This study was designed to compare and analyze the change trend of online consultation and face-to-face healthcare volume, as well as the change in different disease types during the pandemic. The research illustrates the speed, scale, and reception of online-consultation utilization by patients and physicians, as well as the complementary trends in online and face-to-face health services. The results may help provide insights into the planning of future

For peer review only

healthcare resources during the outbreak of infectious diseases.

Methods

Study Design

We conducted an interrupted time-series analysis to estimate changes in face-to-face and online healthcare utilization before and after the COVID-19 outbreak. Simple pre-post designs perform before–after comparisons by estimating the change from a single pre-intervention time point to a single post-intervention time point. However, these have poor internal validity as they cannot exclude underlying trends as a cause for any change [12]. Conversely, interrupted time series (ITS) use multiple observations in the pre-intervention and post-intervention periods, and thus offer thereby offering a quasi-experimental research design with a potentially high degree of internal validity [13,14]. We treated COVID-19 as the intervention, and 2019 and 2020 as the pre- and post-intervention periods respectively, to analyze the change in face-to-face and online healthcare utilization after the COVID-19 outbreak.

Setting

Monthly hospital-grade healthcare-service data from January 2019 to December 2020 were provided by Beijing Hospitals Authority. Beijing municipal hospitals refer to the public hospitals managed by the Beijing Hospitals Authority of Beijing Municipal Health Commission.

Participants

A total of 22 hospitals were selected in this study, and they covered all tertiary first-class public hospitals managed by the Beijing Hospital Authority. Thirteen were general hospitals and nine were specialized hospitals (obstetrics and gynecology, pediatrics, oncology, stomatology, chest, psychiatry, traditional Chinese medicine, etc.). We collected outpatient and emergency visits, the number of discharges, online consultation volume from all 22 Beijing municipal hospitals, and patients including adults and children.

Patient and Public Involvement

This work was an observational cohort study using publicly available data from the Beijing Hospital Authority and GoodDoctor website. Patients were not involved in our design, conduct,

For peer review only

or reporting.

Data sources

First, outpatient and emergency visits, as well as the number of discharges of 2019 and 2020 from all 22 Beijing municipal hospitals were used as the overall face-to-face healthcare-utilization outcomes. Four diseases including acute myocardial infarction (AMI), lung cancer (LC), disk disease, and Parkinson's disease were selected to analyze changes in healthcare-service utilization for different disease severities. These four diseases belonged to the 20 monitored diseases of the Beijing Municipal Health Commission. We used The International Statistical Classification of Diseases and Related Health Problems, Tenth Revision (ICD-10) to identify the principal diagnoses based on the main diagnosis of the electronic medical-record system of hospitals. Owing to data-sharing restrictions, the monthly number of discharges for these four diseases was collected from July 2019 to December 2020.

Second, online consultation data were obtained from GoodDoctor (www.haodf.com). GoodDoctor is the largest and longest-running online physician–patient communication platform in China [15]. It was founded in 2006. As of October 2021, it has collected information on 860 000 physicians from 9,780 regular hospitals in China, among which more than 73% are physicians in tertiary hospitals [16]. The 22 hospitals are all included in the GoodDoctor platform, covering more than 80% of outpatient doctors of these hospitals. A large number of patients turned to this platform for help after the COVID-19 pandemic outbreak, largely representing the trend in the utilization of online consultations during the pandemic.

The platform provides historical data of online consultations from 2010 to the present. We collected the daily online consultation data of the abovementioned 22 public hospitals in 2019 and 2020 and screened out the online-consultation volume data for AMI, LC, disk disease, and Parkinson's disease based on disease information. Figure 2 shows the content of the online consultation provided by this platform. We used Java language to design a web crawler and capture daily online-consultation information from web pages. Specific extracted fields include the date, website link, title, disease description, disease name, expected treatment, the duration of illness, consultation text of the patient and physician, and the name, title, department, patient voting numbers, online-consultation volume, and satisfaction of online service physicians. The final sample size collected was 70,086 for 2019 and 144,651 for 2020.

[Insert Figure 2 here]

Statistical methods

For peer review only

We fitted an ordinary least squares model by using either regress or Newey (with lag specified) with monthly service volume as the primary outcome of interest. We produced Newey–West standard errors to handle autocorrelation. We treated COVID-19 as the intervention, and 2019 and 2020 as the pre-intervention and post-intervention periods, respectively. The ITSA regression model is expressed as follows:

$$Y_t = \beta_0 + \beta_1 T_t + \beta_2 X_t + \beta_3 X_t T_t + \varepsilon_t$$

where Y_t is the effect variable, including outpatient and emergency visits, discharge volume, and online-consultation volume; T_t is the time since the start of the study, X_t is a dummy variable representing the intervention (pre-intervention period 0, otherwise 1), and X_tT_t is the interaction term; β_0 is the intercept or starting level of the outcome variable; β1 is the slope or trajectory of the outcome variable until the COVID-19 outbreak; β_2 is the change in the level of outcome that occurred in the period immediately following the COVID-19 outbreak (compared with the counterfactual); and β_3 is the difference between the pre-pandemic and pandemic slopes of the outcome. Accordingly, we searched for significant p-values in β_2 to indicate an immediate treatment effect or in β_3 to indicate a treatment effect over time.

Moreover, we determined the impact of COVID-19 on the utilization of healthcare services for different diseases.

We stratified models by disease type restricted to (1) AMI, (2) LC, (3) disk disease, and (4) Parkinson’s disease. They were included in the 20 monitored diseases of Beijing Hospital Authority with clear diagnostic criteria. These four diseases had a certain number of patients online and offline. AMI and LC were considered severe cases, whereas disk disease and Parkinson's disease were considered mild cases in the research. All statistical analyses were conducted using Stata 15.0 and SPSS 20.0.

Bias

Although interrupted time series has high internal validity, external confounding factors cannot be controlled. Since the outbreak of COVID-19 across the entirety of China in 2020, we cannot find an effective control group. A certain confounding bias existed in this study.

Result

1. **Impact of COVID-19 pandemic on the utilization of healthcare services**

Table 1 shows the descriptive statistical indicators of the face-to-face outpatient and

For peer review only

emergency visits, discharges, and online consultations in 2019 and 2020. Compared with 2019, the utilization of face-to-face healthcare services decreased in 2020. The monthly average outpatient and emergency visits, as well as discharges, decreased by 36.33% and 35.75%, respectively. Compared with 2019, the utilization of online consultations increased in 2020. The monthly average online consultations increased by 90.06%.

Table 1 Healthcare-service utilization in 2020 compared with 2019 (ten thousand)

	2019 (before the COVID-19 outbreak)			2020 (after the COVID-19 outbreak)		
	face-to-face outpatient and emergency visits	face-to-face discharges	online consultation	face-to-face outpatient and emergency visits	face-to-face discharges	online consultation
Mean	270.179	8.677	0.634	172.018	5.575	1.205
Std.	25.779	0.918	0.054	54.554	2.160	0.238
Median	274.391	8.956	0.611	179.315	5.996	1.272
Max	311.549	9.559	0.754	243.728	8.292	1.450

Figure 3 shows the percentage changes in healthcare-service utilization in Beijing. Compared with the same period in 2019, we observed lower monthly face-to-face outpatient and emergency visits, as well as discharges in 2020. The percentage change frequency for both was the greatest in March, with 66.97% and 75.17% decrease, respectively. The overall change decreases thereafter. Notably, when affected by “the second epidemic,” face-to-face healthcare services increased slightly again, but utilization in July fell at a higher rate than in June. Online consultations in all months except January were higher than in the same period in 2019. The overall change increased. The greatest decrease was observed in August. Figure 3 shows that in the early days of the pandemic (January to June), the utilization of online and face-to-face healthcare services showed obvious complementary trends. Afterwards, the growth rate of online consultations remained relatively high, whereas the decline rate of face-to-face healthcare services gradually decreased. This finding suggested that the utilization of face-to-face healthcare services is gradually returning to normal levels as COVID-19 fades away, whereas online consultations is maintaining a high growth rate.

[Insert Figure 3 here]

For peer review only

2. Interrupted time-series analysis

Changes in overall online and face-to-face healthcare services

Table 2 shows a highly significant reduction in the mean number of outpatient and emergency visits, as well as discharges, which dropped by 1,755,930 cases (P<0.01) and 59,200 cases (P<0.01), respectively. Before the COVID-19 outbreak, face-to-face healthcare services (including outpatient and emergency visits and discharges) increased (Figure 4). Soon after the COVID-19 outbreak, the slope of this trend line significantly increased, but the trend was not statistically significant. Online consultations also rose by 3,650 cases (P<0.05), followed by a significant increase in the monthly trend of utilization (relative to the pre-pandemic) of 580 cases.

Table 2 Impact of COVID-19 on the utilization of online and face-to-face healthcare services (ten thousand)

	Medical utilization reduction after the COVID-19 outbreak (intercept)	P value	Average monthly medical utilization decline after the COVID-19 outbreak (potential trend)	P value
face-to-face outpatient and emergency visits	-175.593 (27.247*)	<0.01	7.357 (4.454)	>0.1
face-to-face discharges	-5.920 (1.608)	<0.01	0.283 (0.217)	>0.1
online consultation	0.365 (0.143)	<0.05	0.058 (0.019)	<0.01

Standard error in parentheses.

* p<0.1, **p<0.05, ***p<0.01.

[Insert Figure 4 here]

Changes in face-to-face healthcare services by type of disease

Table 3 summarizes the effect of COVID-19 on face-to-face healthcare services for different diseases. The COVID-19 outbreak was associated with a rapid decline in face-to-face healthcare services for different diseases. From the perspective of immediate effect, we identified a significant drop in healthcare services for the four diseases. From the perspective of long-term effects, AMI, LC, and disk disease services showed a nonsignificant stepwise increase (the slope

For peer review only - http://bmjopen.bmj.com/site/about/guidelines.xhtml

of the trend line increased). Simultaneously, the monthly trend of utilization for Parkinson's disease significantly increased (relative to the pre-pandemic period) (Figure 5).

Table 3 Impact of COVID-19 on the utilization of face-to-face healthcare services and online consultation by type of disease

	**Medical utilization reduction after the COVID-19 outbreak (intercept)**		**Average monthly medical utilization decline after the COVID-19 outbreak (potential trend)**	
	face-to-face healthcare services	online consultation	face-to-face healthcare services	online consultation
acute myocardial infarction	-174.405 (84.060) *	-5.998 (3.511)	12.438 (11.540)	1.633 (0.472) ***
lung cancer	-2,502.02 (367.118) ***	-11.832 (19.413)	80.508 (80.480)	14.360 (3.079) ***
disk disease	-375.560 (72.369) ***	63.118 (11.955) ***	29.163 (16.876)	3.402 (1.851) *
Parkinson's disease	-205.229 (47.852) ***	24.625 (10.349) **	12.669 (4.892) **	1.772 (1.335)

Standard error in parentheses.

* p<0.1, **p<0.05, ***p<0.01.

[Insert Figure 5 here]

Changes in online consultations by type of disease

Table 3 summarizes the effect of COVID-19 on online consultations for different diseases. The COVID-19 outbreak was associated with a rapid rise in online consultations for different diseases. From the perspective of immediate effect, we identified a significant increase in healthcare services for disk disease and Parkinson's disease. Conversely, online consultations for AMI and LC showed a small, nonsignificant decrease. From the perspective of long-term effects, online consultations for AMI, LC, and disk disease increased monthly at a rate of 1,633, 14,360, and 3,402, respectively after the COVID-19 outbreak (Figure 6).

[Insert Figure 6 here]

3. **Impact of COVID-19 pandemic on online-service physicians' volume**

As affected by COVID-19, the monthly volume of online services provided by physicians in

For peer review only

2020 was significantly higher than that in 2019, except for January. On average, more than 1,300 unique physicians from 22 hospitals in Beijing provided online-consultation services per month in 2020, which was 35.3% higher than that in 2019. This finding suggested that the supply of online consultations greatly increased after the COVID-19 outbreak (Figure 7).

[Insert Figure 7 here]

Discussion

Based on monthly treatment data from 22 public hospitals in Beijing, obvious complementary trends existed in online and face-to-face healthcare services during the COVID-19 pandemic. We found that public hospitals in Beijing experienced substantial losses in face-to-face healthcare and substantial increases in online-consultation services when the COVID-19 pandemic began in China in 2020. The interrupted time-series models revealed that after the COVID-19 outbreak, the considerable reduction in face-to-face healthcare services was a short-term effect, whereas the increase in online consultation was a long-term one. As only one year of the pandemic was examined in the current study, the long-term effects herein referred to change trends within one year. In other words, this study is a snapshot. Simultaneously, the effects of COVID-19 on healthcare services for different diseases varied. Noncritical-disease patients showed greater reductions in face-to-face service utilization and chose online consultations immediately after the COVID-19 lockdown. However, critical-disease patients chose face-to-face treatment first, and slowly moved online over time. The volume of online services provided by physicians also significantly increased as a result of COVID-19.

Our study is consistent with previous reports on hospital healthcare-service changes during the COVID-19 pandemic [17,18]. A systematic review from the UK analyzed 81 studies and reported that the utilization of healthcare in care facilities sharply decreased by about a third during the pandemic period, with greater reductions among people with less severe illnesses [17]. Additionally, according to a study from one large health system (NYU Langone Health) in the United States, telemedicine visits increased from 102.4 daily to 801.6 daily between March 2 and April 14 2020, with a concomitant decline in in-person visits by over 80%. We also showed that online consultation underwent sustained growth owing to the impact of COVID-19, whereas face-to-face healthcare services showed a short-term decline. An important reason for the decline in face-to-face visits was that rigorous public health measures were implemented in China during the COVID-19 epidemic [19]. Some medical resources responded to the outbreak. The number of outpatient and emergency visits to 22 municipal hospitals in Beijing decreased by 36.33% in 2020. More medical personnel provided epidemic prevention and control services, such as nucleic acid tests and vaccines. Furthermore, potential risk factors in the hospital environment made people anxious and worried. A survey has shown that 20% of pregnant

For peer review only

women fear going to a hospital for consultation, and more than 40% fear going to a hospital for check-ups [20].

A rapid decrease in overall face-to-face discharge admissions was observed for four different diseases across 22 tertiary public hospitals in Beijing after the COVID-19 lockdown. A study performed in Germany revealed a similar reduction in traditional face-to-face healthcare services for different diseases [21]. That study found that hospital admissions for malignant cancer treatments and AMI decreased after the lockdown announcement. However, a difference was that our analysis also showed the impact of the epidemic on diseases with varied severity. We also examined the changes in online consultations for different diseases. We found that different diseases showed different online change trends. Online consultations for critical illnesses (such as AMI, and LC) did not increase immediately after the outbreak but gradually increased in the long-term study. A study has shown that telemedicine can be used to reach patients with severe disease burdens [22]. Conversely, we observed that noncritical clinical situations (such as disk disease and Parkinson's disease) considerably and immediately increased, and this trend has been maintained.

Several reasons may explain the dramatic decrease in face-to-face healthcare services after the COVID-19 pandemic. First, lockdown policy and population mobility control measures were implemented in China to mitigate the spread of the pandemic. For instance, the community implemented closed management, hospital services were limited, and non-emergency services had to be reserved in advance and staggered used in Beijing during the pandemic period. This situation may have reduced the availability and accessibility of face-to-face healthcare services. Second, some patients chose to postpone their treatment to prevent cross-infection in the hospital. Most healthcare resources were relocated to manage the pandemic. This setup may trigger attitudes toward delaying less urgent cases by the patient and the healthcare system. Third, some health demands may have been eliminated. For instance, many patients with self-resolving diseases chose not to avail of face-to-face treatment, such as for colds, diarrhea, etc. [23,24]. The more important point was that some health demands may have been transferred online. Abundant internet diagnosis and treatment services were supplied by medical institutions in Beijing during the pandemic, such as online consultation, instant messaging, synchronous chat, video conferencing, etc. Online consultation can span time and space. The physician and patient do not have to meet each other within a physical space, subsequently decreasing the requirement for face-to-face travel, appointment-related concerns, and the risk of face-to-face infection. Some patients could migrate to online consultation to meet their health needs, which can explain the significantly increased volume of online consultations.

On one hand, our results indicated that mass migration to online consultations occurred shortly after the COVID-19 pandemic. On the other hand, the migration of critically ill patients was slower than that of noncritically ill ones because the latter relied more on facility treatment.

For peer review only

For example, the efficiency of the care pathway at every step matters very much for AMI patients [25]. Chemotherapy, radiation therapy, surgery, and molecular targeted therapy are keystones in the treatment of early and locally advanced LC with good prognosis [26]. These conditions rely on face-to-face operations and inspections, and online consultations cannot provide these services, which is why the online-consultation volume for critical illnesses did not increase immediately after the outbreak. Additionally, we found that the online-consultation volume for three diseases, except for Parkinson's, continuously increased. This phenomenon can be explained by the fact that the COVID-19 pandemic accelerated the popularity of online healthcare, thereby increasing the awareness and acceptance of the public regarding online healthcare.

Our study also found that the pandemic led to a significant increase in online patients and also facilitated the online migration of physicians. The number of physicians offering services online in 2020 was much higher than that over the same period in 2019, except for January. This finding showed that physicians paid attention to providing online healthcare services.

With the COVID-19 pandemic, the supply of face-to-face healthcare services was relatively insufficient, resulting in partial healthcare-service disruption. The COVID-19 pandemic resulted in decreased supply of face-to-face healthcare services, which was part of the healthcare-service disruption. To reduce the public-health impact, we put forward two suggestions: first, we should designate some part of healthcare resources to online provision. Moreover, we should rapidly establish a collaborative model of face-to-face and online healthcare services in public-health emergency situations to meet the demand of patients. Online consultations and telemedicine can alleviate geographic and physician shortages, expand healthcare accessibility, and improve the quality of healthcare provision. Therefore, online healthcare services can serve as an important supplement to traditional healthcare services, optimize health resource allocation, and improve service efficiency, especially for noncritically ill patients.

However, not all diseases are suitable for online consultations. Face-to-face healthcare services are necessary and irreplaceable, such as physical examinations, diagnoses, and treatment of difficult and severe diseases. Different healthcare support measures should be formulated according to the characteristics of different diseases. For critical cases, face-to-face channels ought to be opened for diagnosis and treatment to ensure that patients can receive face-to-face treatment in a timely manner. Studies indicate that 9.1% of patients experienced a delay in LC treatment, and half of AMI patients did not reach out to the hospital at all during the COVID-19 pandemic [25,27]. For critical cases, delaying or stopping treatment can have a significant negative impact on the physical and mental health of critically ill patients. A timely diagnosis and improved treatment strategy will facilitate the health of patients [26]. For mild cases, cooperation among the government, health facilities, and third-party platforms should be enhanced. Online healthcare services such as telemedicine, online consultation, psychological counseling, and

For peer review only

chronic disease revisits ought to be expanded. Expanding the supply of online healthcare resources and helping patients migrate online to receive healthcare services are crucial. Meanwhile, a comparison between the quality of online consultation and face-to-face care is worth discussing. An in-depth review shows that online consultation is a useful, high-quality, and safe option for the vast majority of pediatric neurology patients [28]. A study has shown that no significant difference exists between the satisfaction of online counseling and face-to-face healthcare [29]. However, studies have also shown that people still worry about the reliability of online healthcare [30].

Finally, our work confirmed that the increase in online consultations was a persistent phenomenon during the research period. A new normal deployment of online healthcare services occurred in the pandemic and policy-enabling environment. The development of online consultation should be encouraged because of the advantages of online consultation across time and space. New technologies such as 5G technology, sensing techniques, and artificial intelligence are bound to be fully exploited for online consultation. Innovating the long-term synergistic modalities of online and face-to-face healthcare services is important to promote the connection among high-quality medical resources at all levels.

This study had some limitations. First, we studied the complementary trend of online and face-to-face health service utilization. Whether the change in service delivery caused an impact on the quality or the equivalency of care delivery requires further study. Second, to observe the changes in healthcare-service utilization, the behavior of patients with diseases of varied severity, i.e., AMI, LC, disk disease, and Parkinson’s disease, was selected in this paper. Further studies are needed to determine whether this phenomenon can be extended to other diseases. The heterogeneity of the data across the hospitals was also not explored. Third, the usage of GoodDoctor cannot cover all the online-consultation behavior. Although GoodDoctor is the largest online consultant platform in China, some other online-consultant platforms are available in the industry. We choose the GoodDoctor platform by considering that the 22 surveyed hospitals all had a certain number of doctors in the platform, which was representative to a certain extent. Finally, this study was only a snapshot capturing the first year of the pandemic response. Comprehensive research over a longer period is required.

Conclusion

Face-to-face healthcare services dropped precipitously and online consultations rose sharply in 22 tertiary public hospitals in Beijing during the COVID-19 pandemic. However, face-to-face healthcare services showed a relatively fast recovery, and online consultations continued to grow. The change trend in online-consultation volume varied by disease, which was characterized by a nonsignificant increase in the short term and a significant increase in the long

For peer review only

term regarding critical cases after the outbreak, as well as a significant increase in noncritical cases in the long short term. The availability of online consultations should be encouraged during pandemics. Particular attention should be given to severely ill patients who need adequate and prompt face-to-face hospital care.

Author affiliations

1 The Seventh Affiliated Hospital, Sun Yat-sen University, Guangdong, China

2 School of Public Health, Capital Medical University, Beijing, China

Contributors The research idea originated with CM, but all authors designed the study. SZ directed the data analyses. CM and SZ co-wrote this manuscript.

Funding This study has been funded by the Natural Science Foundation of Beijing Municipality (grant number: 9222993).

Competing interests We have read and understood the BMJ policy on the declaration of interests. All authors report no competing interests.

Patient and public involvement statement Patients and the public were not involved in the design or conduct of this study. The study used publicly available data from GoodDoctor, which has signed privacy policies with users.

Patient consent for publication Not applicable.

Ethics approval It was approved by the ethics committee of Capital Medical University (approval No. Z2020SY126).

For peer review only

Provenance and peer review Not commissioned; externally peer-reviewed.

Data availability statement Data are available on reasonable request.

Abbreviations

AIM: Acute Myocardial Infarction

LC: Lung Cancer

References

1 Moynihan R, Sanders S, Michaleff Z A, et al. Impact of COVID-19 pandemic on utilisation of healthcare services: a systematic review[J]. BMJ Open, 2021, 11(3): e045343.

2 Xu S, Glenn S, Sy L,, et al. Impact of the COVID-19 Pandemic on Health Care Utilization in a Large Integrated Health Care System: Retrospective Cohort Study. J Med Internet Res. 2021 Apr 29;23(4): e26558.

3 Hartnett KP, Kite-Powell A, DeVies J, et al. National Syndromic Surveillance Program Community of Practice. Impact of the COVID-19 Pandemic on Emergency Department Visits - United States, January 1, 2019-May 30, 2020. MMWR Morb Mortal Wkly Rep. 2020 Jun 12;69(23):699-704.

4 Lukas H, Xu C, Yu Y, et al. Emerging Telemedicine Tools for Remote COVID-19 Diagnosis, Monitoring, and Management. ACS Nano. 2020 Dec 22;14(12):16180-16193.

5 Bokolo Anthony Jnr. Use of Telemedicine and Virtual Care for Remote Treatment in Response to COVID-19 Pandemic. J Med Syst. 2020 Jun 15;44(7):132.

6 Garcia S, Albaghdadi M S, Meraj P M, et al. Reduction in ST-Segment Elevation Cardiac Catheterization Laboratory Activations in the United States During COVID-19 Pandemic[J]. Journal of the American College of Cardiology, 2020.

7 Mann DM, Chen J, Chunara R, et al. COVID-19 transforms health care through telemedicine: Evidence from the field. J Am Med Inform Assoc. 2020 Jul 1;27(7):1132-1135.

8 Chen Y, Cai M, Li Z, et al. Impacts of the COVID-19 Pandemic on Public Hospitals of Different Levels: Six-Month Evidence from Shanghai, China. Risk Manag Healthc Policy. 2021;14: 3635-3651.

For peer review only

9 Li G, Fan G, Chen Y, et al. What patients "see" doctors in online fever clinics during COVID-19 in Wuhan? J Am Med Inform Assoc. 2020 Jul 1;27(7):1067-1071.

10 Shimels T. The Trend of Health Service Utilization and Challenges Faced During the COVID-19 Pandemic at Primary Units in Addis Ababa: A Mixed-Methods Study. Health Serv Res Manag Epidemiol. 2021 Jul 8;8:23333928211031119.

11 China Internet Network Information center. Beijing, China: CNNIC, 2021. Annual Report 2021.

12 James, Lopez, Bernal, et al. The use of controls in interrupted time series studies of public health interventions.[J]. International Journal of Epidemiology, 2018.

13 Shadish W, Cook T, Campbell D. Experimental and Quasi-Experimental Designs For Generalized Causal Inference. Houghton Mifflin, 2002.

14 Miller CJ, Smith SN, Pugatch M. Experimental and quasi-experimental designs in implementation research. Psychiatry Res. 2020 Jan;283:112452.

15 Liu X, Chen M, Li J, et al. How to Manage Diversity and Enhance Team Performance: Evidence from Online Doctor Teams in China. Int J Environ Res Public Health. 2019 Dec 19;17(1):48.

16 The GoodDoctor profile, The GoodDoctor. 2022. http://www.haodf.com/info/aboutus.php

17 Moynihan R, Sanders S, Michaleff ZA, et al. Impact of COVID-19 pandemic on utilisation of healthcare services: a systematic review. BMJ Open. 2021 Mar 16;11(3): e045343.

18 Mann DM, Chen J, Chunara R, et al. COVID-19 transforms health care through telemedicine: Evidence from the field. J Am Med Inform Assoc. 2020 Jul 1;27(7):1132-1135.

19 Yang ZM, Wu MY, Lu JM, et al. Effect of COVID-19 on hospital visits in Ningbo, China: an interrupted time-series analysis. Int J Qual Health Care. 2021 May 7;33(2): mzab078.

20 Wu H, Sun W, Huang X, et al. Online Antenatal Care During the COVID-19 Pandemic: Opportunities and Challenges. J Med Internet Res. 2020 Jul 22;22(7): e19916

21 Kapsner LA, Kampf MO, Seuchter SA, et al. Reduced Rate of Inpatient Hospital Admissions in 14 German University Hospitals During the COVID-19 Lockdown. Front Public Health. 2021 Jan 13;8: 594117.

22 Shehan JN, Agarwal P, Danis DO 3rd, et al. Effects of COVID-19 on telemedicine practice patterns in outpatient otolaryngology. Am J Otolaryngol. 2021 Nov-Dec;42(6):103044.

23 Giannouchos TV, Biskupiak J, Moss MJ, et al. Trends in outpatient emergency department visits during the COVID-19 pandemic at a large, urban, academic hospital system. Am J Emerg Med. 2021 Feb;40:20-26.

For peer review only

24 Meng H, Ji LN, Huang J, et al. Analysis of the changes and characteristics of pediatric outpatient visits in a general hospital in Beijing before and after the COVID-19 pandemic. Beijing Da Xue Xue Bao Yi Xue Ban. 2021 Oct 18;53(5):952-956. Chinese.

25 German University Hospitals During the COVID-19 Lockdown. Front Public Health. 2021 Jan 13;8: 594117.

26 De Rosa S, Spaccarotella C, Basso C, et al. Reduction of hospitalizations for myocardial infarction in Italy in the COVID-19 era. Eur Heart J. 2020 Jun 7;41(22):2083-2088.

27 Sha Z, Chang K, Mi J, Liang Z, et al. The impact of the COVID-19 pandemic on lung cancer patients. Ann Palliat Med. 2020 Sep;9(5):3373-3378.

28 Joshi CN, Yang ML, Eschbach K, et al. Quality and Safety Analysis of 2,999 Telemedicine Encounters During the COVID-19 Pandemic. Neurol Clin Pract. 2021 Apr;11(2): e73-e82.

29 Fieux M, Duret S, Bawazeer N, et al. Telemedicine for ENT: Effect on quality of care during Covid-19 pandemic. Eur Ann Otorhinolaryngol Head Neck Dis. 2020 Sep;137(4):257-261.

30 Wu H, Sun W, Huang X, et al. Online Antenatal Care During the COVID-19 Pandemic: Opportunities and Challenges. J Med Internet Res. 2020 Jul 22;22(7): e19916.

Figure legends

Figure 1 COVID-19 epidemic trend in Beijing

Figure 2 Information on online consultation provided by this platform

Figure 3 Percentage change in healthcare-service utilization in 2020 compared with that in 2019

Figure 4 Effect of COVID-19 intervention on face-to-face and online healthcare utilization. Scatter plots represent monthly medical utilization. The black line of best fit represents medical utilization across estimated by post-regression. The vertical black line is the intervention point. (a) Face-to-face outpatient and emergency visits, (b) face-to-face discharges, and (c) online consultation.

For peer review only

Figure 5 Effect of COVID-19 intervention on face-to-face healthcare utilization by type of disease. Scatter plots represent medical utilization monthly. The black line of best fit represents medical utilization across estimated by post-regression. The vertical black line is the intervention point. (a)AMI, (b) LC, (c) disk disease, and (d) Parkinson's disease.

Figure 6 Effect of COVID-19 intervention on online-consultation utilization by disease type. Scatter plots represent monthly medical utilization. The black line of best fit represents medical utilization across estimated by post-regression. The vertical black line is the intervention point. (a)AMI, (b) LC, (c) disk disease, and (d) Parkinson's disease.

Figure 7 Changes in online physician volume in 2020 compared with that in 2019. The dotted line is the mean.

For peer review only

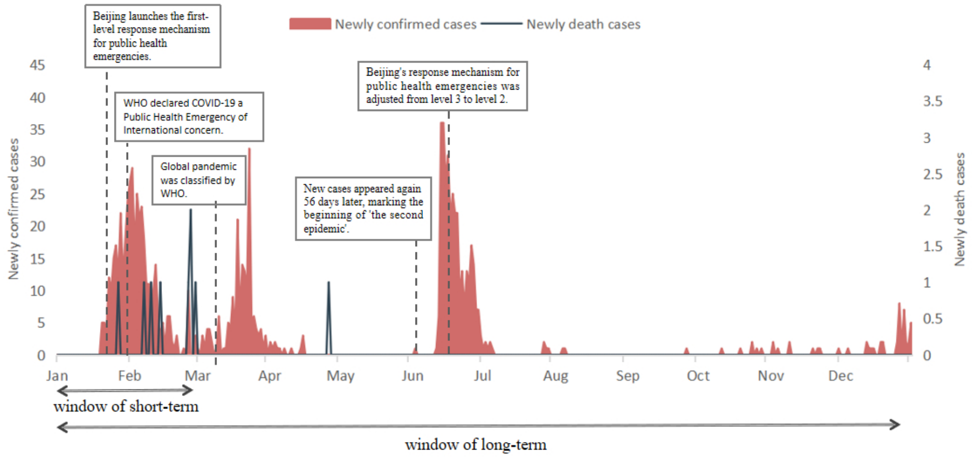

Figure 1 COVID-19 epidemic trend in Beijing

478x244mm (57 x 57 DPI)

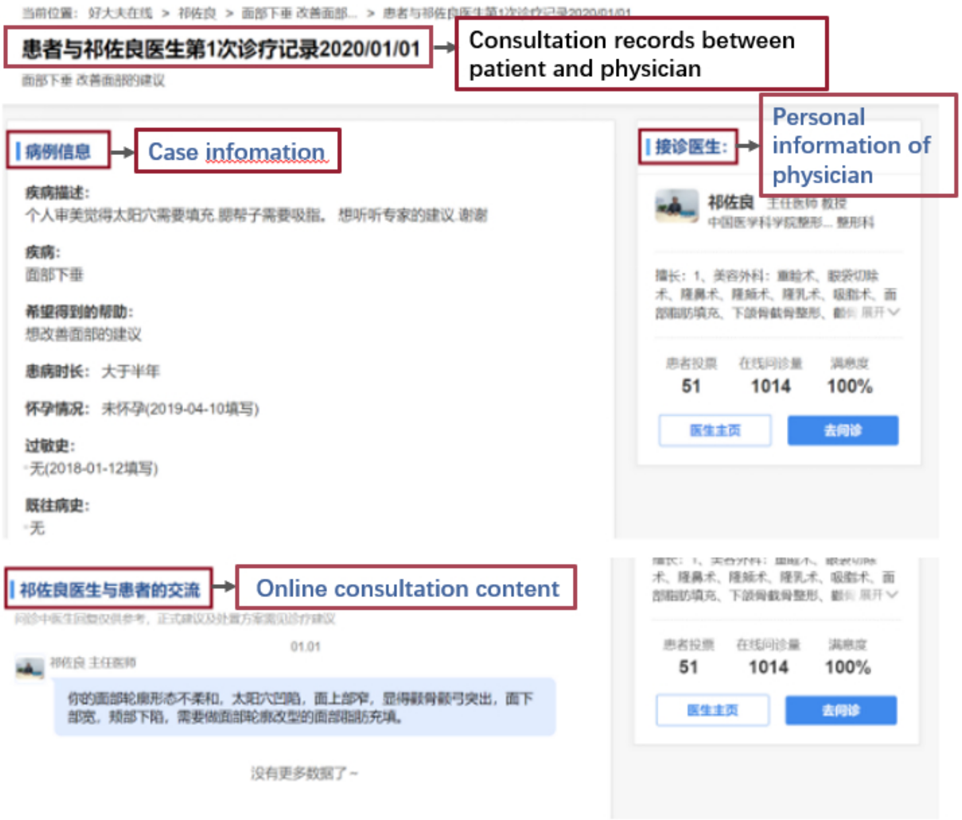

Figure 2 The information of the online consultation provided by GoodDoctor platform

331x283mm (57 x 57 DPI)

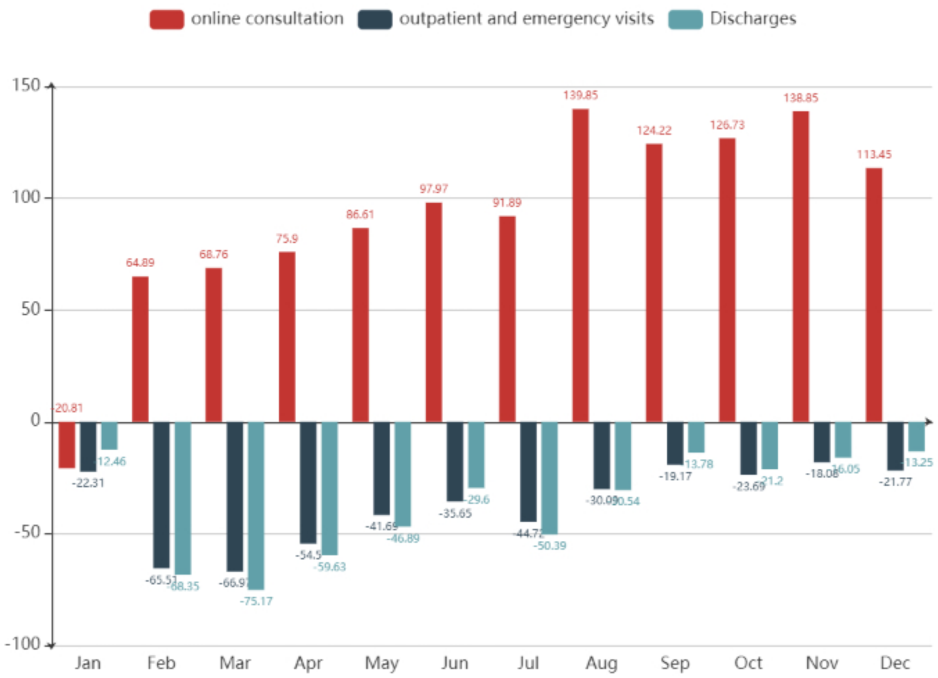

Figure 3 Percentage change in healthcare service utilization in 2020 compared with 2019 (%)

254x175mm (72 x 72 DPI)

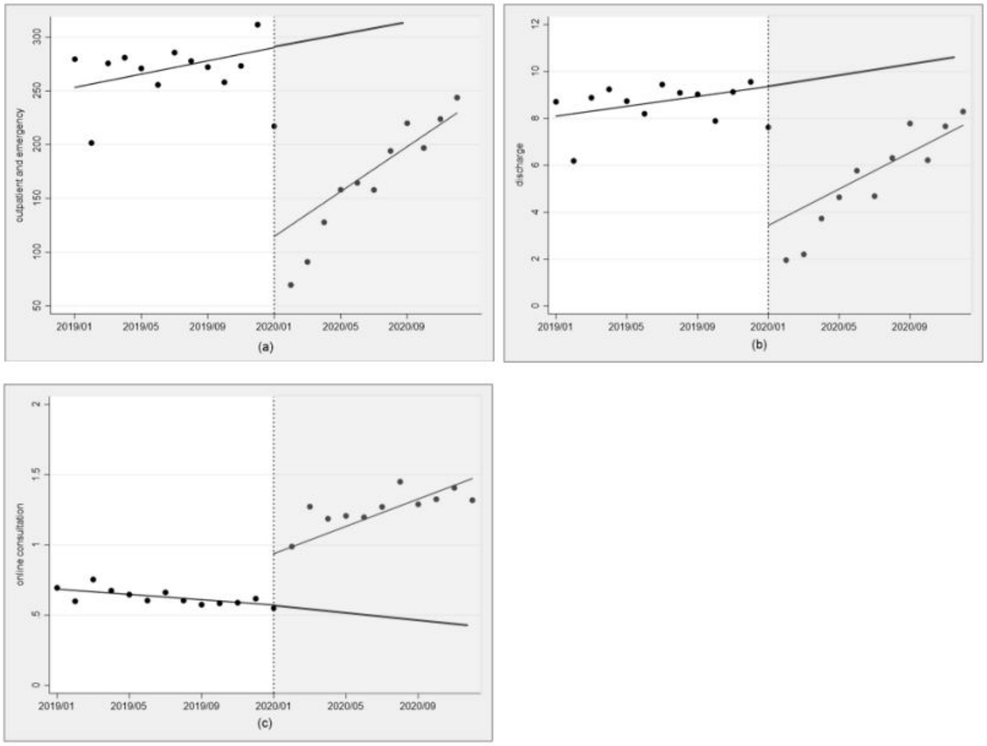

Figure 4 The effect of COVID-19 intervention on face-to-face and online healthcare utilization. Scatter plots represent monthly medical utilization. The black line of best fit represents medical utilization across estimated by post-regression. The vertical black line is the intervention point. (a) face-to-face outpatient and emergency visits; (b) face-to-face discharges; (c) online consultation.

143x108mm (144 x 144 DPI)

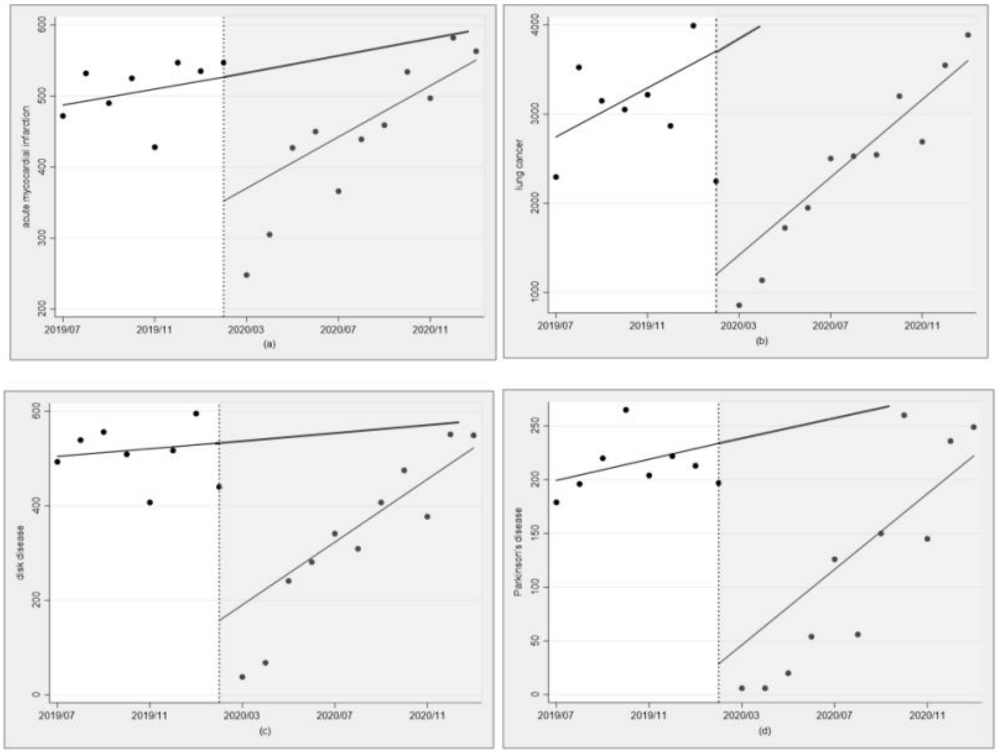

Figure 5 The effect of COVID-19 intervention on face-to-face healthcare utilization by type of disease. Scatter plots represent medical utilization monthly. The black line of best fit represents medical utilization across estimated by post-regression. The vertical black line is the intervention point. (a) AMI; (b)LC; (c) disk disease; (d) Parkinson's disease.

142x108mm (144 x 144 DPI)

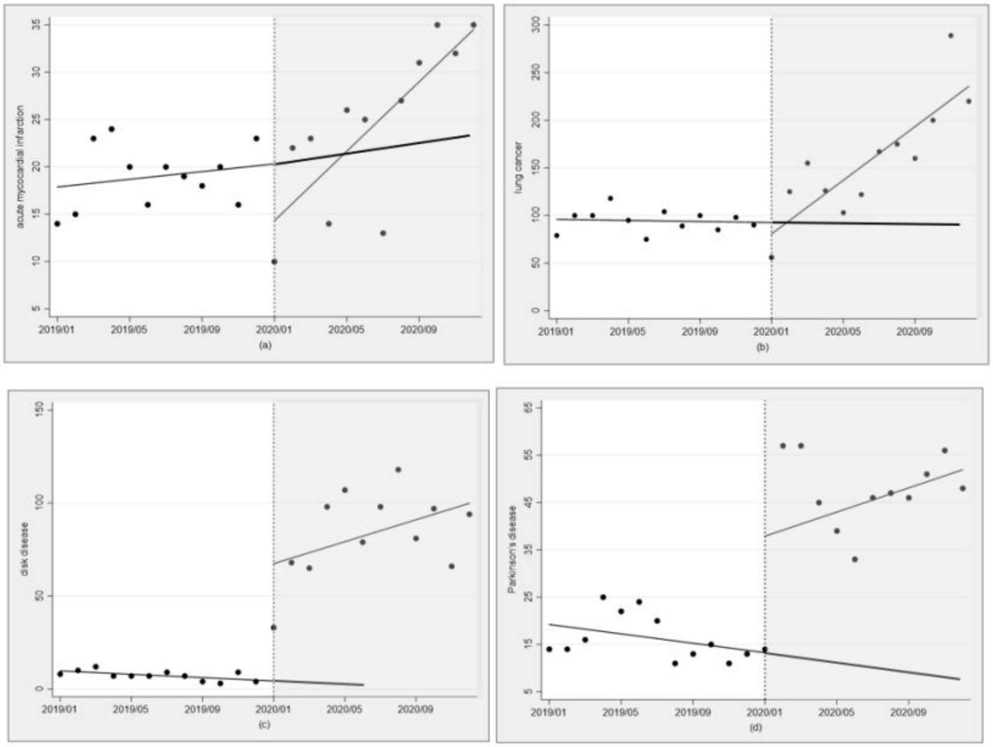

Figure 6 The effect of COVID-19 intervention on online consultations utilization by type of disease. Scatter plots represent medical utilization monthly. The black line of best fit represents medical utilization across estimated by post-regression. The vertical black line is the intervention point. (a)AMI; (b) LC; (c) disk disease; (d) Parkinson's disease.

144x108mm (144 x 144 DPI)

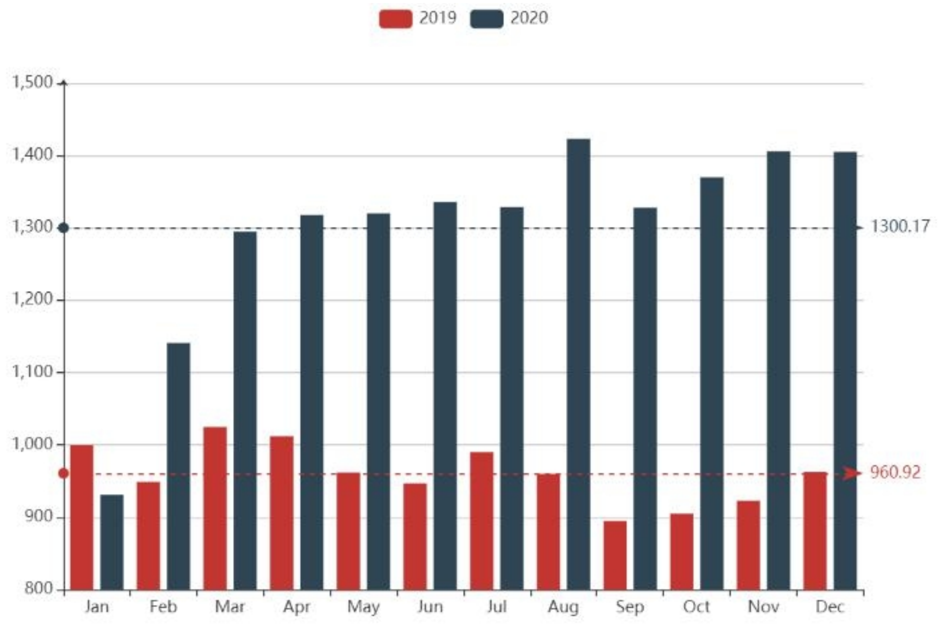

Figure 7 Changes in online physician volume in 2020 compared with 2019 (%).
The dotted line is the mean.

131x88mm (144 x 144 DPI)

STROBE Statement—checklist of items that should be included in reports of observational studies

	Item No.	Recommendation	Page No.	Relevant text from manuscript
**Title and abstract**	1	(*a*) Indicate the study's design with a commonly used term in the title or the abstract	Page 1 line 1-2	COVID-19, interrupted time-series study
		(*b*) Provide in the abstract an informative and balanced summary of what was done and what was found	Page 1 line 9-32, Page 2 line 1-2	We report the main research contents, results and conclusions in the abstract. We found that the online and offline trends were complementary during the pandemic. Obvious complementary trends in online and face-to-face healthcare services existed during the COVID-19 pandemic through the time series analysis. And different changes in healthcare utilization were shown for different diseases.
**Introduction**				
Background/rationale	2	Explain the scientific background and rationale for the investigation being reported	Page 2 line 17-24, Page 3 line 2-30	In the context of the COVID-19 pandemic, fear of infection and reduced availability of healthcare services has led to decreased non-COVID healthcare utilization in hospitals. And online health services have occurred in many countries to relieve the pressure on hospitals and to prevent cross-infection. We described the pandemic situation in Beijing.
Objectives	3	State specific objectives, including any prespecified hypotheses	Page 3 line 17-24	This study was designed to compare and analyze the changing trend of online consultation and face-to-face healthcare volume, as well as the change in different disease types during the pandemic. The research illustrates the speed, scale, and reception of online-consultation utilization by patients and physicians, as

For peer review only - http://bmjopen.bmj.com/site/about/guidelines.xhtml

For peer review only

				well as the complementary trends in online and face-to-face health services.
**Methods**				
Study design	4	Present key elements of study design early in the paper	Page 4 line 5-14	We conducted an interrupted time-series analysis. We treated COVID-19 as the intervention, and 2019 and 2020 as the pre- and post-intervention periods respectively, to analyze the change in face-to-face and online healthcare utilization after the COVID-19 outbreak.
Setting	5	Describe the setting, locations, and relevant dates, including periods of recruitment, exposure, follow-up, and data collection	Page 4 line 17-19	Monthly hospital-grade healthcare-service data from January 2019 to December 2020 were provided by Beijing Hospitals Authority. Beijing municipal hospitals refer to the public hospitals managed by the Beijing Hospitals Authority of Beijing Municipal Health Commission.
Participants	6	(*a*) *Cohort study*—Give the eligibility criteria, and the sources and methods of selection of participants. Describe methods of follow-up<br><br>*Case-control study*—Give the eligibility criteria, and the sources and methods of case ascertainment and control selection. Give the rationale for the choice of cases and controls<br><br>*Cross-sectional study*—Give the eligibility criteria, and the sources and methods of selection of participants	Page 4 line 22-27	A total of 22 hospitals were selected in this study, and they covered all tertiary first-class public hospitals managed by the Beijing Hospital Authority. Thirteen were general hospitals and nine were specialized hospitals (obstetrics and gynecology, pediatrics, oncology, stomatology, chest, psychiatry, traditional Chinese medicine, etc.).
		(*b*) *Cohort study*—For matched studies, give matching criteria and number of exposed and unexposed		N/A

For peer review only - http://bmjopen.bmj.com/site/about/guidelines.xhtml

For peer review only

		Case-control study—For matched studies, give matching criteria and the number of controls per case		
Variables	7	Clearly define all outcomes, exposures, predictors, potential confounders, and effect modifiers. Give diagnostic criteria, if applicable	Page 6 line 1-5	We fitted an ordinary least squares model by using either regress or Newey (with lag specified) with monthly service volume as the primary outcome of interest. We produced Newey–West standard errors to handle autocorrelation. We treated COVID-19 as the intervention, and 2019 and 2020 as the pre-intervention and post-intervention periods, respectively.
Data sources/ measurement	8*	For each variable of interest, give sources of data and details of methods of assessment (measurement). Describe comparability of assessment methods if there is more than one group	Page 5 line 4-31	Outpatient and emergency visits, as well as the number of discharges in 2019 and 2020 from all 22 Beijing municipal hospitals, were used as the overall face-to-face healthcare utilization outcomes. And the monthly number of discharges for AMI, LC, disk disease, and Parkinson's disease four diseases were collected from July 2019 to December 2020. Online consultation data were obtained from GoodDoctor
Bias	9	Describe any efforts to address potential sources of bias	Page 6 line 26-28	Although interrupted time series has high internal validity, external confounding factors cannot be controlled. Since the outbreak of COVID-19 across the entirety of China in 2020, we cannot find an effective control group. A certain confounding bias existed in this study.
Study size	10	Explain how the study size was arrived at	Page 4 line 22-27	A total of 22 hospitals were selected in this study, and they covered all tertiary first-class public hospitals managed by the Beijing Hospital Authority. Thirteen were general hospitals and nine were specialized hospitals (obstetrics and gynecology, pediatrics, oncology, stomatology, chest, psychiatry, traditional Chinese medicine, etc.).We collected outpatient and emergency visits, the number of discharges, online consultation volume from

For peer review only - http://bmjopen.bmj.com/site/about/guidelines.xhtml

	all 22 Beijing municipal hospitals, and patients including adults and children.

Continued on next page

For peer review only

For peer review only - http://bmjopen.bmj.com/site/about/guidelines.xhtml

Quantitative variables	11	Explain how quantitative variables were handled in the analyses. If applicable, describe which groupings were chosen and why	Page 6 line 6-15	Y_t is the effect variable, including outpatient and emergency visits, discharge volume, and online-consultation volume; Tt is the time since the start of the study, X_t is a dummy variable representing the intervention (pre-intervention period 0, otherwise 1), and X_tT_t is the interaction term; β_0 is the intercept or starting level of the outcome variable; β_1 is the slope or trajectory of the outcome variable until the COVID-19 outbreak; β_2 is the change in the level of outcome that occurred in the period immediately following the COVID-19 outbreak (compared with the counterfactual), and β3 is the difference between the pre-pandemic and pandemic slopes of the outcome. All statistical analyses were conducted using Stata 15.0 and SPSS 20.0.
Statistical methods	12	(*a*) Describe all statistical methods, including those used to control for confounding	Page 4 line 9-12	Interrupted time series (ITS) use multiple observations in the pre-intervention and post-intervention periods, and thus offer thereby offering a quasi-experimental research design with a potentially high degree of internal validity
		(*b*) Describe any methods used to examine subgroups and interactions	Page 4 line 8-10	Tt is the time since the start of the study, X_t is a dummy variable representing the intervention (pre-intervention period 0, otherwise 1), and X_tT_t is the interaction term.
		(*c*) Explain how missing data were addressed	N/A	
		(*d*) *Cohort study*—If applicable, explain how loss to follow-up was addressed<br>*Case-control study*—If applicable, explain how matching of cases and controls was addressed<br>*Cross-sectional study*—If applicable, describe analytical methods taking account of sampling strategy	N/A	

For peer review only - http://bmjopen.bmj.com/site/about/guidelines.xhtml

For peer review only

		(*e*) Describe any sensitivity analyses	N/A	
**Results**				
Participants	13*	(a) Report numbers of individuals at each stage of study—eg numbers potentially eligible, examined for eligibility, confirmed eligible, included in the study, completing follow-up, and analysed	N/A	There was no follow-up in our study .
		(b) Give reasons for non-participation at each stage	N/A	
		(c) Consider use of a flow diagram	N/A	
Descriptive data	14*	(a) Give characteristics of study participants (eg demographic, clinical, social) and information on exposures and potential confounders	Page 6 line 32-Page 7 line 5	We described healthcare service utilization in 2020 compared with 2019.
		(b) Indicate number of participants with missing data for each variable of interest	N/A	
		(c) *Cohort study*—Summarise follow-up time (eg, average and total amount)	N/A	
Outcome data	15*	*Cohort study*—Report numbers of outcome events or summary measures over time	Page 7 line 8-22	We described the percentage changes in healthcare-service utilization in Beijing.
		Case-control study—Report numbers in each exposure category, or summary measures of exposure	N/A	
		Cross-sectional study—Report numbers of outcome events or summary measures	N/A	
Main results	16	(*a*) Give unadjusted estimates and, if applicable, confounder-adjusted estimates and their precision (eg, 95% confidence	Page 8 line 2-Page 9 line 16	We described the results of the discontinuous time series

For peer review only - http://bmjopen.bmj.com/site/about/guidelines.xhtml

	interval). Make clear which confounders were adjusted for and why they were included	
	(*b*) Report category boundaries when continuous variables were categorized	N/A
	(*c*) If relevant, consider translating estimates of relative risk into absolute risk for a meaningful time period	N/A

Continued on next page

For peer review only

For peer review only - http://bmjopen.bmj.com/site/about/guidelines.xhtml

Other analyses	17	Report other analyses done—eg analyses of subgroups and interactions, and sensitivity analyses	Page 9 line 19-Page 10 line 4	We described the changes in online physician volume in 2020 compared with 2019.
**Discussion**				
Key results	18	Summarise key results with reference to study objectives	Page 10 line 7-20	Obvious complementary trends existed in online and face-to-face healthcare services during the COVID-19 pandemic. We found that public hospitals in Beijing experienced substantial losses in face-to-face healthcare and substantial increases in online-consultation services when the COVID-19 pandemic began in China in 2020. The interrupted time-series models revealed that after the COVID-19 outbreak, the considerable reduction in face-to-face healthcare services was a short-term effect, whereas the increase in online consultation was a long-term one.
Limitations	19	Discuss limitations of the study, taking into account sources of potential bias or imprecision. Discuss both direction and magnitude of any potential bias	Page 13 line 15-Page 13 line 27	This study had some limitations. First, we studied the complementary trend of online and face-to-face health service utilization. Whether the change in service delivery caused an impact on the quality or the equivalency of care delivery requires further study. Second, to observe the changes in healthcare-service utilization, the behaviour of patients with diseases of varied severity, i.e., AMI, LC, disk disease, and Parkinson's disease, was selected in this paper. Further studies are needed to determine whether this phenomenon can be extended to other diseases. The heterogeneity of the data across the hospitals was also not explored. Third, the usage of GoodDoctor cannot cover all the online-consultation behaviour. Finally, this study was only a snapshot capturing the first year of the pandemic response. Comprehensive research over a longer period is required.

For peer review only - http://bmjopen.bmj.com/site/about/guidelines.xhtml

For peer review only

Interpretation	20	Give a cautious overall interpretation of results considering objectives, limitations, multiplicity of analyses, results from similar studies, and other relevant evidence	Page 10 line 21-Page 13 line 14	The behaviour of patients with diseases of varied severity, i.e., AMI, LC, disk disease, and Parkinson's disease, was selected in this paper. Further studies are needed to determine whether this phenomenon can be extended to other diseases.
Generalisability	21	Discuss the generalisability (external validity) of the study results	Page 13 line 17-20	Further studies are needed to determine whether this phenomenon can be extended to other diseases.
**Other information**				
Funding	22	Give the source of funding and the role of the funders for the present study and, if applicable, for the original study on which the present article is based	Page 14 line 11-12	This study has been funded by the Natural Science Foundation of Beijing Municipality (grant number: 9222993).

*Give information separately for cases and controls in case-control studies and, if applicable, for exposed and unexposed groups in cohort and cross-sectional studies.

Note: An Explanation and Elaboration article discusses each checklist item and gives methodological background and published examples of transparent reporting. The STROBE checklist is best used in conjunction with this article (freely available on the Web sites of PLoS Medicine at http://www.plosmedicine.org/, Annals of Internal Medicine at http://www.annals.org/, and Epidemiology at http://www.epidem.com/). Information on the STROBE Initiative is available at www.strobe-statement.org.

For peer review only

BMJ Open

How has the COVID-19 pandemic affected the utilization of online consultation and face-to-face medical treatment? An interrupted time-series study in Beijing, China.

Journal:	*BMJ Open*
Manuscript ID	bmjopen-2022-062272.R2
Article Type:	Original research
Date Submitted by the Author:	29-Dec-2022
Complete List of Authors:	Zhang, Shan; The Seventh Affiliated Hospital Sun Yat-sen University<br>Ma, Chengyu; Capital Medical University, School of Public Health
**Primary Subject Heading**:	Public health
Secondary Subject Heading:	Health policy, Medical management, Public health
Keywords:	COVID-19, PUBLIC HEALTH, SOCIAL MEDICINE

SCHOLARONE™ Manuscripts

BMJ

I, the Submitting Author has the right to grant and does grant on behalf of all authors of the Work (as defined in the below author licence), an exclusive licence and/or a non-exclusive licence for contributions from authors who are: i) UK Crown employees; ii) where BMJ has agreed a CC-BY licence shall apply, and/or iii) in accordance with the terms applicable for US Federal Government officers or employees acting as part of their official duties; on a worldwide, perpetual, irrevocable, royalty-free basis to BMJ Publishing Group Ltd ("BMJ") its licensees and where the relevant Journal is co-owned by BMJ to the co-owners of the Journal, to publish the Work in this journal and any other BMJ products and to exploit all rights, as set out in our licence.

The Submitting Author accepts and understands that any supply made under these terms is made by BMJ to the Submitting Author unless you are acting as an employee on behalf of your employer or a postgraduate student of an affiliated institution which is paying any applicable article publishing charge ("APC") for Open Access articles. Where the Submitting Author wishes to make the Work available on an Open Access basis (and intends to pay the relevant APC), the terms of reuse of such Open Access shall be governed by a Creative Commons licence – details of these licences and which Creative Commons licence will apply to this Work are set out in our licence referred to above.

Other than as permitted in any relevant BMJ Author's Self Archiving Policies, I confirm this Work has not been accepted for publication elsewhere, is not being considered for publication elsewhere and does not duplicate material already published. I confirm all authors consent to publication of this Work and authorise the granting of this licence.

For peer review only

How has the COVID-19 pandemic affected the utilization of online consultation and face-to-face medical treatment? An interrupted time-series study in Beijing, China.

[1] The Seventh Affiliated Hospital, Sun Yat-sen University, Guangdong, China

[2] School of Public Health, Capital Medical University, Beijing, China

First Author: Shan Zhang, Master's Degree, zsunll2017@163.com

Corresponding Author: Chengyu Ma, Associate Professor, 10132415@qq.com

For peer review only

Abstract

Objective The COVID-19 pandemic has had a major impact on healthcare utilization. This study aimed to quantify how the online and face-to-face utilization of healthcare services changed during this time and thus gain insights into the planning of future healthcare resources during the outbreak of infectious diseases.

Design This work is an interrupted time-series study.

Setting We collected monthly online and face-to-face healthcare-service utilization data in 22 public hospitals in Beijing in 2019 and 2020. The face-to-face healthcare-service utilization data were from 22 municipal tertiary public hospitals in Beijing. The online-consultation data were obtained from GoodDoctor (www.haodf.com), which is the largest and longest-running online physician–patient community in China.

Participants A total of 22 hospitals were selected in this study, and they covered all tertiary first-class public hospitals managed by the Beijing Hospital Authority. Thirteen were general hospitals and nine were specialized hospitals.

Interventions The outbreak of COVID-19 was used as an intervention in this study, to explore the short-term and long-term changes in the amount of treatment. The change in short-term refers to the instantaneous change in medical-service utilization at the beginning of the pandemic from the end of December 2019 to February 2020, whereas long-term analysis refers to a snapshot change within the first year of the pandemic.

Primary outcome measures The main indicators of this study are outpatient and emergency visits, discharge volume, and online consultation volume.

Results The monthly average outpatient visits and discharges decreased by 36.33% and 35.75%, respectively, compared with those in 2019 in 22 public hospitals in Beijing. Moreover, the monthly average online consultations increased by 90.06%. A highly significant reduction

occurred in the mean outpatients and inpatients, which dropped by 1,755,930 cases (P<0.01) and 5,920,000 cases (P<0.01), respectively. Online consultations rose by 3,650 cases (P<0.05). We identified an immediate and significant drop in healthcare services for four major diseases, i.e., acute myocardial infarction(AMI) (-174, P<0.1), lung cancer(LC) (-2,502, P<0.01), disk disease (-3,756, P<0.01), and Parkinson's disease (-205, P<0.01). Otherwise, online consultations for disk disease (63, P<0.01) and Parkinson's disease (25, P<0.05) significantly increased. More than 1,300 unique physicians provided online-consultation services per month in 2020, which was 35.3% higher than in 2019.

Conclusions Obvious complementary trends in online and face-to-face healthcare services existed during the COVID-19 pandemic. Different changes in healthcare utilization were shown for different diseases. Non-critically ill patients chose online consultation immediately after the COVID-19 lockdown, but critically ill patients chose hospital healthcare services first. Additionally, the volume of online physician services significantly rose as a result of COVID-19.

Keywords COVID-19, healthcare utilization, online consultation, health-service research

Strengths and limitations of this study

- Based on the Beijing Hospitals Authority and the GoodDoctor data, this work is an interrupted time-series study to quantify how the online and face-to-face utilization of healthcare services changed during the COVID-19 pandemic.
- The methodology in exploring the characteristics and trends of changes in healthcare utilization in the short and long term during the pandemic is noteworthy.
- ITS methodologies such as this have limitations in external environmental factors. Other policies and seasonal confounding factors such as meteorological factors may affect medical visits.

Introduction

In early 2020, the COVID-19 pandemic affected most of the planet and rapidly changed the utilization of healthcare services worldwide [1]. Fear of infection and reduced availability of healthcare services has led to decreased non-COVID healthcare utilization in hospitals [2,3]. The rapid and innovative deployment of telemedicine and online health services has occurred in many countries to relieve the pressure on hospitals and to prevent cross-infection [4,5]. In the

For peer review only

United States, emergency room visits dropped by 42% in April, followed by a 26% reduction at the end of May, compared with 2019[6]. Meanwhile, telemedicine visits increased from 102.4 daily to 801.6 daily between March 2nd and April 14th 2020[7].

The impact of the global pandemic on healthcare systems is acute. As the COVID-19 pandemic continues, many studies have reported major changes in the utilization of healthcare services because of measures such as lockdowns and stay-at-home orders. These changes include large reductions in hospital visits, and some selective increases in online health services [8-10]. However, minimal data are available on the relationship between hospital visits and online health services over the same period, especially with quantitative time-trend evidence. Examining the short-term and long-term patterns of changes in hospital volume and online health services during the COVID-19 pandemic is valuable. The extent and effects of substitution with online healthcare services for different kinds of diseases also require further investigation.

Beijing is a megacity with the most extensive internet accessibility (77.8%) [11] and the most famous hospitals compared with other parts of China. After the COVID-19 pandemic, many patients were unable to access hospitals in Beijing owing to the lockdown policies. In response, online health services and an internet-based health system have been utilized by hospitals in addition to third-party service platforms, such as GoodDoctor (www.haodf.com). Physicians are active in spending their free time on these platforms to provide patients with high-quality online health consultations. Online consultations refer to patients using the Internet to communicate with physicians in real-time through online text, pictures, video, audio, etc.

Figure 1 depicts the epidemic trends and significant changes in the COVID-19 outbreak in Beijing. The first confirmed case of COVID-19 in Beijing occurred on January 20, 2020. Since then, the virus has spread across Beijing. Beijing launched the first-level response mechanism for public health emergencies on January 24, 2020. To respond to public health emergencies promptly, China has launched a tiered response mechanism based on different pandemic levels and hazards. The first-level response mechanism is unified deployment by provincial headquarters according to the decisions of The State Council. The second-level response mechanism refers to deployment by the provincial headquarters. The third-level response mechanism refers to deployment by the headquarters of the prefect-level city. Two peaks occurred on February 2 and March 23 in the early days. Afterwards, the epidemic entered a normalization stage, with no new cases for a long time. On June 11, a 52-year-old man from Xinfadi Market in Beijing was diagnosed with COVID-19. After a 56-day zero-new-case interval, marking the beginning of "the second epidemic," new cases increased sharply on June 13. The number of new cases in a single day reached the highest value of 36 cases. Beijing's response mechanism for public-health emergencies was adjusted from level 3 to level 2 on June 16. This phenomenon led to a fast downward slope with the control of the Chinese government. The epidemic in Beijing has gradually stabilized since then. As of December 31, 987 confirmed cases

For peer review only

and 9 deaths had been reported. In the present study, the change in short-term refers to the instantaneous change in medical-service utilization at the beginning of the pandemic from the end of December 2019 to February 2020, whereas long-term analysis refers to a snapshot change within the first year of the pandemic.

[Insert Figure 1 here]

This study was designed to compare and analyze the changing trend of online consultation and face-to-face healthcare volume, as well as the change in different disease types during the pandemic. The research illustrates the speed, scale, and reception of online-consultation utilization by patients and physicians, as well as the complementary trends in online and face-to-face health services. The results may help provide insights into the planning of future healthcare resources during the outbreak of infectious diseases.

Methods

Study Design

We conducted an interrupted time-series analysis to estimate changes in face-to-face and online healthcare utilization before and after the COVID-19 outbreak. Simple pre-post designs perform before–after comparisons by estimating the change from a single pre-intervention time point to a single post-intervention time point. However, these have poor internal validity as they cannot exclude underlying trends as a cause for any change [12]. Conversely, interrupted time series (ITS) use multiple observations in the pre-intervention and post-intervention periods, thus offer thereby offering a quasi-experimental research design with a potentially high degree of internal validity [13,14]. We treated COVID-19 as the intervention, and 2019 and 2020 as the pre-intervention and post-intervention periods respectively, to analyze the change in face-to-face and online healthcare utilization after the COVID-19 outbreak.

Setting

Monthly hospital-grade healthcare-service data from January 2019 to December 2020 were provided by Beijing Hospitals Authority. Beijing municipal hospitals refer to the public hospitals managed by the Beijing Hospitals Authority of Beijing Municipal Health Commission.

Participants

A total of 22 hospitals were selected in this study, and they covered all tertiary first-class

For peer review only

public hospitals managed by the Beijing Hospital Authority. Thirteen were general hospitals and nine were specialized hospitals (obstetrics and gynecology, pediatrics, oncology, stomatology, chest, psychiatry, traditional Chinese medicine, etc.). We collected outpatient and emergency visits, the number of discharges, online consultation volume from all 22 Beijing municipal hospitals, and patients including adults and children. Discharged patients refer to patients who are transferred from outpatient or emergency departments to inpatient departments, including acute, chronic, and severe cases.

Patient and Public Involvement

This work was an observational cohort study using publicly available data from the Beijing Hospital Authority and GoodDoctor website. Patients were not involved in our design, conduct, or reporting.

Data sources

First, outpatient and emergency visits, as well as the number of discharges in 2019 and 2020 from all 22 Beijing municipal hospitals were used as the overall face-to-face healthcare utilization outcomes. Four diseases including acute myocardial infarction (AMI), lung cancer (LC), disk disease, and Parkinson's disease were selected to analyze changes in healthcare-service utilization for different disease severities. These four diseases belonged to the 20 monitored diseases of the Beijing Municipal Health Commission. We used The International Statistical Classification of Diseases and Related Health Problems, Tenth Revision (ICD-10) to identify the principal diagnoses based on the main diagnosis of the electronic medical-record system of hospitals. Owing to data-sharing restrictions, the monthly number of discharges for these four diseases was collected from July 2019 to December 2020.

Second, online consultation data were obtained from GoodDoctor (www.haodf.com). GoodDoctor is the largest and longest-running online physician–patient communication platform in China [15]. It was founded in 2006. As of October 2021, it has collected information on 860 000 physicians from 9,780 regular hospitals in China, among which more than 73% are physicians in tertiary hospitals [16]. The 22 hospitals are all included in the GoodDoctor platform, covering more than 80% of outpatient doctors of these hospitals. A large number of patients turned to this platform for help after the COVID-19 pandemic outbreak, largely representing the trend in the utilization of online consultations during the pandemic.

The platform provides historical data of online consultations from 2010 to the present. We collected the daily online consultation data of the abovementioned 22 public hospitals in 2019

For peer review only

and 2020 and screened out the online-consultation volume data for AMI, LC, disk disease, and Parkinson's disease based on disease information. Figure 2 shows the content of the online consultation provided by this platform. We used Java language to design a web crawler and capture daily online-consultation information from web pages. Specific extracted fields include the date, website link, title, disease description, disease name, expected treatment, the duration of illness, consultation text of the patient and physician, and the name, title, department, patient voting numbers, online-consultation volume, and satisfaction of online service physicians. The final sample size collected was 70,086 for 2019 and 144,651 for 2020.

[Insert Figure 2 here]

Statistical methods

We fitted an ordinary least squares model by using either regress or Newey (with lag specified) with monthly service volume as the primary outcome of interest. We produced Newey–West standard errors to handle autocorrelation. We treated COVID-19 as the intervention, and 2019 and 2020 as the pre-intervention and post-intervention periods, respectively. The ITSA regression model is expressed as follows:

$$Y_t = \beta_0 + \beta_1 T_t + \beta_2 X_t + \beta_3 X_t T_t + \varepsilon_t$$

where Y_t is the effect variable, including outpatient and emergency visits, discharge volume, and online-consultation volume; T_t is the time since the start of the study, X_t is a dummy variable representing the intervention (pre-intervention period 0, otherwise 1), and X_tT_t is the interaction term; β_0 is the intercept or starting level of the outcome variable; β1 is the slope or trajectory of the outcome variable until the COVID-19 outbreak; β_2 is the change in the level of outcome that occurred in the period immediately following the COVID-19 outbreak (compared with the counterfactual); and β_3 is the difference between the pre-pandemic and pandemic slopes of the outcome. Accordingly, we searched for significant p-values in β_2 to indicate an immediate treatment effect or in β_3 to indicate a treatment effect over time.

Moreover, we determined the impact of COVID-19 on the utilization of healthcare services for different diseases.

We stratified models by disease type restricted to (1) AMI, (2) LC, (3) disk disease, and (4) Parkinson's disease. They were included in the 20 monitored diseases of Beijing Hospital Authority with clear diagnostic criteria. These four diseases had a certain number of patients online and face-to-face. AMI and LC were considered severe cases, whereas disk disease and Parkinson's disease were considered mild cases in the research. All statistical analyses were conducted using Stata 15.0 and SPSS 20.0.

For peer review only

Bias

Although interrupted time series has high internal validity, external confounding factors cannot be controlled. Since the outbreak of COVID-19 across the entirety of China in 2020, we cannot find an effective control group. A certain confounding bias existed in this study.

Result

1. Impact of the COVID-19 pandemic on the utilization of healthcare services

Table 1 shows the descriptive statistical indicators of the face-to-face outpatient and emergency visits, discharges, and online consultations in 2019 and 2020. Compared with 2019, the utilization of face-to-face healthcare services decreased in 2020. The monthly average outpatient and emergency visits, as well as discharges, decreased by 36.33% and 35.75%, respectively. Compared with 2019, the utilization of online consultations increased in 2020. The monthly average online consultations increased by 90.06%.

Table 1 Healthcare-service utilization in 2020 compared with 2019 (ten thousand)

	2019 (before the COVID-19 outbreak)			2020 (after the COVID-19 outbreak)		
	face-to-face outpatient and emergency visits	face-to-face discharges	online consultation	face-to-face outpatient and emergency visits	face-to-face discharges	online consultation
Mean	270.179	8.677	0.634	172.018	5.575	1.205
Std.	25.779	0.918	0.054	54.554	2.160	0.238
Median	274.391	8.956	0.611	179.315	5.996	1.272
Max	311.549	9.559	0.754	243.728	8.292	1.450

Figure 3 shows the percentage changes in healthcare-service utilization in Beijing. Compared with the same period in 2019, we observed lower monthly face-to-face outpatient and emergency visits, as well as discharges in 2020. The percentage change frequency for both was the greatest in March, with 66.97% and 75.17% decrease, respectively. The overall change decreases thereafter. Notably, when affected by "the second epidemic," face-to-face healthcare services increased slightly again, but utilization in July fell at a higher rate than in June. Online

For peer review only

consultations in all months except January were higher than in the same period in 2019. The overall change increased. The greatest decrease was observed in August. Figure 3 shows that in the early days of the pandemic (January to June), the utilization of online and face-to-face healthcare services showed obvious complementary trends. Afterwards, the growth rate of online consultations remained relatively high, whereas the decline rate of face-to-face healthcare services gradually decreased. This finding suggested that the utilization of face-to-face healthcare services is gradually returning to normal levels as COVID-19 fades away, whereas online consultations is maintaining a high growth rate.

[Insert Figure 3 here]

2. Interrupted time-series analysis

Changes in overall online and face-to-face healthcare services

Table 2 shows a highly significant reduction in the mean number of outpatient and emergency visits, as well as discharges, which dropped by 1,755,930 cases (P<0.01) and 59,200 cases (P<0.01), respectively. Before the COVID-19 outbreak, face-to-face healthcare services (including outpatient and emergency visits and discharges) increased (Figure 4). Soon after the COVID-19 outbreak, the slope of this trend line significantly increased, but the trend was not statistically significant. Online consultations also rose by 3,650 cases (P<0.05), followed by a significant increase in the monthly trend of utilization (relative to the pre-pandemic) of 580 cases.

Table 2 Impact of COVID-19 on the utilization of online and face-to-face healthcare services (ten thousand)

	Medical utilization reduction after the COVID-19 outbreak (intercept)	P value	Average monthly medical utilization decline after the COVID-19 outbreak (potential trend)	P value
face-to-face outpatient and emergency visits	-175.593 (27.247*)	<0.01	7.357 (4.454)	>0.1
face-to-face discharges	-5.920 (1.608)	<0.01	0.283 (0.217)	>0.1
online consultation	0.365 (0.143)	<0.05	0.058 (0.019)	<0.01

Standard error in parentheses.

For peer review only

* p<0.1, **p<0.05, ***p<0.01.

[Insert Figure 4 here]

Changes in face-to-face healthcare services by type of disease

Table 3 summarizes the effect of COVID-19 on face-to-face healthcare services for different diseases. The COVID-19 outbreak was associated with a rapid decline in face-to-face healthcare services for different diseases. From the perspective of immediate effect, we identified a significant drop in healthcare services for the four diseases. From the perspective of long-term effects, AMI, LC, and disk disease services showed a nonsignificant stepwise increase (the slope of the trend line increased). Simultaneously, the monthly trend of utilization for Parkinson's disease significantly increased (relative to the pre-pandemic period) (Figure 5).

Table 3 Impact of COVID-19 on the utilization of face-to-face healthcare services and online consultation by type of disease

	Medical utilization reduction after the COVID-19 outbreak (intercept)		Average monthly medical utilization decline after the COVID-19 outbreak (potential trend)	
	face-to-face healthcare services	online consultation	face-to-face healthcare services	online consultation
acute myocardial infarction	-174.405 (84.060) *	-5.998 (3.511)	12.438 (11.540)	1.633 (0.472) ***
lung cancer	-2,502.02 (367.118) ***	-11.832 (19.413)	80.508 (80.480)	14.360 (3.079) ***
disk disease	-375.560 (72.369) ***	63.118 (11.955) ***	29.163 (16.876)	3.402 (1.851) *
Parkinson's disease	-205.229 (47.852) ***	24.625 (10.349) **	12.669 (4.892) **	1.772 (1.335)

Standard error in parentheses.

* p<0.1, **p<0.05, ***p<0.01.

[Insert Figure 5 here]

Changes in online consultations by type of disease

Table 3 summarizes the effect of COVID-19 on online consultations for different diseases. The COVID-19 outbreak was associated with a rapid rise in online consultations for different

For peer review only

diseases. From the perspective of immediate effect, we identified a significant increase in healthcare services for disk disease and Parkinson's disease. Conversely, online consultations for AMI and LC showed a small, nonsignificant decrease. From the perspective of long-term effects, online consultations for AMI, LC, and disk disease increased monthly at a rate of 1,633, 14,360, and 3,402, respectively after the COVID-19 outbreak (Figure 6).

[Insert Figure 6 here]

3. **Impact of COVID-19 pandemic on online-service physicians' volume**

As affected by COVID-19, the monthly volume of online services provided by physicians in 2020 was significantly higher than that in 2019, except for January. On average, more than 1,300 unique physicians from 22 hospitals in Beijing provided online-consultation services per month in 2020, which was 35.3% higher than that in 2019. This finding suggested that the supply of online consultations greatly increased after the COVID-19 outbreak (Figure 7).

[Insert Figure 7 here]

Discussion

Based on monthly treatment data from 22 public hospitals in Beijing, obvious complementary trends existed in online and face-to-face healthcare services during the COVID-19 pandemic. We found that public hospitals in Beijing experienced substantial losses in face-to-face healthcare and substantial increases in online-consultation services when the COVID-19 pandemic began in China in 2020. The interrupted time-series models revealed that after the COVID-19 outbreak, the considerable reduction in face-to-face healthcare services was a short-term effect, whereas the increase in online consultation was a long-term one. As only one year of the pandemic was examined in the current study, the long-term effects herein referred to change trends within one year. In other words, this study is a snapshot. Simultaneously, the effects of COVID-19 on healthcare services for different diseases varied. Noncritical-disease patients showed greater reductions in face-to-face service utilization and chose online consultations immediately after the COVID-19 lockdown. However, critical-disease patients chose face-to-face treatment first, and slowly moved online over time. The volume of online services provided by physicians also significantly increased as a result of COVID-19.

Our study is consistent with previous reports on hospital healthcare-service changes during the COVID-19 pandemic [17,18]. A systematic review from the UK analyzed 81 studies and reported that the utilization of healthcare in care facilities sharply decreased by about a third during the pandemic period, with greater reductions among people with less severe illnesses [17].

For peer review only

Additionally, according to a study from one large health system (NYU Langone Health) in the United States, telemedicine visits increased from 102.4 daily to 801.6 daily between March 2 and April 14 2020, with a concomitant decline in in-person visits by over 80%. We also showed that online consultation underwent sustained growth owing to the impact of COVID-19, whereas face-to-face healthcare services showed a short-term decline. An important reason for the decline in face-to-face visits was that rigorous public health measures were implemented in China during the COVID-19 epidemic [19]. Some medical resources responded to the outbreak. The number of outpatient and emergency visits to 22 municipal hospitals in Beijing decreased by 36.33% in 2020. More medical personnel provided epidemic prevention and control services, such as nucleic acid tests and vaccines. Furthermore, potential risk factors in the hospital environment made people anxious and worried. A survey has shown that 20% of pregnant women fear going to a hospital for consultation, and more than 40% fear going to a hospital for check-ups [20].

A rapid decrease in overall face-to-face discharge admissions was observed for four different diseases across 22 tertiary public hospitals in Beijing after the COVID-19 lockdown. A study performed in Germany revealed a similar reduction in traditional face-to-face healthcare services for different diseases [21]. That study found that hospital admissions for malignant cancer treatments and AMI decreased after the lockdown announcement. However, a difference was that our analysis also showed the impact of the epidemic on diseases with varied severity. We also examined the changes in online consultations for different diseases. We found that different diseases showed different online change trends. Online consultations for critical illnesses (such as AMI, and LC) did not increase immediately after the outbreak but gradually increased in the long-term study. A study has shown that telemedicine can be used to reach patients with severe disease burdens [22]. Conversely, we observed that noncritical clinical situations (such as disk disease and Parkinson's disease) considerably and immediately increased, and this trend has been maintained.

Several reasons may explain the dramatic decrease in face-to-face healthcare services after the COVID-19 pandemic. First, lockdown policy and population mobility control measures were implemented in China to mitigate the spread of the pandemic. For instance, the community implemented closed management, hospital services were limited, and non-emergency services had to be reserved in advance and staggered used in Beijing during the pandemic period. This situation may have reduced the availability and accessibility of face-to-face healthcare services. Second, some patients chose to postpone their treatment to prevent cross-infection in the hospital. Most healthcare resources were relocated to manage the pandemic. This setup may trigger attitudes toward delaying less urgent cases by the patient and the healthcare system. Third, some health demands may have been eliminated. For instance, many patients with self-resolving diseases chose not to avail of face-to-face treatment, such as for colds, diarrhea,

For peer review only

etc. [23,24]. The more important point was that some health demands may have been transferred online. Abundant internet diagnosis and treatment services were supplied by medical institutions in Beijing during the pandemic, such as online consultation, instant messaging, synchronous chat, video conferencing, etc. Online consultation can span time and space. The physician and patient do not have to meet each other within a physical space, subsequently decreasing the requirement for face-to-face travel, appointment-related concerns, and the risk of face-to-face infection. Some patients could migrate to online consultation to meet their health needs, which can explain the significantly increased volume of online consultations.

On one hand, our results indicated that mass migration to online consultations occurred shortly after the COVID-19 pandemic. On the other hand, the migration of critically ill patients was slower than that of noncritically ill ones because the latter relied more on facility treatment. For example, the efficiency of the care pathway at every step matters very much for AMI patients [25]. Chemotherapy, radiation therapy, surgery, and molecular targeted therapy are keystones in the treatment of early and locally advanced LC with good prognosis [26]. These conditions rely on face-to-face operations and inspections, and online consultations cannot provide these services, which is why the online-consultation volume for critical illnesses did not increase immediately after the outbreak. Additionally, we found that the online-consultation volume for three diseases, except for Parkinson's, continuously increased. This phenomenon can be explained by the fact that the COVID-19 pandemic accelerated the popularity of online healthcare, thereby increasing the awareness and acceptance of the public regarding online healthcare.

Our study also found that the pandemic led to a significant increase in online patients and also facilitated the online migration of physicians. The number of physicians offering services online in 2020 was much higher than that over the same period in 2019, except for January. This finding showed that physicians paid attention to providing online healthcare services.

With the COVID-19 pandemic, the supply of face-to-face healthcare services was relatively insufficient, resulting in partial healthcare-service disruption. The COVID-19 pandemic resulted in decreased supply of face-to-face healthcare services, which was part of the healthcare-service disruption. To reduce the public-health impact, we put forward two suggestions: first, we should designate some part of healthcare resources to online provision. Moreover, we should rapidly establish a collaborative model of face-to-face and online healthcare services in public-health emergencies to meet the demand of patients. Online consultations and telemedicine can alleviate geographic and physician shortages, expand healthcare accessibility, and improve the quality of healthcare provision. Therefore, online healthcare services can serve as an important supplement to traditional healthcare services, optimize health resource allocation, and improve service efficiency, especially for noncritically ill patients.

For peer review only

However, not all diseases are suitable for online consultations. Face-to-face healthcare services are necessary and irreplaceable, such as physical examinations, diagnoses, and treatment of difficult and severe diseases. Different healthcare support measures should be formulated according to the characteristics of different diseases. For critical cases, face-to-face channels ought to be opened for diagnosis and treatment to ensure that patients can receive face-to-face treatment in a timely manner. Studies indicate that 9.1% of patients experienced a delay in LC treatment, and half of AMI patients did not reach out to the hospital at all during the COVID-19 pandemic [25,27]. For critical cases, delaying or stopping treatment can have a significant negative impact on the physical and mental health of critically ill patients. A timely diagnosis and improved treatment strategy will facilitate the health of patients [26]. For mild cases, cooperation among the government, health facilities, and third-party platforms should be enhanced. Online healthcare services such as telemedicine, online consultation, psychological counseling, and chronic disease revisits ought to be expanded. Expanding the supply of online healthcare resources and helping patients migrate online to receive healthcare services are crucial. Meanwhile, a comparison between the quality of online consultation and face-to-face care is worth discussing. An in-depth review shows that online consultation is a useful, high-quality, and safe option for the vast majority of pediatric neurology patients [28]. A study has shown that no significant difference exists between the satisfaction of online counseling and face-to-face healthcare [29]. However, studies have also shown that people still worry about the reliability of online healthcare [30].

Finally, our work confirmed that the increase in online consultations was a persistent phenomenon during the research period. A new normal deployment of online healthcare services occurred in the pandemic and policy-enabling environment. The development of online consultation should be encouraged because of the advantages of online consultation across time and space. New technologies such as 5G technology, sensing techniques, and artificial intelligence are bound to be fully exploited for online consultation. Innovating the long-term synergistic modalities of online and face-to-face healthcare services is important to promote the connection among high-quality medical resources at all levels.

This study had some limitations. First, we studied the complementary trend of online and face-to-face health service utilization. Whether the change in service delivery caused an impact on the quality or the equivalency of care delivery requires further study. Second, to observe the changes in healthcare-service utilization, the behavior of patients with diseases of varied severity, i.e., AMI, LC, disk disease, and Parkinson’s disease, was selected in this paper. Further studies are needed to determine whether this phenomenon can be extended to other diseases. The heterogeneity of the data across the hospitals was also not explored. Third, the usage of GoodDoctor cannot cover all the online-consultation behavior. Although GoodDoctor is the largest online consultant platform in China, some other online-consultant platforms are available

For peer review only

in the industry. We choose the GoodDoctor platform by considering that the 22 surveyed hospitals all had a certain number of doctors in the platform, which was representative to a certain extent. Finally, this study was only a snapshot capturing the first year of the pandemic response. Comprehensive research over a longer period is required.

Conclusion

Face-to-face healthcare services dropped precipitously and online consultations rose sharply in 22 tertiary public hospitals in Beijing during the COVID-19 pandemic. However, face-to-face healthcare services showed a relatively fast recovery, and online consultations continued to grow. The changing trend in online-consultation volume varied by disease, which was characterized by a nonsignificant increase in the short term and a significant increase in the long term regarding critical cases after the outbreak, as well as a significant increase in noncritical cases in the long short term. The availability of online consultations should be encouraged during pandemics. Particular attention should be given to severely ill patients who need adequate and prompt face-to-face hospital care.

Author affiliations

1 The Seventh Affiliated Hospital, Sun Yat-sen University, Guangdong, China

2 School of Public Health, Capital Medical University, Beijing, China

Contributors The research idea originated with CM, but all authors designed the study. SZ directed the data analyses. CM and SZ co-wrote this manuscript.

Funding This study has been funded by the Natural Science Foundation of Beijing Municipality (grant number: 9222993).

Competing interests We have read and understood the BMJ policy on the declaration of interests. All authors report no competing interests.

For peer review only

Patient and public involvement statement Patients and the public were not involved in the design or conduct of this study. The study used publicly available data from GoodDoctor, which has signed privacy policies with users.

Patient consent for publication Not applicable.

Ethics approval It was approved by the ethics committee of Capital Medical University (approval No. Z2020SY126).

Provenance and peer review Not commissioned; externally peer-reviewed.

Data availability statement Data are available on reasonable request.

Abbreviations

AMI: Acute Myocardial Infarction

LC: Lung Cancer

References

1 Moynihan R, Sanders S, Michaleff Z A, et al. Impact of COVID-19 pandemic on utilisation of healthcare services: a systematic review[J]. BMJ Open, 2021, 11(3): e045343.

2 Xu S, Glenn S, Sy L, et al. Impact of the COVID-19 Pandemic on Health Care Utilization in a Large Integrated Health Care System: Retrospective Cohort Study. J Med Internet Res. 2021 Apr 29;23(4): e26558.

3 Hartnett KP, Kite-Powell A, DeVies J, et al. National Syndromic Surveillance Program Community of Practice. Impact of the COVID-19 Pandemic on Emergency Department Visits - United States, January 1, 2019-May 30, 2020. MMWR Morb Mortal Wkly Rep. 2020 Jun 12;69(23):699-704.

4 Lukas H, Xu C, Yu Y, et al. Emerging Telemedicine Tools for Remote COVID-19 Diagnosis, Monitoring, and Management. ACS Nano. 2020 Dec 22;14(12):16180-16193.

For peer review only

5 Bokolo Anthony Jnr. Use of Telemedicine and Virtual Care for Remote Treatment in Response to COVID-19 Pandemic. J Med Syst. 2020 Jun 15;44(7):132.

6 Garcia S, Albaghdadi M S, Meraj P M, et al. Reduction in ST-Segment Elevation Cardiac Catheterization Laboratory Activations in the United States During COVID-19 Pandemic[J]. Journal of the American College of Cardiology, 2020.

7 Mann DM, Chen J, Chunara R, et al. COVID-19 transforms health care through telemedicine: Evidence from the field. J Am Med Inform Assoc. 2020 Jul 1;27(7):1132-1135.

8 Chen Y, Cai M, Li Z, et al. Impacts of the COVID-19 Pandemic on Public Hospitals of Different Levels: Six-Month Evidence from Shanghai, China. Risk Manag Healthc Policy. 2021;14: 3635-3651.

9 Li G, Fan G, Chen Y, et al. What patients "see" doctors in online fever clinics during COVID-19 in Wuhan? J Am Med Inform Assoc. 2020 Jul 1;27(7):1067-1071.

10 Shimels T. The Trend of Health Service Utilization and Challenges Faced During the COVID-19 Pandemic at Primary Units in Addis Ababa: A Mixed-Methods Study. Health Serv Res Manag Epidemiol. 2021 Jul 8;8:23333928211031119.

11 China Internet Network Information center. Beijing, China: CNNIC, 2021. Annual Report 2021.

12 James, Lopez, Bernal, et al. The use of controls in interrupted time series studies of public health interventions.[J]. International Journal of Epidemiology, 2018.

13 Shadish W, Cook T, Campbell D. Experimental and Quasi-Experimental Designs For Generalized Causal Inference. Houghton Mifflin, 2002.

14 Miller CJ, Smith SN, Pugatch M. Experimental and quasi-experimental designs in implementation research. Psychiatry Res. 2020 Jan;283:112452.

15 Liu X, Chen M, Li J, et al. How to Manage Diversity and Enhance Team Performance: Evidence from Online Doctor Teams in China. Int J Environ Res Public Health. 2019 Dec 19;17(1):48.

16 The GoodDoctor profile, The GoodDoctor. 2022. http://www.haodf.com/info/aboutus.php

17 Moynihan R, Sanders S, Michaleff ZA, et al. Impact of COVID-19 pandemic on utilisation of healthcare services: a systematic review. BMJ Open. 2021 Mar 16;11(3): e045343.

18 Mann DM, Chen J, Chunara R, et al. COVID-19 transforms health care through telemedicine: Evidence from the field. J Am Med Inform Assoc. 2020 Jul 1;27(7):1132-1135.

19 Yang ZM, Wu MY, Lu JM, et al. Effect of COVID-19 on hospital visits in Ningbo, China: an interrupted time-series analysis. Int J Qual Health Care. 2021 May 7;33(2): mzab078.

For peer review only

20 Wu H, Sun W, Huang X, et al. Online Antenatal Care During the COVID-19 Pandemic: Opportunities and Challenges. J Med Internet Res. 2020 Jul 22;22(7): e19916

21 Kapsner LA, Kampf MO, Seuchter SA, et al. Reduced Rate of Inpatient Hospital Admissions in 14 German University Hospitals During the COVID-19 Lockdown. Front Public Health. 2021 Jan 13;8: 594117.

22 Shehan JN, Agarwal P, Danis DO 3rd, et al. Effects of COVID-19 on telemedicine practice patterns in outpatient otolaryngology. Am J Otolaryngol. 2021 Nov-Dec;42(6):103044.

23 Giannouchos TV, Biskupiak J, Moss MJ, et al. Trends in outpatient emergency department visits during the COVID-19 pandemic at a large, urban, academic hospital system. Am J Emerg Med. 2021 Feb;40:20-26.

24 Meng H, Ji LN, Huang J, et al. Analysis of the changes and characteristics of pediatric outpatient visits in a general hospital in Beijing before and after the COVID-19 pandemic. Beijing Da Xue Xue Bao Yi Xue Ban. 2021 Oct 18;53(5):952-956. Chinese.

25 German University Hospitals During the COVID-19 Lockdown. Front Public Health. 2021 Jan 13;8: 594117.

26 De Rosa S, Spaccarotella C, Basso C, et al. Reduction of hospitalizations for myocardial infarction in Italy in the COVID-19 era. Eur Heart J. 2020 Jun 7;41(22):2083-2088.

27 Sha Z, Chang K, Mi J, Liang Z, et al. The impact of the COVID-19 pandemic on lung cancer patients. Ann Palliat Med. 2020 Sep;9(5):3373-3378.

28 Joshi CN, Yang ML, Eschbach K, et al. Quality and Safety Analysis of 2,999 Telemedicine Encounters During the COVID-19 Pandemic. Neurol Clin Pract. 2021 Apr;11(2): e73-e82.

29 Fieux M, Duret S, Bawazeer N, et al. Telemedicine for ENT: Effect on quality of care during Covid-19 pandemic. Eur Ann Otorhinolaryngol Head Neck Dis. 2020 Sep;137(4):257-261.

30 Wu H, Sun W, Huang X, et al. Online Antenatal Care During the COVID-19 Pandemic: Opportunities and Challenges. J Med Internet Res. 2020 Jul 22;22(7): e19916.

Figure legends

Figure 1 COVID-19 epidemic trend in Beijing

Figure 2 Information on online consultation provided by this platform

For peer review only

Figure 3 Percentage change in healthcare-service utilization in 2020 compared with that in 2019

Figure 4 Effect of COVID-19 intervention on face-to-face and online healthcare utilization. Scatter plots represent monthly medical utilization. The black line of best fit represents medical utilization across estimated by post-regression. The vertical black line is the intervention point. (a) Face-to-face outpatient and emergency visits, (b) face-to-face discharges, and (c) online consultation.

Figure 5 Effect of COVID-19 intervention on face-to-face healthcare utilization by type of disease. Scatter plots represent medical utilization monthly. The black line of best fit represents medical utilization across estimated by post-regression. The vertical black line is the intervention point. (a)AMI, (b) LC, (c) disk disease, and (d) Parkinson's disease.

Figure 6 Effect of COVID-19 intervention on online-consultation utilization by disease type. Scatter plots represent monthly medical utilization. The black line of best fit represents medical utilization across estimated by post-regression. The vertical black line is the intervention point. (a)AMI, (b) LC, (c) disk disease, and (d) Parkinson's disease.

Figure 7 Changes in online physician volume in 2020 compared with that in 2019. The dotted line is the mean.

For peer review only

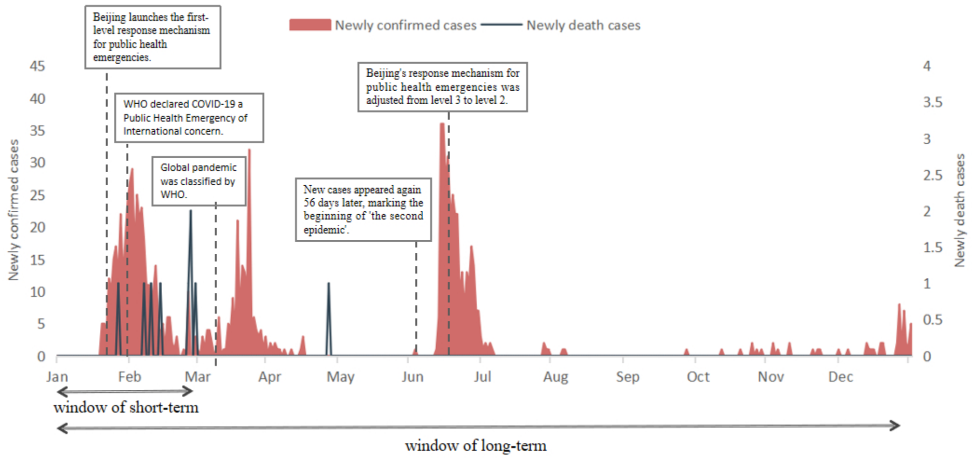

Figure 1 COVID-19 epidemic trend in Beijing

478x244mm (57 x 57 DPI)

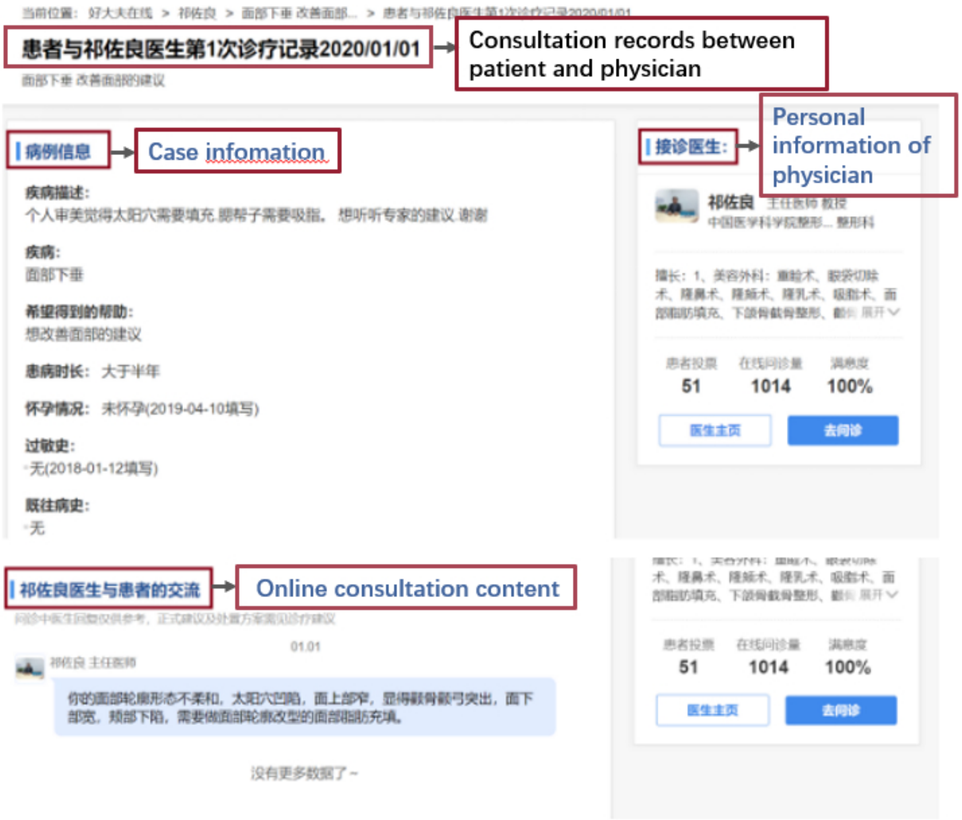

Figure 2 The information of the online consultation provided by GoodDoctor platform

331x283mm (57 x 57 DPI)

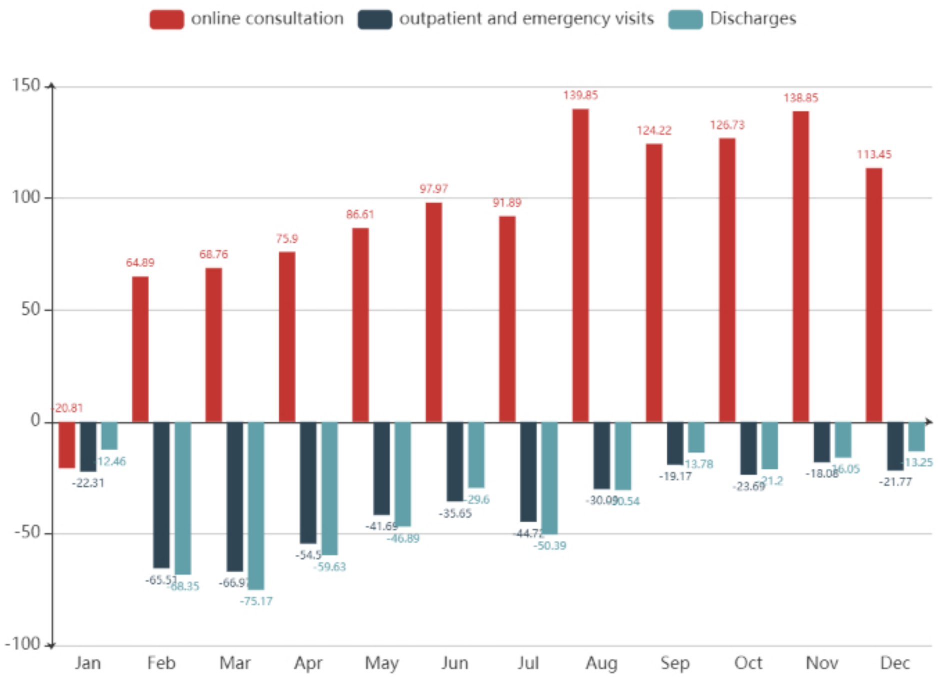

Figure 3 Percentage change in healthcare service utilization in 2020 compared with 2019 (%)

254x175mm (72 x 72 DPI)

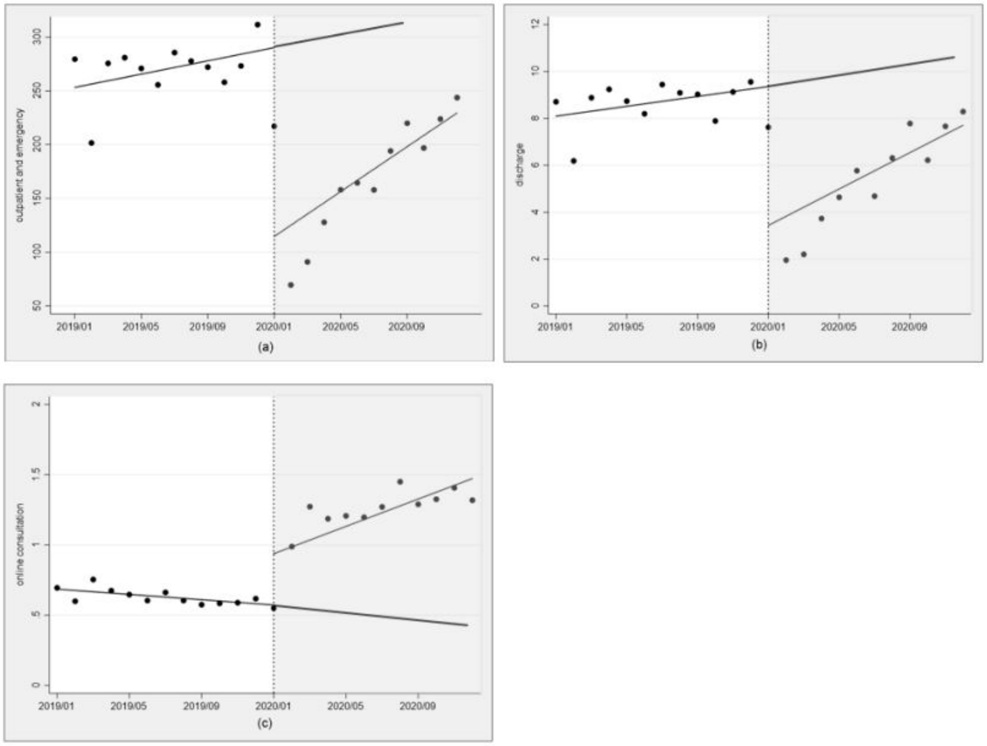

Figure 4 The effect of COVID-19 intervention on face-to-face and online healthcare utilization. Scatter plots represent monthly medical utilization. The black line of best fit represents medical utilization across estimated by post-regression. The vertical black line is the intervention point. (a) face-to-face outpatient and emergency visits; (b) face-to-face discharges; (c) online consultation.

143x108mm (144 x 144 DPI)

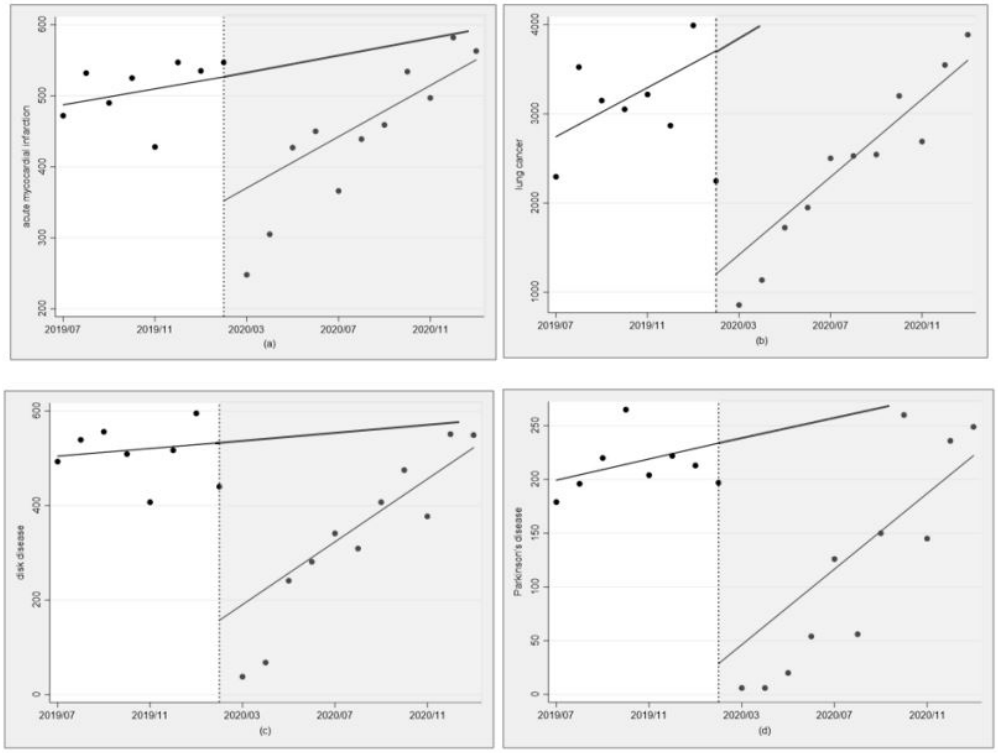

Figure 5 The effect of COVID-19 intervention on face-to-face healthcare utilization by type of disease. Scatter plots represent medical utilization monthly. The black line of best fit represents medical utilization across estimated by post-regression. The vertical black line is the intervention point. (a) AMI; (b)LC; (c) disk disease; (d) Parkinson's disease.

142x108mm (144 x 144 DPI)

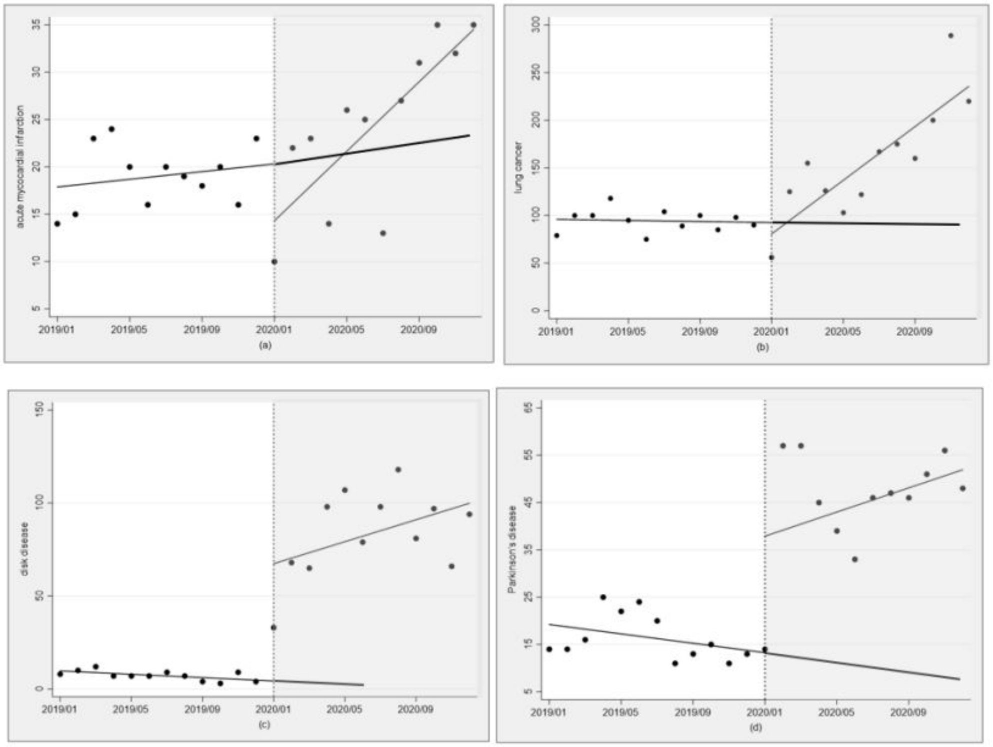

Figure 6 The effect of COVID-19 intervention on online consultations utilization by type of disease. Scatter plots represent medical utilization monthly. The black line of best fit represents medical utilization across estimated by post-regression. The vertical black line is the intervention point. (a)AMI; (b) LC; (c) disk disease; (d) Parkinson's disease.

144x108mm (144 x 144 DPI)

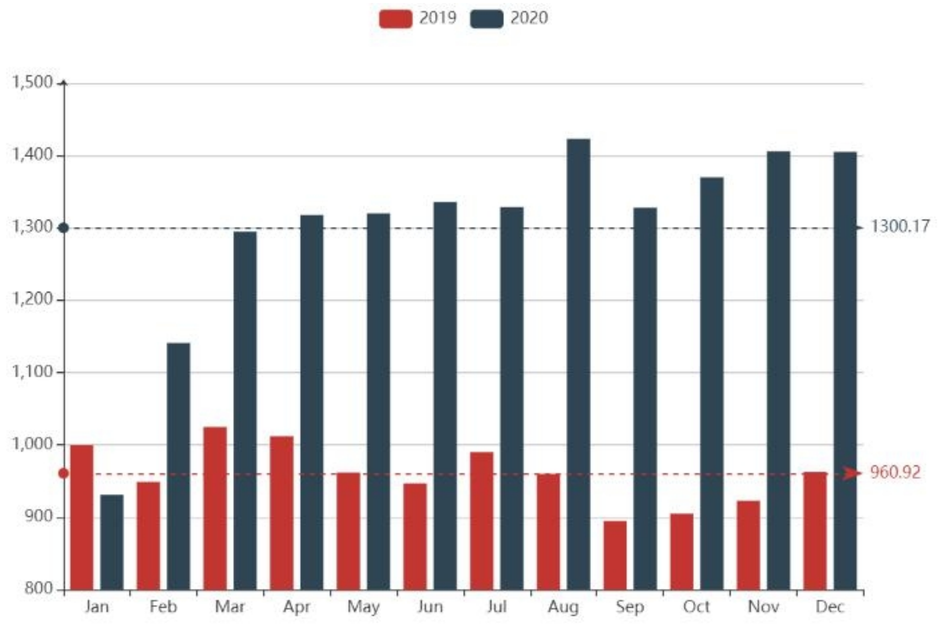

Figure 7 Changes in online physician volume in 2020 compared with 2019 (%). The dotted line is the mean.

131x88mm (144 x 144 DPI)

STROBE Statement—checklist of items that should be included in reports of observational studies

	Item No.	Recommendation	Page No.	Relevant text from manuscript
**Title and abstract**	1	(*a*) Indicate the study's design with a commonly used term in the title or the abstract	Page 1 line 1-2	COVID-19, interrupted time-series study
		(*b*) Provide in the abstract an informative and balanced summary of what was done and what was found	Page 1 line 9-32, Page 2 line 1-2	We report the main research contents, results and conclusions in the abstract. We found that the online and offline trends were complementary during the pandemic. Obvious complementary trends in online and face-to-face healthcare services existed during the COVID-19 pandemic through the time series analysis. And different changes in healthcare utilization were shown for different diseases.
**Introduction**				
Background/rationale	2	Explain the scientific background and rationale for the investigation being reported	Page 2 line 17-24, Page 3 line 2-30	In the context of the COVID-19 pandemic, fear of infection and reduced availability of healthcare services has led to decreased non-COVID healthcare utilization in hospitals. And online health services have occurred in many countries to relieve the pressure on hospitals and to prevent cross-infection. We described the pandemic situation in Beijing.
Objectives	3	State specific objectives, including any prespecified hypotheses	Page 3 line 17-24	This study was designed to compare and analyze the changing trend of online consultation and face-to-face healthcare volume, as well as the change in different disease types during the pandemic. The research illustrates the speed, scale, and reception of online-consultation utilization by patients and physicians, as

For peer review only - http://bmjopen.bmj.com/site/about/guidelines.xhtml

				well as the complementary trends in online and face-to-face health services.
**Methods**				
Study design	4	Present key elements of study design early in the paper	Page 4 line 5-14	We conducted an interrupted time-series analysis. We treated COVID-19 as the intervention, and 2019 and 2020 as the pre- and post-intervention periods respectively, to analyze the change in face-to-face and online healthcare utilization after the COVID-19 outbreak.
Setting	5	Describe the setting, locations, and relevant dates, including periods of recruitment, exposure, follow-up, and data collection	Page 4 line 17-19	Monthly hospital-grade healthcare-service data from January 2019 to December 2020 were provided by Beijing Hospitals Authority. Beijing municipal hospitals refer to the public hospitals managed by the Beijing Hospitals Authority of Beijing Municipal Health Commission.
Participants	6	(*a*) *Cohort study*—Give the eligibility criteria, and the sources and methods of selection of participants. Describe methods of follow-up<br><br>*Case-control study*—Give the eligibility criteria, and the sources and methods of case ascertainment and control selection. Give the rationale for the choice of cases and controls<br><br>*Cross-sectional study*—Give the eligibility criteria, and the sources and methods of selection of participants	Page 4 line 22-27	A total of 22 hospitals were selected in this study, and they covered all tertiary first-class public hospitals managed by the Beijing Hospital Authority. Thirteen were general hospitals and nine were specialized hospitals (obstetrics and gynecology, pediatrics, oncology, stomatology, chest, psychiatry, traditional Chinese medicine, etc.).
		(*b*) *Cohort study*—For matched studies, give matching criteria and number of exposed and unexposed		N/A

For peer review only

For peer review only - http://bmjopen.bmj.com/site/about/guidelines.xhtml

		Case-control study—For matched studies, give matching criteria and the number of controls per case		
Variables	7	Clearly define all outcomes, exposures, predictors, potential confounders, and effect modifiers. Give diagnostic criteria, if applicable	Page 6 line 1-5	We fitted an ordinary least squares model by using either regress or Newey (with lag specified) with monthly service volume as the primary outcome of interest. We produced Newey–West standard errors to handle autocorrelation. We treated COVID-19 as the intervention, and 2019 and 2020 as the pre-intervention and post-intervention periods, respectively.
Data sources/ measurement	8*	For each variable of interest, give sources of data and details of methods of assessment (measurement). Describe comparability of assessment methods if there is more than one group	Page 5 line 4-31	Outpatient and emergency visits, as well as the number of discharges in 2019 and 2020 from all 22 Beijing municipal hospitals, were used as the overall face-to-face healthcare utilization outcomes. And the monthly number of discharges for AMI, LC, disk disease, and Parkinson's disease four diseases were collected from July 2019 to December 2020. Online consultation data were obtained from GoodDoctor
Bias	9	Describe any efforts to address potential sources of bias	Page 6 line 26-28	Although interrupted time series has high internal validity, external confounding factors cannot be controlled. Since the outbreak of COVID-19 across the entirety of China in 2020, we cannot find an effective control group. A certain confounding bias existed in this study.
Study size	10	Explain how the study size was arrived at	Page 4 line 22-27	A total of 22 hospitals were selected in this study, and they covered all tertiary first-class public hospitals managed by the Beijing Hospital Authority. Thirteen were general hospitals and nine were specialized hospitals (obstetrics and gynecology, pediatrics, oncology, stomatology, chest, psychiatry, traditional Chinese medicine, etc.).We collected outpatient and emergency visits, the number of discharges, online consultation volume from

For peer review only

	all 22 Beijing municipal hospitals, and patients including adults and children.

Continued on next page

For peer review only

For peer review only - http://bmjopen.bmj.com/site/about/guidelines.xhtml

Quantitative variables	11	Explain how quantitative variables were handled in the analyses. If applicable, describe which groupings were chosen and why	Page 6 line 6-15	Y_t is the effect variable, including outpatient and emergency visits, discharge volume, and online-consultation volume; Tt is the time since the start of the study, X_t is a dummy variable representing the intervention (pre-intervention period 0, otherwise 1), and X_tT_t is the interaction term; β_0 is the intercept or starting level of the outcome variable; β_1 is the slope or trajectory of the outcome variable until the COVID-19 outbreak; β_2 is the change in the level of outcome that occurred in the period immediately following the COVID-19 outbreak (compared with the counterfactual), and β3 is the difference between the pre-pandemic and pandemic slopes of the outcome. All statistical analyses were conducted using Stata 15.0 and SPSS 20.0.
Statistical methods	12	(*a*) Describe all statistical methods, including those used to control for confounding	Page 4 line 9-12	Interrupted time series (ITS) use multiple observations in the pre-intervention and post-intervention periods, and thus offer thereby offering a quasi-experimental research design with a potentially high degree of internal validity
		(*b*) Describe any methods used to examine subgroups and interactions	Page 4 line 8-10	Tt is the time since the start of the study, X_t is a dummy variable representing the intervention (pre-intervention period 0, otherwise 1), and X_tT_t is the interaction term.
		(*c*) Explain how missing data were addressed	N/A	
		(*d*) *Cohort study*—If applicable, explain how loss to follow-up was addressed<br>*Case-control study*—If applicable, explain how matching of cases and controls was addressed<br>*Cross-sectional study*—If applicable, describe analytical methods taking account of sampling strategy	N/A	

For peer review only

		(*e*) Describe any sensitivity analyses	N/A	
**Results**				
Participants	13*	(a) Report numbers of individuals at each stage of study—eg numbers potentially eligible, examined for eligibility, confirmed eligible, included in the study, completing follow-up, and analysed	N/A	There was no follow-up in our study .
		(b) Give reasons for non-participation at each stage	N/A	
		(c) Consider use of a flow diagram	N/A	
Descriptive data	14*	(a) Give characteristics of study participants (eg demographic, clinical, social) and information on exposures and potential confounders	Page 6 line 32-Page 7 line 5	We described healthcare service utilization in 2020 compared with 2019.
		(b) Indicate number of participants with missing data for each variable of interest	N/A	
		(c) *Cohort study*—Summarise follow-up time (eg, average and total amount)	N/A	
Outcome data	15*	*Cohort study*—Report numbers of outcome events or summary measures over time	Page 7 line 8-22	We described the percentage changes in healthcare-service utilization in Beijing.
		Case-control study—Report numbers in each exposure category, or summary measures of exposure	N/A	
		Cross-sectional study—Report numbers of outcome events or summary measures	N/A	
Main results	16	(*a*) Give unadjusted estimates and, if applicable, confounder-adjusted estimates and their precision (eg, 95% confidence	Page 8 line 2-Page 9 line 16	We described the results of the discontinuous time series

For peer review only

	interval). Make clear which confounders were adjusted for and why they were included	
	(*b*) Report category boundaries when continuous variables were categorized	N/A
	(*c*) If relevant, consider translating estimates of relative risk into absolute risk for a meaningful time period	N/A

Continued on next page

For peer review only

For peer review only - http://bmjopen.bmj.com/site/about/guidelines.xhtml

Other analyses	17	Report other analyses done—eg analyses of subgroups and interactions, and sensitivity analyses	Page 9 line 19-Page 10 line 4	We described the changes in online physician volume in 2020 compared with 2019.
**Discussion**				
Key results	18	Summarise key results with reference to study objectives	Page 10 line 7-20	Obvious complementary trends existed in online and face-to-face healthcare services during the COVID-19 pandemic. We found that public hospitals in Beijing experienced substantial losses in face-to-face healthcare and substantial increases in online-consultation services when the COVID-19 pandemic began in China in 2020. The interrupted time-series models revealed that after the COVID-19 outbreak, the considerable reduction in face-to-face healthcare services was a short-term effect, whereas the increase in online consultation was a long-term one.
Limitations	19	Discuss limitations of the study, taking into account sources of potential bias or imprecision. Discuss both direction and magnitude of any potential bias	Page 13 line 15-Page 13 line 27	This study had some limitations. First, we studied the complementary trend of online and face-to-face health service utilization. Whether the change in service delivery caused an impact on the quality or the equivalency of care delivery requires further study. Second, to observe the changes in healthcare-service utilization, the behaviour of patients with diseases of varied severity, i.e., AMI, LC, disk disease, and Parkinson's disease, was selected in this paper. Further studies are needed to determine whether this phenomenon can be extended to other diseases. The heterogeneity of the data across the hospitals was also not explored. Third, the usage of GoodDoctor cannot cover all the online-consultation behaviour. Finally, this study was only a snapshot capturing the first year of the pandemic response. Comprehensive research over a longer period is required.

For peer review only

Interpretation	20	Give a cautious overall interpretation of results considering objectives, limitations, multiplicity of analyses, results from similar studies, and other relevant evidence	Page 10 line 21-Page 13 line 14	The behaviour of patients with diseases of varied severity, i.e., AMI, LC, disk disease, and Parkinson's disease, was selected in this paper. Further studies are needed to determine whether this phenomenon can be extended to other diseases.
Generalisability	21	Discuss the generalisability (external validity) of the study results	Page 13 line 17-20	Further studies are needed to determine whether this phenomenon can be extended to other diseases.
**Other information**				
Funding	22	Give the source of funding and the role of the funders for the present study and, if applicable, for the original study on which the present article is based	Page 14 line 11-12	This study has been funded by the Natural Science Foundation of Beijing Municipality (grant number: 9222993).

*Give information separately for cases and controls in case-control studies and, if applicable, for exposed and unexposed groups in cohort and cross-sectional studies.

Note: An Explanation and Elaboration article discusses each checklist item and gives methodological background and published examples of transparent reporting. The STROBE checklist is best used in conjunction with this article (freely available on the Web sites of PLoS Medicine at http://www.plosmedicine.org/, Annals of Internal Medicine at http://www.annals.org/, and Epidemiology at http://www.epidem.com/). Information on the STROBE Initiative is available at www.strobe-statement.org.

For peer review only

BMJ Open

How has the COVID-19 pandemic affected the utilization of online consultation and face-to-face medical treatment? An interrupted time-series study in Beijing, China.

Journal:	*BMJ Open*
Manuscript ID	bmjopen-2022-062272.R3
Article Type:	Original research
Date Submitted by the Author:	06-Jan-2023
Complete List of Authors:	Zhang, Shan; The Seventh Affiliated Hospital Sun Yat-sen University<br>Ma, Chengyu; Capital Medical University, School of Public Health
**Primary Subject Heading**:	Public health
Secondary Subject Heading:	Health policy, Medical management, Public health
Keywords:	COVID-19, PUBLIC HEALTH, SOCIAL MEDICINE

SCHOLARONE™
Manuscripts

I, the Submitting Author has the right to grant and does grant on behalf of all authors of the Work (as defined in the below author licence), an exclusive licence and/or a non-exclusive licence for contributions from authors who are: i) UK Crown employees; ii) where BMJ has agreed a CC-BY licence shall apply, and/or iii) in accordance with the terms applicable for US Federal Government officers or employees acting as part of their official duties; on a worldwide, perpetual, irrevocable, royalty-free basis to BMJ Publishing Group Ltd ("BMJ") its licensees and where the relevant Journal is co-owned by BMJ to the co-owners of the Journal, to publish the Work in this journal and any other BMJ products and to exploit all rights, as set out in our licence.

The Submitting Author accepts and understands that any supply made under these terms is made by BMJ to the Submitting Author unless you are acting as an employee on behalf of your employer or a postgraduate student of an affiliated institution which is paying any applicable article publishing charge ("APC") for Open Access articles. Where the Submitting Author wishes to make the Work available on an Open Access basis (and intends to pay the relevant APC), the terms of reuse of such Open Access shall be governed by a Creative Commons licence – details of these licences and which Creative Commons licence will apply to this Work are set out in our licence referred to above.

Other than as permitted in any relevant BMJ Author's Self Archiving Policies, I confirm this Work has not been accepted for publication elsewhere, is not being considered for publication elsewhere and does not duplicate material already published. I confirm all authors consent to publication of this Work and authorise the granting of this licence.

For peer review only

How has the COVID-19 pandemic affected the utilization of online consultation and face-to-face medical treatment? An interrupted time-series study in Beijing, China.

[1] The Seventh Affiliated Hospital, Sun Yat-sen University, Guangdong, China

[2] School of Public Health, Capital Medical University, Beijing, China

First Author: Shan Zhang[1], Master's Degree, zsunll2017@163.com

Corresponding Author: Chengyu Ma[2], Associate Professor, 10132415@qq.com

Abstract

Objective The COVID-19 pandemic has had a major impact on healthcare utilization. This study aimed to quantify how the online and face-to-face utilization of healthcare services changed during this time and thus gain insights into the planning of future healthcare resources during the outbreak of infectious diseases.

Design This work is an interrupted time-series study.

Setting Monthly hospital-grade healthcare-service data from 22 tertiary first-class public hospitals managed by the Beijing Hospital Authority and online-consultation data from GoodDoctor were used in this study.

Methods This is an interrupted time-series study about the change in face-to-face and online healthcare utilization before and after the COVID-19 outbreak. We compared the impact of COVID-19 on the primary outcomes of both face-to-face healthcare utilization (outpatient and emergency visits, discharge volume) and online health care utilization (online consultation volume). And we also analysed the impact of COVID-19 on the healthcare utilization of different types of diseases.

Results The monthly average outpatient visits and discharges decreased by 36.33% and 35.75%, respectively, compared with those in 2019 in 22 public hospitals in Beijing. Moreover, the monthly average online consultations increased by 90.06%. A highly significant reduction occurred in the mean outpatients and inpatients, which dropped by 1,755,930 cases (P<0.01) and 5,920,000 cases (P<0.01), respectively. Online consultations rose by 3,650 cases (P<0.05). We identified an immediate and significant drop in healthcare services for four major diseases, i.e., acute myocardial infarction(AMI) (-174, P<0.1), lung cancer(LC) (-2,502, P<0.01), disk disease (-3,756, P<0.01), and Parkinson's disease (-205, P<0.01). Otherwise, online consultations for disk disease (63, P<0.01) and Parkinson's disease (25, P<0.05) significantly increased. More than 1,300 unique physicians provided online-consultation services per month in 2020, which was 35.3% higher than in 2019.

For peer review only

Conclusions Obvious complementary trends in online and face-to-face healthcare services existed during the COVID-19 pandemic. Different changes in healthcare utilization were shown for different diseases. Non-critically ill patients chose online consultation immediately after the COVID-19 lockdown, but critically ill patients chose hospital healthcare services first. Additionally, the volume of online physician services significantly rose as a result of COVID-19.

Keywords COVID-19, healthcare utilization, online consultation, health-service research

Strengths and limitations of this study

- Based on the Beijing Hospitals Authority and the GoodDoctor data, this work is an interrupted time-series study to quantify how the online and face-to-face utilization of healthcare services changed during the COVID-19 pandemic.

- The methodology in exploring the characteristics and trends of changes in healthcare utilization in the short and long term during the pandemic is noteworthy.

- ITS methodologies such as this have limitations in external environmental factors. Other policies and seasonal confounding factors such as meteorological factors may affect medical visits.

Introduction

In early 2020, the COVID-19 pandemic affected most of the planet and rapidly changed the utilization of healthcare services worldwide [1]. Fear of infection and reduced availability of healthcare services has led to decreased non-COVID healthcare utilization in hospitals [2,3]. The rapid and innovative deployment of telemedicine and online health services has occurred in many countries to relieve the pressure on hospitals and to prevent cross-infection [4,5]. In the United States, emergency room visits dropped by 42% in April, followed by a 26% reduction at the end of May, compared with 2019[6]. Meanwhile, telemedicine visits increased from 102.4 daily to 801.6 daily between March 2nd and April 14th 2020[7].

The impact of the global pandemic on healthcare systems is acute. As the COVID-19 pandemic continues, many studies have reported major changes in the utilization of healthcare services because of measures such as lockdowns and stay-at-home orders. These changes include large reductions in hospital visits, and some selective increases in online health services [8-10]. However, minimal data are available on the relationship between hospital visits and online health services over the same period, especially with quantitative time-trend evidence. Examining the short-term

For peer review only

and long-term patterns of changes in hospital volume and online health services during the COVID-19 pandemic is valuable. The extent and effects of substitution with online healthcare services for different kinds of diseases also require further investigation.

Beijing is a megacity with the most extensive internet accessibility (77.8%) [11] and the most famous hospitals compared with other parts of China. After the COVID-19 pandemic, many patients were unable to access hospitals in Beijing owing to the lockdown policies. In response, online health services and an internet-based health system have been utilized by hospitals in addition to third-party service platforms, such as GoodDoctor (www.haodf.com). Physicians are active in spending their free time on these platforms to provide patients with high-quality online health consultations. Online consultations refer to patients using the Internet to communicate with physicians in real-time through online text, pictures, video, audio, etc.

Figure 1 depicts the epidemic trends and significant changes in the COVID-19 outbreak in Beijing. The first confirmed case of COVID-19 in Beijing occurred on January 20, 2020. Since then, the virus has spread across Beijing. Beijing launched the first-level response mechanism for public health emergencies on January 24, 2020. To respond to public health emergencies promptly, China has launched a tiered response mechanism based on different pandemic levels and hazards. The first-level response mechanism is unified deployment by provincial headquarters according to the decisions of The State Council. The second-level response mechanism refers to deployment by the provincial headquarters. The third-level response mechanism refers to deployment by the headquarters of the prefect-level city. Two peaks occurred on February 2 and March 23 in the early days. Afterwards, the epidemic entered a normalization stage, with no new cases for a long time. On June 11, a 52-year-old man from Xinfadi Market in Beijing was diagnosed with COVID-19. After a 56-day zero-new-case interval, marking the beginning of "the second epidemic," new cases increased sharply on June 13. The number of new cases in a single day reached the highest value of 36 cases. Beijing's response mechanism for public-health emergencies was adjusted from level 3 to level 2 on June 16. This phenomenon led to a fast downward slope with the control of the Chinese government. The epidemic in Beijing has gradually stabilized since then. As of December 31, 987 confirmed cases and 9 deaths had been reported. In the present study, the change in short-term refers to the instantaneous change in medical-service utilization at the beginning of the pandemic from the end of December 2019 to February 2020, whereas long-term analysis refers to a snapshot change within the first year of the pandemic.

[Insert Figure 1 here]

This study was designed to compare and analyze the changing trend of online consultation and face-to-face healthcare volume, as well as the change in different disease types during the pandemic. The research illustrates the speed, scale, and reception of online-consultation utilization by patients and physicians, as well as the complementary trends in online and face-to-

For peer review only

face health services. The results may help provide insights into the planning of future healthcare resources during the outbreak of infectious diseases.

Methods

Study Design

We conducted an interrupted time-series analysis to estimate changes in face-to-face and online healthcare utilization before and after the COVID-19 outbreak. Simple pre-post designs perform before–after comparisons by estimating the change from a single pre-intervention time point to a single post-intervention time point. However, these have poor internal validity as they cannot exclude underlying trends as a cause for any change [12]. Conversely, interrupted time series (ITS) use multiple observations in the pre-intervention and post-intervention periods, thus offer thereby offering a quasi-experimental research design with a potentially high degree of internal validity [13,14]. We treated COVID-19 as the intervention, and 2019 and 2020 as the pre-intervention and post-intervention periods respectively, to analyze the change in face-to-face and online healthcare utilization after the COVID-19 outbreak.

Setting

Monthly hospital-grade healthcare-service data from January 2019 to December 2020 were provided by Beijing Hospitals Authority. Beijing municipal hospitals refer to the public hospitals managed by the Beijing Hospitals Authority of Beijing Municipal Health Commission.

Participants

A total of 22 hospitals were selected in this study, and they covered all tertiary first-class public hospitals managed by the Beijing Hospital Authority. Thirteen were general hospitals and nine were specialized hospitals (obstetrics and gynecology, pediatrics, oncology, stomatology, chest, psychiatry, traditional Chinese medicine, etc.). We collected outpatient and emergency visits, the number of discharges, online consultation volume from all 22 Beijing municipal hospitals, and patients including adults and children. Discharged patients refer to patients who are transferred from outpatient or emergency departments to inpatient departments, including acute, chronic, and severe cases.

Patient and Public Involvement

For peer review only

This work was an observational cohort study using publicly available data from the Beijing Hospital Authority and GoodDoctor website. Patients were not involved in our design, conduct, or reporting.

Data sources

First, outpatient and emergency visits, as well as the number of discharges in 2019 and 2020 from all 22 Beijing municipal hospitals were used as the overall face-to-face healthcare utilization outcomes. Four diseases including acute myocardial infarction (AMI), lung cancer (LC), disk disease, and Parkinson's disease were selected to analyze changes in healthcare-service utilization for different disease severities. These four diseases belonged to the 20 monitored diseases of the Beijing Municipal Health Commission. We used The International Statistical Classification of Diseases and Related Health Problems, Tenth Revision (ICD-10) to identify the principal diagnoses based on the main diagnosis of the electronic medical-record system of hospitals. Owing to data-sharing restrictions, the monthly number of discharges for these four diseases was collected from July 2019 to December 2020.

Second, online consultation data were obtained from GoodDoctor (www.haodf.com). GoodDoctor is the largest and longest-running online physician–patient communication platform in China [15]. It was founded in 2006. As of October 2021, it has collected information on 860 000 physicians from 9,780 regular hospitals in China, among which more than 73% are physicians in tertiary hospitals [16]. The 22 hospitals are all included in the GoodDoctor platform, covering more than 80% of outpatient doctors of these hospitals. A large number of patients turned to this platform for help after the COVID-19 pandemic outbreak, largely representing the trend in the utilization of online consultations during the pandemic.

The platform provides historical data of online consultations from 2010 to the present. We collected the daily online consultation data of the abovementioned 22 public hospitals in 2019 and 2020 and screened out the online-consultation volume data for AMI, LC, disk disease, and Parkinson's disease based on disease information. Figure 2 shows the content of the online consultation provided by this platform. We used Java language to design a web crawler and capture daily online-consultation information from web pages. Specific extracted fields include the date, website link, title, disease description, disease name, expected treatment, the duration of illness, consultation text of the patient and physician, and the name, title, department, patient voting numbers, online-consultation volume, and satisfaction of online service physicians. The final sample size collected was 70,086 for 2019 and 144,651 for 2020.

[Insert Figure 2 here]

Statistical methods

We fitted an ordinary least squares model by using either regress or Newey (with lag specified) with monthly service volume as the primary outcome of interest. We produced Newey–West standard errors to handle autocorrelation. We treated COVID-19 as the intervention, and 2019 and 2020 as the pre-intervention and post-intervention periods, respectively. The ITSA regression model is expressed as follows:

$$Y_t = \beta_0 + \beta_1 T_t + \beta_2 X_t + \beta_3 X_t T_t + \varepsilon_t$$

where Y_t is the effect variable, including outpatient and emergency visits, discharge volume, and online-consultation volume; T_t is the time since the start of the study, X_t is a dummy variable representing the intervention (pre-intervention period 0, otherwise 1), and $X_t T_t$ is the interaction term; β_0 is the intercept or starting level of the outcome variable; β1 is the slope or trajectory of the outcome variable until the COVID-19 outbreak; β_2 is the change in the level of outcome that occurred in the period immediately following the COVID-19 outbreak (compared with the counterfactual); and β_3 is the difference between the pre-pandemic and pandemic slopes of the outcome. Accordingly, we searched for significant p-values in β_2 to indicate an immediate treatment effect or in β_3 to indicate a treatment effect over time.

Moreover, we determined the impact of COVID-19 on the utilization of healthcare services for different diseases.

We stratified models by disease type restricted to (1) AMI, (2) LC, (3) disk disease, and (4) Parkinson’s disease. They were included in the 20 monitored diseases of Beijing Hospital Authority with clear diagnostic criteria. These four diseases had a certain number of patients online and face-to-face. AMI and LC were considered severe cases, whereas disk disease and Parkinson's disease were considered mild cases in the research. All statistical analyses were conducted using Stata 15.0 and SPSS 20.0.

Bias

Although interrupted time series has high internal validity, external confounding factors cannot be controlled. Since the outbreak of COVID-19 across the entirety of China in 2020, we cannot find an effective control group. A certain confounding bias existed in this study.

Result

1. **Impact of the COVID-19 pandemic on the utilization of healthcare services**

For peer review only

Table 1 shows the descriptive statistical indicators of the face-to-face outpatient and emergency visits, discharges, and online consultations in 2019 and 2020. Compared with 2019, the utilization of face-to-face healthcare services decreased in 2020. The monthly average outpatient and emergency visits, as well as discharges, decreased by 36.33% and 35.75%, respectively. Compared with 2019, the utilization of online consultations increased in 2020. The monthly average online consultations increased by 90.06%.

Table 1 Healthcare-service utilization in 2020 compared with 2019 (ten thousand)

	2019 (before the COVID-19 outbreak)			2020 (after the COVID-19 outbreak)		
	face-to-face outpatient and emergency visits	face-to-face discharges	online consultation	face-to-face outpatient and emergency visits	face-to-face discharges	online consultation
Mean	270.179	8.677	0.634	172.018	5.575	1.205
Std.	25.779	0.918	0.054	54.554	2.160	0.238
Median	274.391	8.956	0.611	179.315	5.996	1.272
Max	311.549	9.559	0.754	243.728	8.292	1.450

Figure 3 shows the percentage changes in healthcare-service utilization in Beijing. Compared with the same period in 2019, we observed lower monthly face-to-face outpatient and emergency visits, as well as discharges in 2020. The percentage change frequency for both was the greatest in March, with 66.97% and 75.17% decrease, respectively. The overall change decreases thereafter. Notably, when affected by "the second epidemic," face-to-face healthcare services increased slightly again, but utilization in July fell at a higher rate than in June. Online consultations in all months except January were higher than in the same period in 2019. The overall change increased. The greatest decrease was observed in August. Figure 3 shows that in the early days of the pandemic (January to June), the utilization of online and face-to-face healthcare services showed obvious complementary trends. Afterwards, the growth rate of online consultations remained relatively high, whereas the decline rate of face-to-face healthcare services gradually decreased. This finding suggested that the utilization of face-to-face healthcare services is gradually returning to normal levels as COVID-19 fades away, whereas online consultations is maintaining a high growth rate.

[Insert Figure 3 here]

For peer review only

2. Interrupted time-series analysis

Changes in overall online and face-to-face healthcare services

Table 2 shows a highly significant reduction in the mean number of outpatient and emergency visits, as well as discharges, which dropped by 1,755,930 cases (P<0.01) and 59,200 cases (P<0.01), respectively. Before the COVID-19 outbreak, face-to-face healthcare services (including outpatient and emergency visits and discharges) increased (Figure 4). Soon after the COVID-19 outbreak, the slope of this trend line significantly increased, but the trend was not statistically significant. Online consultations also rose by 3,650 cases (P<0.05), followed by a significant increase in the monthly trend of utilization (relative to the pre-pandemic) of 580 cases.

Table 2 Impact of COVID-19 on the utilization of online and face-to-face healthcare services (ten thousand)

	Medical utilization reduction after the COVID-19 outbreak (intercept)	P value	Average monthly medical utilization decline after the COVID-19 outbreak (potential trend)	P value
face-to-face outpatient and emergency visits	-175.593 (27.247*)	<0.01	7.357 (4.454)	>0.1
face-to-face discharges	-5.920 (1.608)	<0.01	0.283 (0.217)	>0.1
online consultation	0.365 (0.143)	<0.05	0.058 (0.019)	<0.01

Standard error in parentheses.

* p<0.1, **p<0.05, ***p<0.01.

[Insert Figure 4 here]

Changes in face-to-face healthcare services by type of disease

Table 3 summarizes the effect of COVID-19 on face-to-face healthcare services for different diseases. The COVID-19 outbreak was associated with a rapid decline in face-to-face healthcare services for different diseases. From the perspective of immediate effect, we identified a significant drop in healthcare services for the four diseases. From the perspective of long-term effects, AMI, LC, and disk disease services showed a nonsignificant stepwise increase (the slope of the trend line increased). Simultaneously, the monthly trend of utilization for Parkinson's disease

For peer review only

significantly increased (relative to the pre-pandemic period) (Figure 5).

Table 3 Impact of COVID-19 on the utilization of face-to-face healthcare services and online consultation by type of disease

	Medical utilization reduction after the COVID-19 outbreak (intercept)		Average monthly medical utilization decline after the COVID-19 outbreak (potential trend)	
	face-to-face healthcare services	online consultation	face-to-face healthcare services	online consultation
acute myocardial infarction	-174.405 (84.060) *	-5.998 (3.511)	12.438 (11.540)	1.633 (0.472) ***
lung cancer	-2,502.02 (367.118) ***	-11.832 (19.413)	80.508 (80.480)	14.360 (3.079) ***
disk disease	-375.560 (72.369) ***	63.118 (11.955) ***	29.163 (16.876)	3.402 (1.851) *
Parkinson’s disease	-205.229 (47.852) ***	24.625 (10.349) **	12.669 (4.892) **	1.772 (1.335)

Standard error in parentheses.

* p<0.1, **p<0.05, ***p<0.01.

[Insert Figure 5 here]

Changes in online consultations by type of disease

Table 3 summarizes the effect of COVID-19 on online consultations for different diseases. The COVID-19 outbreak was associated with a rapid rise in online consultations for different diseases. From the perspective of immediate effect, we identified a significant increase in healthcare services for disk disease and Parkinson’s disease. Conversely, online consultations for AMI and LC showed a small, nonsignificant decrease. From the perspective of long-term effects, online consultations for AMI, LC, and disk disease increased monthly at a rate of 1,633, 14,360, and 3,402, respectively after the COVID-19 outbreak (Figure 6).

[Insert Figure 6 here]

3. **Impact of COVID-19 pandemic on online-service physicians’ volume**

As affected by COVID-19, the monthly volume of online services provided by physicians in 2020 was significantly higher than that in 2019, except for January. On average, more than 1,300

For peer review only

unique physicians from 22 hospitals in Beijing provided online-consultation services per month in 2020, which was 35.3% higher than that in 2019. This finding suggested that the supply of online consultations greatly increased after the COVID-19 outbreak (Figure 7).

[Insert Figure 7 here]

Discussion

Based on monthly treatment data from 22 public hospitals in Beijing, obvious complementary trends existed in online and face-to-face healthcare services during the COVID-19 pandemic. We found that public hospitals in Beijing experienced substantial losses in face-to-face healthcare and substantial increases in online-consultation services when the COVID-19 pandemic began in China in 2020. The interrupted time-series models revealed that after the COVID-19 outbreak, the considerable reduction in face-to-face healthcare services was a short-term effect, whereas the increase in online consultation was a long-term one. As only one year of the pandemic was examined in the current study, the long-term effects herein referred to change trends within one year. In other words, this study is a snapshot. Simultaneously, the effects of COVID-19 on healthcare services for different diseases varied. Noncritical-disease patients showed greater reductions in face-to-face service utilization and chose online consultations immediately after the COVID-19 lockdown. However, critical-disease patients chose face-to-face treatment first, and slowly moved online over time. The volume of online services provided by physicians also significantly increased as a result of COVID-19.

Our study is consistent with previous reports on hospital healthcare-service changes during the COVID-19 pandemic [17,18]. A systematic review from the UK analyzed 81 studies and reported that the utilization of healthcare in care facilities sharply decreased by about a third during the pandemic period, with greater reductions among people with less severe illnesses [17]. Additionally, according to a study from one large health system (NYU Langone Health) in the United States, telemedicine visits increased from 102.4 daily to 801.6 daily between March 2 and April 14 2020, with a concomitant decline in in-person visits by over 80%. We also showed that online consultation underwent sustained growth owing to the impact of COVID-19, whereas face-to-face healthcare services showed a short-term decline. An important reason for the decline in face-to-face visits was that rigorous public health measures were implemented in China during the COVID-19 epidemic [19]. Some medical resources responded to the outbreak. The number of outpatient and emergency visits to 22 municipal hospitals in Beijing decreased by 36.33% in 2020. More medical personnel provided epidemic prevention and control services, such as nucleic acid tests and vaccines. Furthermore, potential risk factors in the hospital environment made people anxious and worried. A survey has shown that 20% of pregnant women fear going to a hospital for consultation, and more than 40% fear going to a hospital for check-ups [20].

A rapid decrease in overall face-to-face discharge admissions was observed for four different diseases across 22 tertiary public hospitals in Beijing after the COVID-19 lockdown. A study performed in Germany revealed a similar reduction in traditional face-to-face healthcare services for different diseases [21]. That study found that hospital admissions for malignant cancer treatments and AMI decreased after the lockdown announcement. However, a difference was that our analysis also showed the impact of the epidemic on diseases with varied severity. We also examined the changes in online consultations for different diseases. We found that different diseases showed different online change trends. Online consultations for critical illnesses (such as AMI, and LC) did not increase immediately after the outbreak but gradually increased in the long-term study. A study has shown that telemedicine can be used to reach patients with severe disease burdens [22]. Conversely, we observed that noncritical clinical situations (such as disk disease and Parkinson's disease) considerably and immediately increased, and this trend has been maintained.

Several reasons may explain the dramatic decrease in face-to-face healthcare services after the COVID-19 pandemic. First, lockdown policy and population mobility control measures were implemented in China to mitigate the spread of the pandemic. For instance, the community implemented closed management, hospital services were limited, and non-emergency services had to be reserved in advance and staggered used in Beijing during the pandemic period. This situation may have reduced the availability and accessibility of face-to-face healthcare services. Second, some patients chose to postpone their treatment to prevent cross-infection in the hospital. Most healthcare resources were relocated to manage the pandemic. This setup may trigger attitudes toward delaying less urgent cases by the patient and the healthcare system. Third, some health demands may have been eliminated. For instance, many patients with self-resolving diseases chose not to avail of face-to-face treatment, such as for colds, diarrhea, etc. [23,24]. The more important point was that some health demands may have been transferred online. Abundant internet diagnosis and treatment services were supplied by medical institutions in Beijing during the pandemic, such as online consultation, instant messaging, synchronous chat, video conferencing, etc. Online consultation can span time and space. The physician and patient do not have to meet each other within a physical space, subsequently decreasing the requirement for face-to-face travel, appointment-related concerns, and the risk of face-to-face infection. Some patients could migrate to online consultation to meet their health needs, which can explain the significantly increased volume of online consultations.

On one hand, our results indicated that mass migration to online consultations occurred shortly after the COVID-19 pandemic. On the other hand, the migration of critically ill patients was slower than that of noncritically ill ones because the latter relied more on facility treatment. For example, the efficiency of the care pathway at every step matters very much for AMI patients [25]. Chemotherapy, radiation therapy, surgery, and molecular targeted therapy are keystones in the treatment of early and locally advanced LC with good prognosis [26]. These conditions rely on face-

For peer review only

to-face operations and inspections, and online consultations cannot provide these services, which is why the online-consultation volume for critical illnesses did not increase immediately after the outbreak. Additionally, we found that the online-consultation volume for three diseases, except for Parkinson's, continuously increased. This phenomenon can be explained by the fact that the COVID-19 pandemic accelerated the popularity of online healthcare, thereby increasing the awareness and acceptance of the public regarding online healthcare.

Our study also found that the pandemic led to a significant increase in online patients and also facilitated the online migration of physicians. The number of physicians offering services online in 2020 was much higher than that over the same period in 2019, except for January. This finding showed that physicians paid attention to providing online healthcare services.

With the COVID-19 pandemic, the supply of face-to-face healthcare services was relatively insufficient, resulting in partial healthcare-service disruption. The COVID-19 pandemic resulted in decreased supply of face-to-face healthcare services, which was part of the healthcare-service disruption. To reduce the public-health impact, we put forward two suggestions: first, we should designate some part of healthcare resources to online provision. Moreover, we should rapidly establish a collaborative model of face-to-face and online healthcare services in public-health emergencies to meet the demand of patients. Online consultations and telemedicine can alleviate geographic and physician shortages, expand healthcare accessibility, and improve the quality of healthcare provision. Therefore, online healthcare services can serve as an important supplement to traditional healthcare services, optimize health resource allocation, and improve service efficiency, especially for noncritically ill patients.

However, not all diseases are suitable for online consultations. Face-to-face healthcare services are necessary and irreplaceable, such as physical examinations, diagnoses, and treatment of difficult and severe diseases. Different healthcare support measures should be formulated according to the characteristics of different diseases. For critical cases, face-to-face channels ought to be opened for diagnosis and treatment to ensure that patients can receive face-to-face treatment in a timely manner. Studies indicate that 9.1% of patients experienced a delay in LC treatment, and half of AMI patients did not reach out to the hospital at all during the COVID-19 pandemic [25,27]. For critical cases, delaying or stopping treatment can have a significant negative impact on the physical and mental health of critically ill patients. A timely diagnosis and improved treatment strategy will facilitate the health of patients [26]. For mild cases, cooperation among the government, health facilities, and third-party platforms should be enhanced. Online healthcare services such as telemedicine, online consultation, psychological counseling, and chronic disease revisits ought to be expanded. Expanding the supply of online healthcare resources and helping patients migrate online to receive healthcare services are crucial. Meanwhile, a comparison between the quality of online consultation and face-to-face care is worth discussing. An in-depth review shows that online consultation is a useful, high-quality, and safe option for the vast majority

For peer review only

of pediatric neurology patients [28]. A study has shown that no significant difference exists between the satisfaction of online counseling and face-to-face healthcare [29]. However, studies have also shown that people still worry about the reliability of online healthcare [30].

Finally, our work confirmed that the increase in online consultations was a persistent phenomenon during the research period. A new normal deployment of online healthcare services occurred in the pandemic and policy-enabling environment. The development of online consultation should be encouraged because of the advantages of online consultation across time and space. New technologies such as 5G technology, sensing techniques, and artificial intelligence are bound to be fully exploited for online consultation. Innovating the long-term synergistic modalities of online and face-to-face healthcare services is important to promote the connection among high-quality medical resources at all levels.

This study had some limitations. First, we studied the complementary trend of online and face-to-face health service utilization. Whether the change in service delivery caused an impact on the quality or the equivalency of care delivery requires further study. Second, to observe the changes in healthcare-service utilization, the behavior of patients with diseases of varied severity, i.e., AMI, LC, disk disease, and Parkinson's disease, was selected in this paper. Further studies are needed to determine whether this phenomenon can be extended to other diseases. The heterogeneity of the data across the hospitals was also not explored. Third, the usage of GoodDoctor cannot cover all the online-consultation behavior. Although GoodDoctor is the largest online consultant platform in China, some other online-consultant platforms are available in the industry. We choose the GoodDoctor platform by considering that the 22 surveyed hospitals all had a certain number of doctors in the platform, which was representative to a certain extent. Finally, this study was only a snapshot capturing the first year of the pandemic response. Comprehensive research over a longer period is required.

Conclusion

Face-to-face healthcare services dropped precipitously and online consultations rose sharply in 22 tertiary public hospitals in Beijing during the COVID-19 pandemic. However, face-to-face healthcare services showed a relatively fast recovery, and online consultations continued to grow. The changing trend in online-consultation volume varied by disease, which was characterized by a nonsignificant increase in the short term and a significant increase in the long term regarding critical cases after the outbreak, as well as a significant increase in noncritical cases in the long short term. The availability of online consultations should be encouraged during pandemics. Particular attention should be given to severely ill patients who need adequate and prompt face-to-face hospital care.

For peer review only

Author affiliations

1 The Seventh Affiliated Hospital, Sun Yat-sen University, Guangdong, China

2 School of Public Health, Capital Medical University, Beijing, China

Contributors The research idea originated with CM, but all authors designed the study. SZ directed the data analyses. CM and SZ co-wrote this manuscript.

Funding This study has been funded by the Natural Science Foundation of Beijing Municipality (grant number: 9222993).

Competing interests We have read and understood the BMJ policy on the declaration of interests. All authors report no competing interests.

Patient and public involvement statement Patients and the public were not involved in the design or conduct of this study. The study used publicly available data from GoodDoctor, which has signed privacy policies with users.

Patient consent for publication Not applicable.

Ethics approval It was approved by the ethics committee of Capital Medical University (approval No. Z2020SY126).

Provenance and peer review Not commissioned; externally peer-reviewed.

Data availability statement Data are available on reasonable request.

For peer review only

Abbreviations

AMI: Acute Myocardial Infarction

LC: Lung Cancer

References

1 Moynihan R, Sanders S, Michaleff Z A, et al. Impact of COVID-19 pandemic on utilisation of healthcare services: a systematic review[J]. BMJ Open, 2021, 11(3): e045343.

2 Xu S, Glenn S, Sy L, et al. Impact of the COVID-19 Pandemic on Health Care Utilization in a Large Integrated Health Care System: Retrospective Cohort Study. J Med Internet Res. 2021 Apr 29;23(4): e26558.

3 Hartnett KP, Kite-Powell A, DeVies J, et al. National Syndromic Surveillance Program Community of Practice. Impact of the COVID-19 Pandemic on Emergency Department Visits - United States, January 1, 2019-May 30, 2020. MMWR Morb Mortal Wkly Rep. 2020 Jun 12;69(23):699-704.

4 Lukas H, Xu C, Yu Y, et al. Emerging Telemedicine Tools for Remote COVID-19 Diagnosis, Monitoring, and Management. ACS Nano. 2020 Dec 22;14(12):16180-16193.

5 Bokolo Anthony Jnr. Use of Telemedicine and Virtual Care for Remote Treatment in Response to COVID-19 Pandemic. J Med Syst. 2020 Jun 15;44(7):132.

6 Garcia S, Albaghdadi M S, Meraj P M, et al. Reduction in ST-Segment Elevation Cardiac Catheterization Laboratory Activations in the United States During COVID-19 Pandemic[J]. Journal of the American College of Cardiology, 2020.

7 Mann DM, Chen J, Chunara R, et al. COVID-19 transforms health care through telemedicine: Evidence from the field. J Am Med Inform Assoc. 2020 Jul 1;27(7):1132-1135.

8 Chen Y, Cai M, Li Z, et al. Impacts of the COVID-19 Pandemic on Public Hospitals of Different Levels: Six-Month Evidence from Shanghai, China. Risk Manag Healthc Policy. 2021;14: 3635-3651.

9 Li G, Fan G, Chen Y, et al. What patients "see" doctors in online fever clinics during COVID-19 in Wuhan? J Am Med Inform Assoc. 2020 Jul 1;27(7):1067-1071.

10 Shimels T. The Trend of Health Service Utilization and Challenges Faced During the COVID-19 Pandemic at Primary Units in Addis Ababa: A Mixed-Methods Study. Health Serv Res Manag Epidemiol. 2021 Jul 8;8:23333928211031119.

11 China Internet Network Information center. Beijing, China: CNNIC, 2021. Annual Report 2021.

For peer review only

12 James, Lopez, Bernal, et al. The use of controls in interrupted time series studies of public health interventions.[J]. International Journal of Epidemiology, 2018.

13 Shadish W, Cook T, Campbell D. Experimental and Quasi-Experimental Designs For Generalized Causal Inference. Houghton Mifflin, 2002.

14 Miller CJ, Smith SN, Pugatch M. Experimental and quasi-experimental designs in implementation research. Psychiatry Res. 2020 Jan;283:112452.

15 Liu X, Chen M, Li J, et al. How to Manage Diversity and Enhance Team Performance: Evidence from Online Doctor Teams in China. Int J Environ Res Public Health. 2019 Dec 19;17(1):48.

16 The GoodDoctor profile, The GoodDoctor. 2022. http://www.haodf.com/info/aboutus.php

17 Moynihan R, Sanders S, Michaleff ZA, et al. Impact of COVID-19 pandemic on utilisation of healthcare services: a systematic review. BMJ Open. 2021 Mar 16;11(3): e045343.

18 Mann DM, Chen J, Chunara R, et al. COVID-19 transforms health care through telemedicine: Evidence from the field. J Am Med Inform Assoc. 2020 Jul 1;27(7):1132-1135.

19 Yang ZM, Wu MY, Lu JM, et al. Effect of COVID-19 on hospital visits in Ningbo, China: an interrupted time-series analysis. Int J Qual Health Care. 2021 May 7;33(2): mzab078.

20 Wu H, Sun W, Huang X, et al. Online Antenatal Care During the COVID-19 Pandemic: Opportunities and Challenges. J Med Internet Res. 2020 Jul 22;22(7): e19916

21 Kapsner LA, Kampf MO, Seuchter SA, et al. Reduced Rate of Inpatient Hospital Admissions in 14 German University Hospitals During the COVID-19 Lockdown. Front Public Health. 2021 Jan 13;8: 594117.

22 Shehan JN, Agarwal P, Danis DO 3rd, et al. Effects of COVID-19 on telemedicine practice patterns in outpatient otolaryngology. Am J Otolaryngol. 2021 Nov-Dec;42(6):103044.

23 Giannouchos TV, Biskupiak J, Moss MJ, et al. Trends in outpatient emergency department visits during the COVID-19 pandemic at a large, urban, academic hospital system. Am J Emerg Med. 2021 Feb;40:20-26.

24 Meng H, Ji LN, Huang J, et al. Analysis of the changes and characteristics of pediatric outpatient visits in a general hospital in Beijing before and after the COVID-19 pandemic. Beijing Da Xue Xue Bao Yi Xue Ban. 2021 Oct 18;53(5):952-956. Chinese.

25 German University Hospitals During the COVID-19 Lockdown. Front Public Health. 2021 Jan 13;8: 594117.

26 De Rosa S, Spaccarotella C, Basso C, et al. Reduction of hospitalizations for myocardial infarction

For peer review only

in Italy in the COVID-19 era. Eur Heart J. 2020 Jun 7;41(22):2083-2088.

27 Sha Z, Chang K, Mi J, Liang Z, et al. The impact of the COVID-19 pandemic on lung cancer patients. Ann Palliat Med. 2020 Sep;9(5):3373-3378.

28 Joshi CN, Yang ML, Eschbach K, et al. Quality and Safety Analysis of 2,999 Telemedicine Encounters During the COVID-19 Pandemic. Neurol Clin Pract. 2021 Apr;11(2): e73-e82.

29 Fieux M, Duret S, Bawazeer N, et al. Telemedicine for ENT: Effect on quality of care during Covid-19 pandemic. Eur Ann Otorhinolaryngol Head Neck Dis. 2020 Sep;137(4):257-261.

30 Wu H, Sun W, Huang X, et al. Online Antenatal Care During the COVID-19 Pandemic: Opportunities and Challenges. J Med Internet Res. 2020 Jul 22;22(7): e19916.

Figure legends

Figure 1 COVID-19 epidemic trend in Beijing

Figure 2 Information on online consultation provided by this platform

Figure 3 Percentage change in healthcare-service utilization in 2020 compared with that in 2019

Figure 4 Effect of COVID-19 intervention on face-to-face and online healthcare utilization. Scatter plots represent monthly medical utilization. The black line of best fit represents medical utilization across estimated by post-regression. The vertical black line is the intervention point. (a) Face-to-face outpatient and emergency visits, (b) face-to-face discharges, and (c) online consultation.

Figure 5 Effect of COVID-19 intervention on face-to-face healthcare utilization by type of disease. Scatter plots represent medical utilization monthly. The black line of best fit represents medical utilization across estimated by post-regression. The vertical black line is the intervention point. (a)AMI, (b) LC, (c) disk disease, and (d) Parkinson’s disease.

Figure 6 Effect of COVID-19 intervention on online-consultation utilization by disease type. Scatter plots represent monthly medical utilization. The black line of best fit represents medical utilization

For peer review only

across estimated by post-regression. The vertical black line is the intervention point. (a)AMI, (b) LC, (c) disk disease, and (d) Parkinson’s disease.

Figure 7 Changes in online physician volume in 2020 compared with that in 2019. The dotted line is the mean.

For peer review only

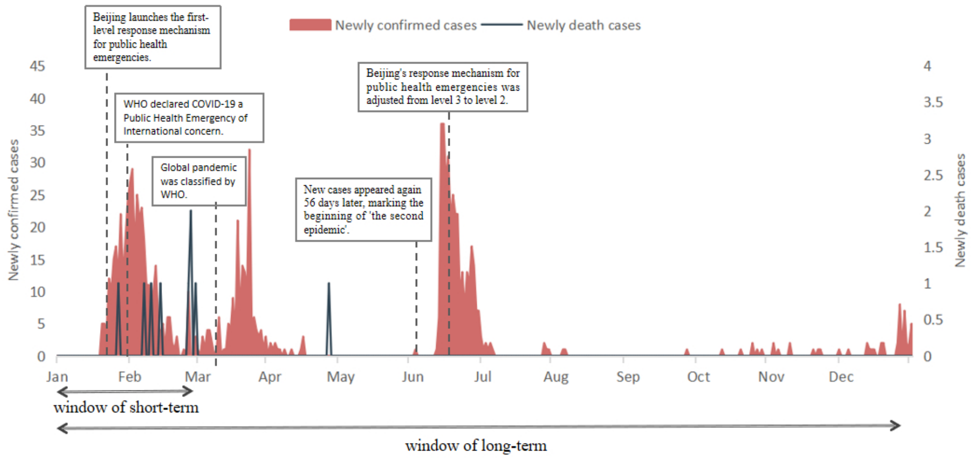

Figure 1 COVID-19 epidemic trend in Beijing

478x244mm (57 x 57 DPI)

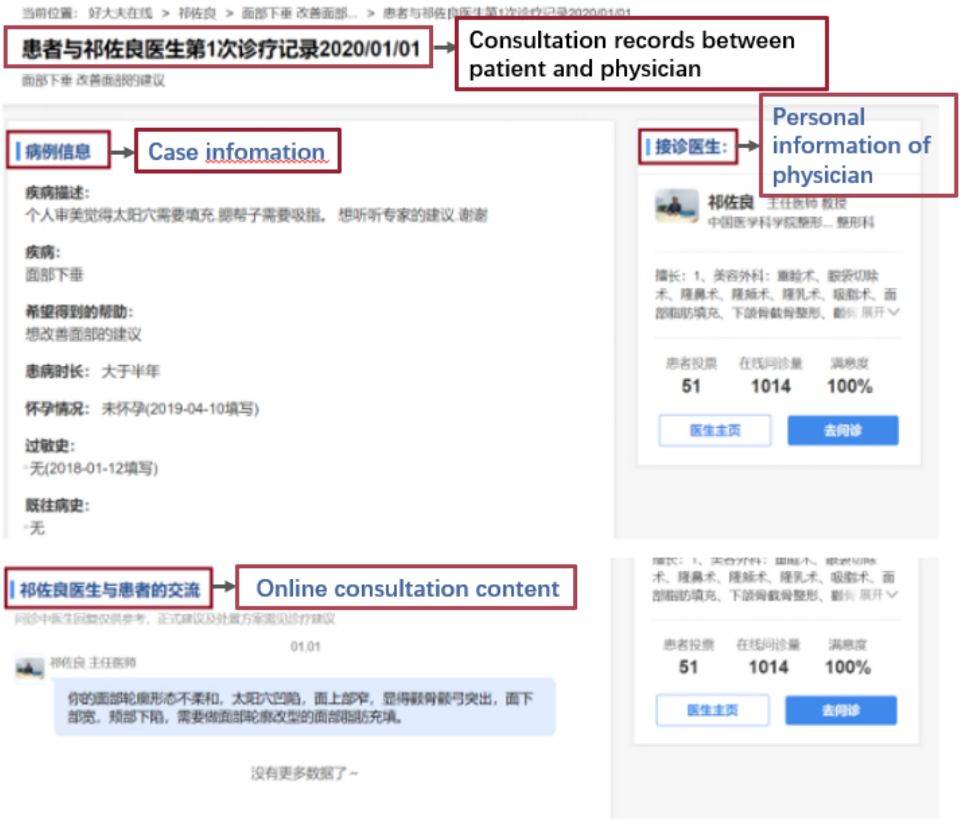

Figure 2 The information of the online consultation provided by GoodDoctor platform

331x283mm (57 x 57 DPI)

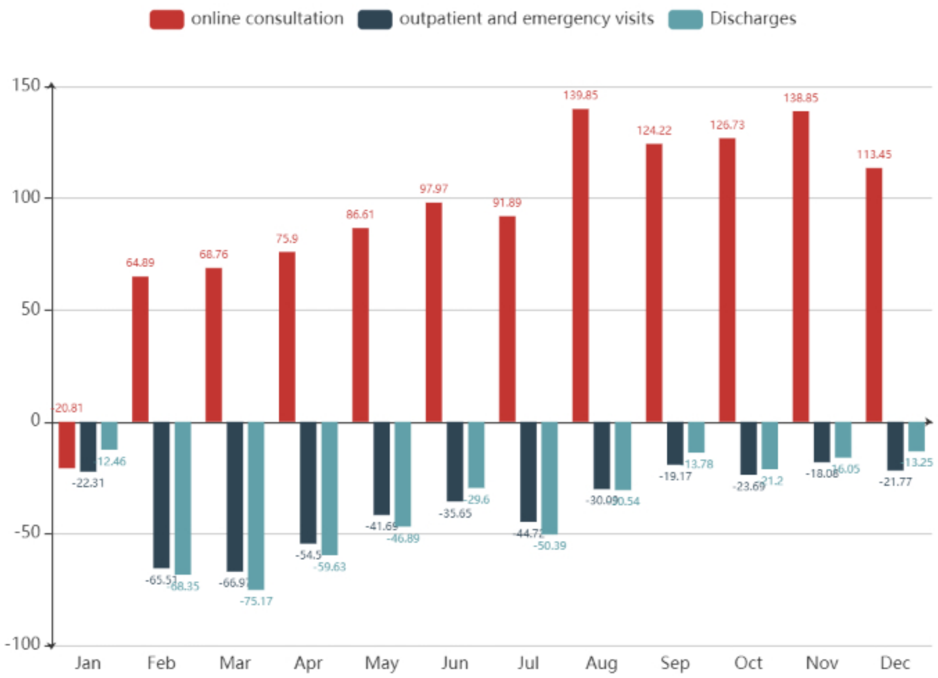

Figure 3 Percentage change in healthcare service utilization in 2020 compared with 2019 (%)

254x175mm (72 x 72 DPI)

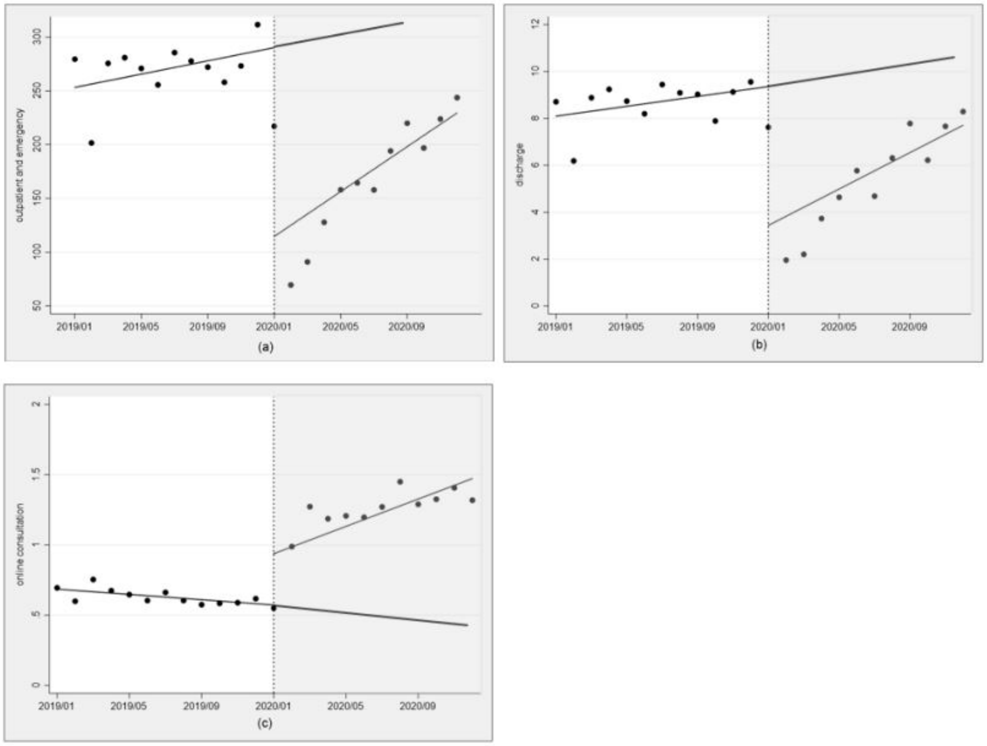

Figure 4 The effect of COVID-19 intervention on face-to-face and online healthcare utilization. Scatter plots represent monthly medical utilization. The black line of best fit represents medical utilization across estimated by post-regression. The vertical black line is the intervention point. (a) face-to-face outpatient and emergency visits; (b) face-to-face discharges; (c) online consultation.

143x108mm (144 x 144 DPI)

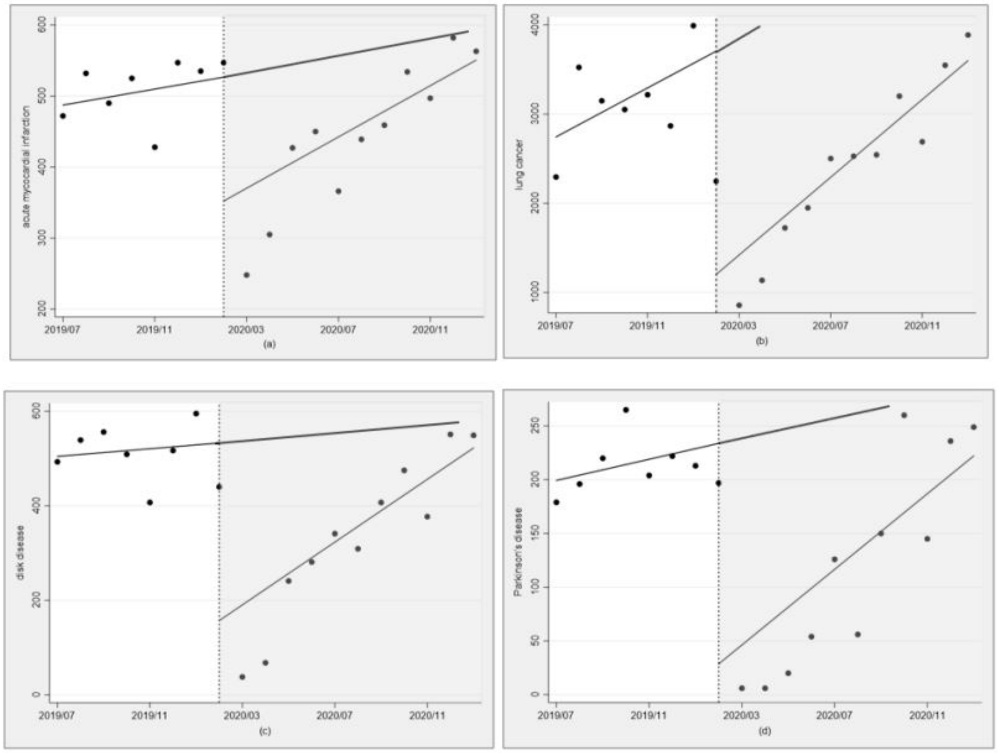

Figure 5 The effect of COVID-19 intervention on face-to-face healthcare utilization by type of disease. Scatter plots represent medical utilization monthly. The black line of best fit represents medical utilization across estimated by post-regression. The vertical black line is the intervention point. (a) AMI; (b)LC; (c) disk disease; (d) Parkinson's disease.

142x108mm (144 x 144 DPI)

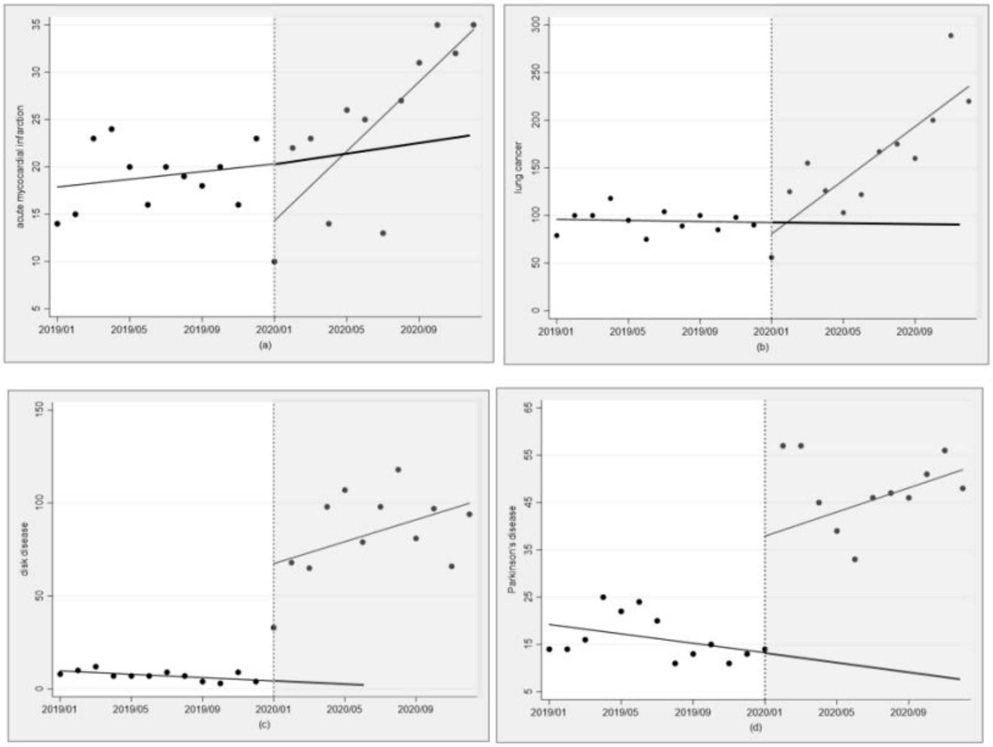

Figure 6 The effect of COVID-19 intervention on online consultations utilization by type of disease. Scatter plots represent medical utilization monthly. The black line of best fit represents medical utilization across estimated by post-regression. The vertical black line is the intervention point. (a)AMI; (b) LC; (c) disk disease; (d) Parkinson's disease.

144x108mm (144 x 144 DPI)

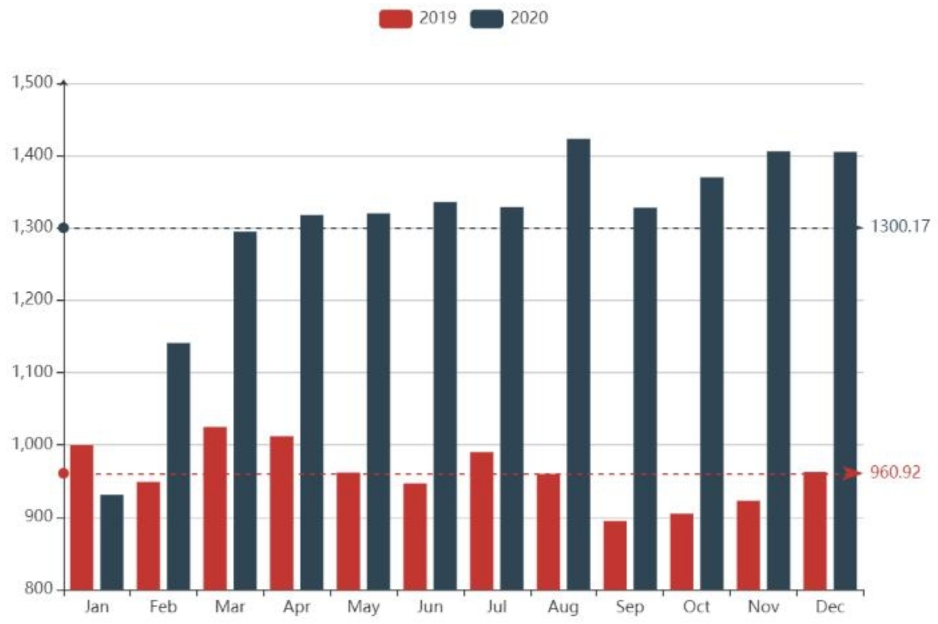

Figure 7 Changes in online physician volume in 2020 compared with 2019 (%). The dotted line is the mean.

131x88mm (144 x 144 DPI)

STROBE Statement—checklist of items that should be included in reports of observational studies

	Item No.	Recommendation	Page No.	Relevant text from manuscript
**Title and abstract**	1	(*a*) Indicate the study's design with a commonly used term in the title or the abstract	Page 1 line 1-2	COVID-19, interrupted time-series study
		(*b*) Provide in the abstract an informative and balanced summary of what was done and what was found	Page 1 line 9-32, Page 2 line 1-2	We report the main research contents, results and conclusions in the abstract. We found that the online and offline trends were complementary during the pandemic. Obvious complementary trends in online and face-to-face healthcare services existed during the COVID-19 pandemic through the time series analysis. And different changes in healthcare utilization were shown for different diseases.
**Introduction**				
Background/rationale	2	Explain the scientific background and rationale for the investigation being reported	Page 2 line 17-24, Page 3 line 2-30	In the context of the COVID-19 pandemic, fear of infection and reduced availability of healthcare services has led to decreased non-COVID healthcare utilization in hospitals. And online health services have occurred in many countries to relieve the pressure on hospitals and to prevent cross-infection. We described the pandemic situation in Beijing.
Objectives	3	State specific objectives, including any prespecified hypotheses	Page 3 line 17-24	This study was designed to compare and analyze the changing trend of online consultation and face-to-face healthcare volume, as well as the change in different disease types during the pandemic. The research illustrates the speed, scale, and reception of online-consultation utilization by patients and physicians, as

For peer review only - http://bmjopen.bmj.com/site/about/guidelines.xhtml

For peer review only

				well as the complementary trends in online and face-to-face health services.
**Methods**				
Study design	4	Present key elements of study design early in the paper	Page 4 line 5-14	We conducted an interrupted time-series analysis. We treated COVID-19 as the intervention, and 2019 and 2020 as the pre- and post-intervention periods respectively, to analyze the change in face-to-face and online healthcare utilization after the COVID-19 outbreak.
Setting	5	Describe the setting, locations, and relevant dates, including periods of recruitment, exposure, follow-up, and data collection	Page 4 line 17-19	Monthly hospital-grade healthcare-service data from January 2019 to December 2020 were provided by Beijing Hospitals Authority. Beijing municipal hospitals refer to the public hospitals managed by the Beijing Hospitals Authority of Beijing Municipal Health Commission.
Participants	6	(*a*) *Cohort study*—Give the eligibility criteria, and the sources and methods of selection of participants. Describe methods of follow-up<br><br>*Case-control study*—Give the eligibility criteria, and the sources and methods of case ascertainment and control selection. Give the rationale for the choice of cases and controls<br><br>*Cross-sectional study*—Give the eligibility criteria, and the sources and methods of selection of participants	Page 4 line 22-27	A total of 22 hospitals were selected in this study, and they covered all tertiary first-class public hospitals managed by the Beijing Hospital Authority. Thirteen were general hospitals and nine were specialized hospitals (obstetrics and gynecology, pediatrics, oncology, stomatology, chest, psychiatry, traditional Chinese medicine, etc.).
		(*b*) *Cohort study*—For matched studies, give matching criteria and number of exposed and unexposed		N/A

For peer review only - http://bmjopen.bmj.com/site/about/guidelines.xhtml

For peer review only

		Case-control study—For matched studies, give matching criteria and the number of controls per case		
Variables	7	Clearly define all outcomes, exposures, predictors, potential confounders, and effect modifiers. Give diagnostic criteria, if applicable	Page 6 line 1-5	We fitted an ordinary least squares model by using either regress or Newey (with lag specified) with monthly service volume as the primary outcome of interest. We produced Newey–West standard errors to handle autocorrelation. We treated COVID-19 as the intervention, and 2019 and 2020 as the pre-intervention and post-intervention periods, respectively.
Data sources/ measurement	8*	For each variable of interest, give sources of data and details of methods of assessment (measurement). Describe comparability of assessment methods if there is more than one group	Page 5 line 4-31	Outpatient and emergency visits, as well as the number of discharges in 2019 and 2020 from all 22 Beijing municipal hospitals, were used as the overall face-to-face healthcare utilization outcomes. And the monthly number of discharges for AMI, LC, disk disease, and Parkinson's disease four diseases were collected from July 2019 to December 2020. Online consultation data were obtained from GoodDoctor
Bias	9	Describe any efforts to address potential sources of bias	Page 6 line 26-28	Although interrupted time series has high internal validity, external confounding factors cannot be controlled. Since the outbreak of COVID-19 across the entirety of China in 2020, we cannot find an effective control group. A certain confounding bias existed in this study.
Study size	10	Explain how the study size was arrived at	Page 4 line 22-27	A total of 22 hospitals were selected in this study, and they covered all tertiary first-class public hospitals managed by the Beijing Hospital Authority. Thirteen were general hospitals and nine were specialized hospitals (obstetrics and gynecology, pediatrics, oncology, stomatology, chest, psychiatry, traditional Chinese medicine, etc.).We collected outpatient and emergency visits, the number of discharges, online consultation volume from

For peer review only - http://bmjopen.bmj.com/site/about/guidelines.xhtml

	all 22 Beijing municipal hospitals, and patients including adults and children.

Continued on next page

For peer review only

Quantitative variables	11	Explain how quantitative variables were handled in the analyses. If applicable, describe which groupings were chosen and why	Page 6 line 6-15	Y_t is the effect variable, including outpatient and emergency visits, discharge volume, and online-consultation volume; Tt is the time since the start of the study, X_t is a dummy variable representing the intervention (pre-intervention period 0, otherwise 1), and X_tT_t is the interaction term; β_0 is the intercept or starting level of the outcome variable; β_1 is the slope or trajectory of the outcome variable until the COVID-19 outbreak; β_2 is the change in the level of outcome that occurred in the period immediately following the COVID-19 outbreak (compared with the counterfactual), and β3 is the difference between the pre-pandemic and pandemic slopes of the outcome. All statistical analyses were conducted using Stata 15.0 and SPSS 20.0.
Statistical methods	12	(*a*) Describe all statistical methods, including those used to control for confounding	Page 4 line 9-12	Interrupted time series (ITS) use multiple observations in the pre-intervention and post-intervention periods, and thus offer thereby offering a quasi-experimental research design with a potentially high degree of internal validity
		(*b*) Describe any methods used to examine subgroups and interactions	Page 4 line 8-10	Tt is the time since the start of the study, X_t is a dummy variable representing the intervention (pre-intervention period 0, otherwise 1), and X_tT_t is the interaction term.
		(*c*) Explain how missing data were addressed	N/A	
		(*d*) *Cohort study*—If applicable, explain how loss to follow-up was addressed<br>*Case-control study*—If applicable, explain how matching of cases and controls was addressed<br>*Cross-sectional study*—If applicable, describe analytical methods taking account of sampling strategy	N/A	

For peer review only - http://bmjopen.bmj.com/site/about/guidelines.xhtml

		(*e*) Describe any sensitivity analyses	N/A	
**Results**				
Participants	13*	(a) Report numbers of individuals at each stage of study—eg numbers potentially eligible, examined for eligibility, confirmed eligible, included in the study, completing follow-up, and analysed	N/A	There was no follow-up in our study .
		(b) Give reasons for non-participation at each stage	N/A	
		(c) Consider use of a flow diagram	N/A	
Descriptive data	14*	(a) Give characteristics of study participants (eg demographic, clinical, social) and information on exposures and potential confounders	Page 6 line 32-Page 7 line 5	We described healthcare service utilization in 2020 compared with 2019.
		(b) Indicate number of participants with missing data for each variable of interest	N/A	
		(c) *Cohort study*—Summarise follow-up time (eg, average and total amount)	N/A	
Outcome data	15*	*Cohort study*—Report numbers of outcome events or summary measures over time	Page 7 line 8-22	We described the percentage changes in healthcare-service utilization in Beijing.
		Case-control study—Report numbers in each exposure category, or summary measures of exposure	N/A	
		Cross-sectional study—Report numbers of outcome events or summary measures	N/A	
Main results	16	(*a*) Give unadjusted estimates and, if applicable, confounder-adjusted estimates and their precision (eg, 95% confidence	Page 8 line 2-Page 9 line 16	We described the results of the discontinuous time series

For peer review only - http://bmjopen.bmj.com/site/about/guidelines.xhtml

	interval). Make clear which confounders were adjusted for and why they were included	
	(*b*) Report category boundaries when continuous variables were categorized	N/A
	(*c*) If relevant, consider translating estimates of relative risk into absolute risk for a meaningful time period	N/A

Continued on next page

For peer review only

For peer review only - http://bmjopen.bmj.com/site/about/guidelines.xhtml

Other analyses	17	Report other analyses done—eg analyses of subgroups and interactions, and sensitivity analyses	Page 9 line 19-Page 10 line 4	We described the changes in online physician volume in 2020 compared with 2019.
**Discussion**				
Key results	18	Summarise key results with reference to study objectives	Page 10 line 7-20	Obvious complementary trends existed in online and face-to-face healthcare services during the COVID-19 pandemic. We found that public hospitals in Beijing experienced substantial losses in face-to-face healthcare and substantial increases in online-consultation services when the COVID-19 pandemic began in China in 2020. The interrupted time-series models revealed that after the COVID-19 outbreak, the considerable reduction in face-to-face healthcare services was a short-term effect, whereas the increase in online consultation was a long-term one.
Limitations	19	Discuss limitations of the study, taking into account sources of potential bias or imprecision. Discuss both direction and magnitude of any potential bias	Page 13 line 15-Page 13 line 27	This study had some limitations. First, we studied the complementary trend of online and face-to-face health service utilization. Whether the change in service delivery caused an impact on the quality or the equivalency of care delivery requires further study. Second, to observe the changes in healthcare-service utilization, the behaviour of patients with diseases of varied severity, i.e., AMI, LC, disk disease, and Parkinson's disease, was selected in this paper. Further studies are needed to determine whether this phenomenon can be extended to other diseases. The heterogeneity of the data across the hospitals was also not explored. Third, the usage of GoodDoctor cannot cover all the online-consultation behaviour. Finally, this study was only a snapshot capturing the first year of the pandemic response. Comprehensive research over a longer period is required.

For peer review only

For peer review only - http://bmjopen.bmj.com/site/about/guidelines.xhtml

Interpretation	20	Give a cautious overall interpretation of results considering objectives, limitations, multiplicity of analyses, results from similar studies, and other relevant evidence	Page 10 line 21-Page 13 line 14	The behaviour of patients with diseases of varied severity, i.e., AMI, LC, disk disease, and Parkinson's disease, was selected in this paper. Further studies are needed to determine whether this phenomenon can be extended to other diseases.
Generalisability	21	Discuss the generalisability (external validity) of the study results	Page 13 line 17-20	Further studies are needed to determine whether this phenomenon can be extended to other diseases.
**Other information**				
Funding	22	Give the source of funding and the role of the funders for the present study and, if applicable, for the original study on which the present article is based	Page 14 line 11-12	This study has been funded by the Natural Science Foundation of Beijing Municipality (grant number: 9222993).

*Give information separately for cases and controls in case-control studies and, if applicable, for exposed and unexposed groups in cohort and cross-sectional studies.

Note: An Explanation and Elaboration article discusses each checklist item and gives methodological background and published examples of transparent reporting. The STROBE checklist is best used in conjunction with this article (freely available on the Web sites of PLoS Medicine at http://www.plosmedicine.org/, Annals of Internal Medicine at http://www.annals.org/, and Epidemiology at http://www.epidem.com/). Information on the STROBE Initiative is available at www.strobe-statement.org.

For peer review only
